# Supplementary material for: Competing risk of mortality on loss to follow-up outcome among patients with HIV on ART: a retrospective cohort study from the Zimbabwe national ART programme
Source: BMJ Open. 2020 Oct 6;10(10):e036136. doi: 10.1136/bmjopen-2019-036136 (PMC7539573; doi:10.1136/bmjopen-2019-036136)
Supplement: Supplementary data [file bmjopen-2019-036136supp001.pdf]

A1: Cox proportional hazard

Traditionally for time-to-event analysis, researchers tend to fit the semi-parametric cause-specific proportional hazard model which was proposed by Cox (1972) because of its less stringent assumption on the hazard form and survival function [22]. For the conventional cause-specific proportion hazard, we did a non-informative censoring of LTFU and transfer-out. We assumed that the hazard function is an arbitrary, unspecified, non-negative function of time [22].

A2: Survival Function

Let *T* be a non-negative random variable defining the survival time of an individual. The survival function [*S(t)*] is used to represent the probability that an individual survives from time origin to some time beyond *t*

$$S(t) = P(T \geq t) \qquad (1)$$

If we assume that *T* is a continuous random variable $[P(T \geq t) = P(T > t)]$, the different values that *T* can take have an underlying probability density function (pdf) given by:

$$S(t) = P(T > t) = \int_0^t f(x)dx = 1 - F(t) \qquad (2)$$

where *F(t)* is the cumulative distribution function (CDF) expressed as:

$$F(t) = P(T < t) = \int_0^t f(x)dx \qquad (3)$$

If one has a survival function, the density function can be calculated by differentiating the cumulative distribution function as:

$$f(t) = -\frac{dF(t)}{dt} \qquad (4)$$

A3: The Hazard function

The hazard function is the probability that an individual dies at time *t* conditional to having survived to that time. The hazard function, therefore, represents an instantaneous death (failure) rate for an individual who survived to that time. In other words, the hazard function considers the probability that a random variable is associated with an individual's survival time lies between *t* and $t+\delta t$ conditional to *T* being greater than or equal to *t*:

$$\begin{aligned} h(t) &= P(t < T < t + \delta t \mid T \geq t) \\ &= \frac{F(t+\delta t) - F(t)}{S(t)} \end{aligned} \tag{5}$$

where *F(t)* is the cumulative distribution function. The hazard function is the limiting value of the above probability in Equation 5 divided by the time interval δt, as δt tends to zero. This can be re-written as:

$$\begin{aligned} h(t) = \mu(t) &= \lim_{\delta t \to 0} \frac{P[t \leq T < t + \delta t \mid T \geq t]}{\delta t} \\ &= \lim_{\delta t \to 0} \frac{P[t \leq T < t + \delta t]}{\delta t P(T > t)} \\ &= \lim_{\delta t \to 0} \frac{F[t + \delta t - F(t)]}{\delta t S(t)} \\ &= \frac{1}{S(t)} \left\{ \lim_{\delta t \to 0} \frac{F[t + \delta t - F(t)]}{\delta t} \right\} \end{aligned} \quad . \tag{6}$$

But $\left\{ \lim_{\delta t \to 0} \frac{F[t + \delta t - F(t)]}{\delta t} \right\} = f(t)$ and since T is a continuous random variable, then

$$h(t) = \frac{f(t)}{S(t)} = -\frac{d}{dt} \log[S(t)] \ . \tag{7}$$

The cumulative hazard is

$$H(t) = \int_0^t h(u)du = -\log[S(x)] \ . \tag{8}$$

Thus for a continuous lifetimes

$$\begin{aligned} S(t) &= \exp\{-H(t)\} \\ &= \exp\left[-\int_0^t h(u)du \right] \end{aligned} \ . \tag{9}$$

A4: The Cox regression model

This model is also known as the Proportional hazard regression model, which models the incidence or hazard rate (mortality rate in our case). This model assumes independence of

survival times between distinct individuals in a sample and a constant hazard ratio regardless of time. The model can be written as:

$$
\begin{aligned}
\log\left(\frac{h(t)}{h_0(t)}\right) &= \beta_1 X_1 + \beta_2 X_2 + \ldots + \beta_k X_k \\
\left(\frac{h(t)}{h_0(t)}\right) &= \exp(\beta_1 X_1 + \beta_2 X_2 + \ldots + \beta_k X_k) \\
h(t) &= h_0(t)\exp(\beta_1 X_1 + \beta_2 X_2 + \ldots + \beta_k X_k)
\end{aligned} \tag{10}
$$

This model can be simplified in a matric form as:

$$h(t/\mathbf{Z}) = h_0(t)\exp(\boldsymbol{\beta}\ \mathbf{Z}^T)$$

where $h(t/\mathbf{Z})$ is the expected hazard at time t conditional to the covariate matrix $\mathbf{Z}$. The expected hazard is a product of the baseline hazard ($h_0(t)$) (which is a function of duration time only) and the exponential function of the regressors ($\boldsymbol{\beta} = (\beta_1, \beta_2, \ldots, \beta_k)$; a 1 x k parameter vector) for k covariates. Then the explanatory variables for n persons, $\mathbf{Z}$ matrix, will be:

$$
\mathbf{Z} = \begin{bmatrix}
X_{11}, & X_{12}, & . & . & ., & X_{1n} \\
X_{21}, & X_{22}, & . & . & ., & X_{2n} \\
. & . & . & . & . & . \\
. & . & . & . & . & . \\
. & . & . & . & . & . \\
X_{k1}, & X_{k2}, & . & . & ., & X_{kn}
\end{bmatrix}
$$

Thus for the individual covariates, $Z_i = (X_{i1}, \ldots, X_{ik})^T$, the baseline hazard ($h_0(t)$ is equal to *h(t)* when all the regressors are equal to zero, the function of the regressors will be equal to 1. The Cox model is termed semi-parametric because it is made up of the parametric part for the covariates adjustment and the non-parametric baseline hazard as there are no assumptions for the underlying hazard function.

A5: Likelihood function of the Cox regression

The Cox model parameters are estimated by maximising the partial log-likelihood function. Letting the minimum censoring time C_j and survival time T_j be denoted by $t_{(j)}$. The censoring

time is right censored at C after observing that $T_j > C_j$. Let $R(t_j)$ denotes the set of lives which are at risk before the *j*th observed lifetime. Then the partial likelihood is a product of the event times of a quotient that compares the hazard of individuals with the event at a time $t_{(j)}$ to all the hazard of all individuals at risk at a time $t_{(j)}$ can be derived in terms of these censoring times. If we observe $\left\{\left(T_j, \delta_j\right)\right\}_{j=1}^{n}$ where T_j is still the observed time and δ_j the censoring indicator taking values 1 if the failure time is observed for individuals $j = 1, 2, ..., n$ where n is the total number of observations and 0 if right censoring, then the likelihood can be easily expressed as a product of the pdf and the cumulative survival function expressed as $LH \propto \prod_{j=1}^{n} f\left(t_j\right)^{\delta_j} S\left(t_j +\right)^{1-\delta_j}$. This function can be expressed as well as a partial likelihood function in terms of covariates and regression coefficients matrices, as shown below:

$$L_p(\beta \mid D_{obs}) = \prod_{j=1}^{n} \left[\frac{\exp(\boldsymbol{Z}_j^T \boldsymbol{\beta})}{\sum_{k \in R(t_j)} \exp(\boldsymbol{Z}_k^T \boldsymbol{\beta})} \right]^{\delta_j}, \tag{11}$$

where $D_{obs} = \{(t_j, \delta_j, \boldsymbol{Z}\ ) : j = 1, 2, ..., n\}$ is the observed univariate right-censored survival data. Considering the Breslow concept when ties are present, the partial likelihood may be expressed as:

$$L_{pt}(\beta \mid D_{obs}) = \prod_{j=1}^{d} \frac{\exp(\boldsymbol{Z}_j^T \boldsymbol{\beta})}{\left[\sum_{k \in R(t_j)} \exp(\boldsymbol{Z}_k^T \boldsymbol{\beta}) \right]^{d_j}},$$

where $d = \sum_{j=i}^{n} \delta_j, \boldsymbol{z}_j = \sum_{k \in D_j} \boldsymbol{x}_j, d_j =$ the number of events at $t_{(j)}$ and $R(t_j)$ is a set of all individuals who have the event at the defined time $t_{(j)}$. Thus, this can be written as

$$L_{pt}(\beta \mid D_{obs}) = \prod_{j=1}^{n} \frac{\exp(\delta_j \boldsymbol{Z}_j^T \boldsymbol{\beta})}{\left[\sum_{k \in R(t_j)} \exp(\boldsymbol{Z}_k^T \boldsymbol{\beta}) \right]^{\delta_j}},$$

Expanding the Breslow equation gives:

$$\log L(\beta \mid D_{obs}) = \sum_{j=1}^{D} \left[\sum_{i \in D_j}^{n} \boldsymbol{Z}_j^T \boldsymbol{\beta} - d_j \log \left\{ \sum_{k \in R_j}^{n} \exp(\boldsymbol{Z}_j^T \boldsymbol{\beta}) \right\} \right] \tag{12}$$

where *j* indexes the ordered failure times $t_{(j)}, j = 1,...,D$; D_j is the set of d_j observations that fail at time $t_{(j)}$; d_j is the number of failures at time $t_{(j)}$; and R_j is the set of observations *k* that are at risk at time $t_{(j)}$(that is, all k such that $t_{0k} < t_{(j)} \leq t_k$). This can be simplified in set notation form as $k \in R_j$ and $d_j \in D_j$.

A6: Competing risk model specification [49]

In epidemiological studies, more than one cause of failure is possible, hence the estimates of the targeted outcome probabilities are either over-estimated with the Cox model [50] the drive for the competing risk analysis to get marginal probabilities of mortality as proposed by Fine and Gray [17]. Competing risk is when we have more than one cause of failure outcomes, and we are only interested in one of them. In this case, mortality was the competing event to LTFU. The Kaplan-Meier estimate is normally used, where the competing risk events are treated as censored observations, but this is biased since the independence assumption is violated. An individual who is censored because of the competing event will certainly not experience the outcome of interest; hence, the Kaplan-Meier may overestimate the probability of failure, especially if completion is high. Hence, using the Kaplan-Meier estimate in this situation would result in the biased estimates. The competing risk inference is the joint distribution of time of death or failure (T) and failure outcome, in our case death (D) conditional to some covariates, X. The cause-specific hazard (*q* cause of death) function will be

$$h_q(t) = \lim_{\delta x \to 0} \frac{P(t \leq T < t + \delta t, D = q \mid T \geq t)}{\delta t} \tag{13}$$

The cumulative cause-specific hazard is written as:

$$H_q(t) = \int_0^t h_q(u) du \tag{14}$$

And define

$$S_q(t) = \exp(H_q(t)) \tag{15}$$

The cumulative incidence function (CIF) or the sub-distribution function for death is defined as:

$$I_q(t) = P(T \le t, D = q) = \int_0^t h_q(u) S(u) du \tag{16}$$

This function is sub-distribution since the cumulative probability of death is always below one, that is, $I_q(\infty) = P(D = q) < 1$. The hazard specification would be $h_q(t) = \frac{d \log(1 - I_q(t))}{dt}$, and the hazard model is expressed as

$$h_q(t / \mathbf{Z}) = h_{q,0}(t) \exp(\boldsymbol{\beta}_q \mathbf{Z}^T) \tag{17}$$

where $h_{q,0}$ is the baseline sub-distribution hazard of cause q and the vector $\boldsymbol{\beta}_q$ represents the matrix of covariates effects of cause q . To estimate the unknown $\boldsymbol{\beta}_q$ parameters, we maximise the following log-pseudolikelihood function:

$$\log L(\beta \mid D_{obs}) = \sum_{i=1}^{n} \delta_i w_i \left[\boldsymbol{Z}_i^T \boldsymbol{\beta} + offset_i - \log \left\{ \sum_{k \in R_i}^{n} w_i \alpha_{ik} \exp(\boldsymbol{Z}_k^T \boldsymbol{\beta} + offset_k) \right\} \right] \tag{18}$$

where δ_i is death for an ith individual, R_i is the set of observations, q at risk at time t_i. The only difference with the Cox is the α_{iq} weights. These weights keep individuals who experience the competing event to be in subsequent risk sets and to reduce their weight over time hence their likelihood of being censored increases.
