# Supplementary figures and images for: Competing risk of mortality on loss to follow-up outcome among patients with HIV on ART: a retrospective cohort study from the Zimbabwe national ART programme
Source: BMJ Open. 2020 Oct 6;10(10):e036136. doi: 10.1136/bmjopen-2019-036136 (PMC7539573; doi:10.1136/bmjopen-2019-036136)

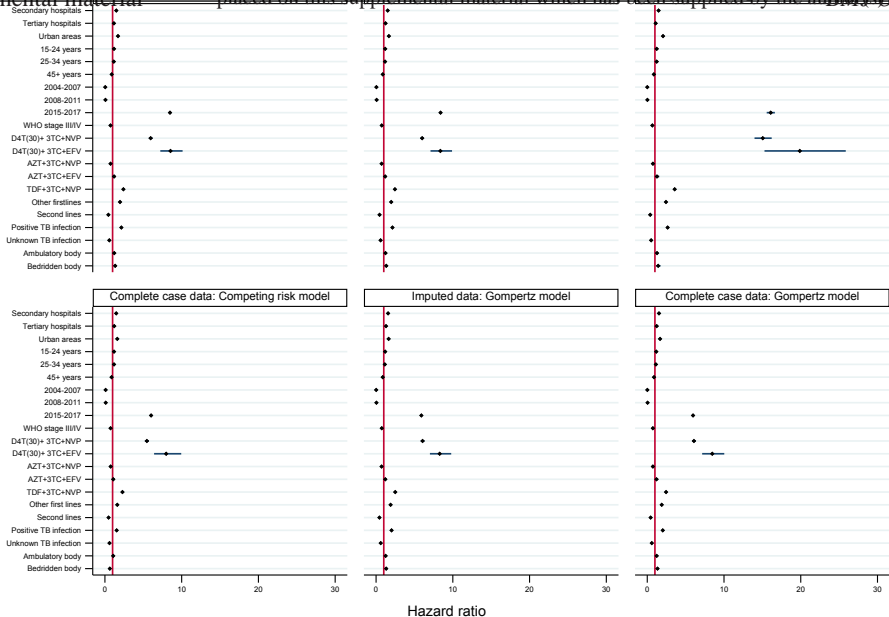

Supplement: Supplementary data [file bmjopen-2019-036136supp002.pdf]
