# Supplementary material for: Competing risk of mortality on loss to follow-up outcome among patients with HIV on ART: a retrospective cohort study from the Zimbabwe national ART programme
Source: BMJ Open. 2020 Oct 6;10(10):e036136. doi: 10.1136/bmjopen-2019-036136 (PMC7539573; doi:10.1136/bmjopen-2019-036136)
Supplement: Author's manuscript [file bmjopen-2019-036136.draft_revisions.pdf]

BMJ Open is committed to open peer review. As part of this commitment we make the peer review history of every article we publish publicly available.

When an article is published we post the peer reviewers' comments and the authors' responses online. We also post the versions of the paper that were used during peer review. These are the versions that the peer review comments apply to.

The versions of the paper that follow are the versions that were submitted during the peer review process. They are not the versions of record or the final published versions. They should not be cited or distributed as the published version of this manuscript.

BMJ Open is an open access journal and the full, final, typeset and author-corrected version of record of the manuscript is available on our site with no access controls, subscription charges or pay-per-view fees (http://bmjopen.bmj.com).

If you have any questions on BMJ Open's open peer review process please email info.bmjopen@bmj.com

BMJ Open

Competing risk of mortality on loss to follow-up outcome among HIV patients on ART: A retrospective cohort study from the Zimbabwe National ART Programme

Journal:	*BMJ Open*
Manuscript ID	bmjopen-2019-036136
Article Type:	Original research
Date Submitted by the Author:	02-Dec-2019
Complete List of Authors:	Matsena Zingoni, Zvifadzo; University of the Witwatersrand Faculty of Health Sciences, Division of Epidemiology and Biostatistcs; Ministry of Health and Child Care, National Institute of Health Research<br>Chirwa, Tobias; University of the Witwatersrand Faculty of Health Sciences, Division of Epidemiology and Biostatistics<br>Todd, Jim; LSHTM, EPH<br>Musenge, Eustasius; University of the Witwatersrand Faculty of Health Sciences, Division of Epidemiology and Biostatistics
Keywords:	Epidemiology < INFECTIOUS DISEASES, HIV & AIDS < INFECTIOUS DISEASES, Public health < INFECTIOUS DISEASES

SCHOLARONE™
Manuscripts

I, the Submitting Author has the right to grant and does grant on behalf of all authors of the Work (as defined in the below author licence), an exclusive licence and/or a non-exclusive licence for contributions from authors who are: i) UK Crown employees; ii) where BMJ has agreed a CC-BY licence shall apply, and/or iii) in accordance with the terms applicable for US Federal Government officers or employees acting as part of their official duties; on a worldwide, perpetual, irrevocable, royalty-free basis to BMJ Publishing Group Ltd ("BMJ") its licensees and where the relevant Journal is co-owned by BMJ to the co-owners of the Journal, to publish the Work in this journal and any other BMJ products and to exploit all rights, as set out in our licence.

The Submitting Author accepts and understands that any supply made under these terms is made by BMJ to the Submitting Author unless you are acting as an employee on behalf of your employer or a postgraduate student of an affiliated institution which is paying any applicable article publishing charge ("APC") for Open Access articles. Where the Submitting Author wishes to make the Work available on an Open Access basis (and intends to pay the relevant APC), the terms of reuse of such Open Access shall be governed by a Creative Commons licence – details of these licences and which Creative Commons licence will apply to this Work are set out in our licence referred to above.

Other than as permitted in any relevant BMJ Author's Self Archiving Policies, I confirm this Work has not been accepted for publication elsewhere, is not being considered for publication elsewhere and does not duplicate material already published. I confirm all authors consent to publication of this Work and authorise the granting of this licence.

For peer review only

Competing risk of mortality on loss to follow-up outcome among HIV Patients on ART: A retrospective cohort study from the Zimbabwe National ART Programme

Zvifadzo Matsena Zingoni[§, 1, 2], Tobias Freeman Chirwa[1], Jim Todd[3] and Eustasius Musenge[1]

[1]Division of Epidemiology and Biostatistics, University of the Witwatersrand, Johannesburg, South Africa

[2]National Institute of Health Research, Ministry of Health and Child Care, Harare, Zimbabwe

[3]Department of Population Health, London School of Hygiene and Tropical Medicine, London, United Kingdom

[§]Corresponding author: Zvifadzo Matsena Zingoni
27 St Andrews' Road
Parktown, 2193 Johannesburg, South Africa
email: zmatsena28@gmail.com
ORCID number: 0000-0002-7993-1187

Authors email addresses of authors:
Tobias Freeman Chirwa (TFC): Tobias.Chirwa@wits.ac.za
Jim Todd (JT): jim.todd@lshtm.ac.uk
Eustasius Musenge (EM): Eustasius.Musenge@wits.ac.za

Word count
Abstract: 299
Main body: 3975

ABSTRACT

Objective: To determine the effect of health facility type after ART services decentralisation on loss to follow up (LTFU) with mortality as a competing risk among patients.

Design: Retrospective cohort study

Setting: Nation-wide routinely collected HIV patients level data for monitoring ART outcomes in Zimbabwe. The data was collected at health facility level for patient who initiated ART between 2004 and 2017 through the electronic patient management system (ePMS).

Participants: The final sample considered had 390,771 participants from 538 health facilities aged 15 years and above extracted from the ePMS database.

For peer review only - http://bmjopen.bmj.com/site/about/guidelines.xhtml

Outcomes: The primary endpoint was LTFU defined as a failure of a patient to report for drug refill for at least 90 days from last appointment date or if the patient missed the next scheduled visit date and never showed up again. Mortality was considered a secondary outcome if a patient was reported to have died.

Results: The total exposure time contributed was 1,544,468 person-years. LTFU rate was 5.75(95% confidence interval (CI): 5.71-5.78) per 100 person-years. Adjustment for the competing event independently increased LTFU rate ratio in provincial and referral (adjusted sub-hazard ratios (AsHR) 1.24; 95%CI: 1.20-1.28) and district and mission (AsHR 1.47; 95%CI: 1.45-1.45) hospitals (reference: primary health care); in urban sites (AsHR 1.58; 95%CI: 1.55-1.60) (reference: rural); and among adolescence and young adults (15-24 years) group (AsHR 1.17; 95%CI: 1.15-1.19) (reference: 35-44 years). We also detected overwhelming association between LTFU and tuberculosis infected patients (AsHR 1.55; 95%CI: 1.47-1.64) (reference: no tuberculosis).

Conclusions: We have observed considerable findings that "leakages" within the ART treatment cascade still persist even after decentralisation of health services. Risk factors for LTFU reflect those found in sub-Saharan Africa studies. Interventions which retain patients in care by minimising any "leakages" along the treatment cascade are essential in attaining the UNAIDS 90-90-90 fast track targets.

Keywords: Antiretroviral therapy, Competing risk analysis, Loss to follow-up, Mortality, Retention, Risk factors,

Article Summary: Strength and limitations of the study

- The analysis is based on a huge national routinely collected individual-level dataset and a big follow-u period making our findings may be generalized to the ART population in Zimbabwe.
- Use of competing risk analysis based on the cumulative incidence function (CIF) to explore the association between selected covariates and the absolute risk which is essential for medical decision-making so that appropriate sub-population interventions can be implemented.

For peer review only - http://bmjopen.bmj.com/site/about/guidelines.xhtml

- Independent factors variable selection was performed using the regularization (shrinkage) lasso regression technique.
- Important factors known to influence LTFU like CD4 cell counts and viral load were missing in the dataset; and the risk factors adjusted for were not time dependent.
- Study has a possibility of participant selection bias since only participants enrolled in health facilities linked to the electronic patients management system were considered for analysis.

INTRODUCTION

Globally, remarkable progress has been made on the treatment of HIV-infected people using antiretroviral therapy(ART), particularly in sub-Saharan Africa(SSA)[1]. In Zimbabwe, the ART programme is a comprehensive care and support package aiming to slow disease progression[2], improve survival and quality of life [3]; and minimise HIV transmission by reducing the viral load. Therefore, it is imperative to monitor individuals on ART to ensure treatment efficacy, support adherence; and identify treatment failure and resistance.

At national level, aggregated health facility data is usually used for monitoring ART programme; however, rigorous analyses are restricted. This promoted the steady evolving of patient-level data worldwide[4–8]. With efforts to achieve zero HIV incidences by 2030, the 90-90-90 UNAIDS targets were launched, whereby 90% of the people living with HIV (PLWHIV) should know their status, 90% of those diagnosed should be initiated on ART, and 90% of those on ART should achieve viral suppression[9]. In Zimbabwe, strides have been made towards achieving these targets; however, the last 90% estimated at 73% in 2018 [10]. To improve the trailing last 90% indicator, it is crucial to come up with broad efforts which minimise long-term ART attrition due to loss to follow-up (LTFU), mortality, drop-outs and withdrawals.

In Zimbabwe, ART services were decentralised to increased coverage and easy access to those in need [11]. However, the impact ART decentralisation has on patients' outcomes, particularly in Zimbabwe using patients-level data in unknown. Reporting of ART outcomes at different health care facility types (primary health care (PHC), district/mission and provincial/referral hospitals) is vital to guide the ART programme and policy as well as reflecting on some of the gaps which may contribute to the tailing third 90% target. Therefore, this study aims to estimate the LTFU

For peer review only - http://bmjopen.bmj.com/site/about/guidelines.xhtml

rate by performing a competing risk analysis of mortality on LTFU outcome adjusting for health care facility types amongst ART patients; and determine other potential independent factors using patient-level data from the Zimbabwe National ART database.

METHODS

Study Population

This study was conducted in Zimbabwe, a landlocked country in SSA with roughly 1.4 million PLWHIV and HIV prevalence of 13.7% among adults in 2017[10]. Individual patients aged 15years and above, who initiated ART between 1st January 2004 and 31st December 2017, were eligible for this analysis. A full description of how the final sample size was achieved in illustrated on Figure 1.

[Insert Figure 1]

Study design and Data sources

This study was a secondary data analysis from a retrospective cohort study of routinely collected patient data through the electronic patient management system (ePMS). The data came from the national ART programme which started in 2004 with ART sites expanded to 1,566 by 2017[10]. Treatment guidelines followed WHO recommendations; hence, the eligibility criteria and first-line regimens changed over times. The initial regimen for an ART naïve patient is the first-line ART, a combination of two nucleoside reverse transcriptase inhibitors(NRTI), the first drug which can be Zidovudine(AZT), Tenofovir(TDF) (the most common) or Stavudine(D4T) and second drug is mainly Lamivudine (3TC); and one non-NRTI which can either be Nevirapine(NVP) or Efavirenz(EFV). Once the patient experienced treatment failure or intolerance, switch to second-line(a combination of protease inhibitor and two NRTIs) ART can be made[12].

Monitoring and Evaluation System

The monitoring and evaluation system of the ART programme in Zimbabwe has been described elsewhere[13]. Briefly, since the inception of ART, all patient monitoring records (PMR) have been collected manually using a paper-based system, kept at the health facility where the patient

For peer review only - http://bmjopen.bmj.com/site/about/guidelines.xhtml

is registered. At enrolment, the patient's demographic data are captured. At each subsequent clinic visit, information on ART regimen type, clinical information, laboratory investigations and physical examination is captured. Outcomes are assessed and captured as death, LTFU, transfer-out to another health facility or alive and active on treatment. In 2012 a national ePMS was introduced, with a retrospective entry of all existing PMR data at each clinic, to increase patient tracking and improve program monitoring. There was a systematic selection of the health facilities which represent all levels of care in Zimbabwe to implement ePMS. All subsequent clinic visits were recorded prospectively with a real-time entry of patient records.

Study Endpoints

The primary endpoint was LTFU defined as a failure of a patient to report for drug refill for at least 90 days from last appointment date or if the patient missed the next scheduled visit date and never showed up again. Patients, who returned after having been LTFU, were classified as alive and active on treatment. We also consider mortality outcome if a patient was reported to have died.

Data Analysis Methods

Descriptive summaries were reported as frequencies and proportions stratified by health facility type. Calendar trend pattern of LTFU was explored and incidence rates calculated from the ART initiation date to outcome occurrence date. Kaplan-Meier non-parametric curves (Equations 1-4 in Appendix) were used to describe the probability of LTFU over time stratified by selected covariates. The log-rank test was used to assess the equality of these patient retention probabilities. Estimated hazard rates (HR) (Equation 5-9 in Appendix) were reported as per 100 person-years.

Important variable selection was done using the lasso regression regularization technique. Variable selection sensitivity analysis was done using the subset selection techniques (best subset selection, stepwise forward variable selection and stepwise backward variable selection methods). All these methods yields the same independent factors to be assessed their association with time to LTFU using seven different time-to-event models were fitted, Figure 2. The predictors considered in the final regression models were all categorical namely: health facility

For peer review only - http://bmjopen.bmj.com/site/about/guidelines.xhtml

type, health facility site, age groups, tuberculosis status, body functional status, WHO clinical stage, calendar period and regimen type.

[Insert Figure 2]

The standard Cox regression model defined in Equation 10-12 in Appendix is normally used for time to event analysis; however, this method may give biased estimates in the presence of competing events. We fitted the competing risk (CR) model of mortality on LTFU through the cumulative incidence function (CIF) define in Equation 13-18 in Appendix) [14]. CR analysis assumes that patients are exposed to at least two risks (mortality and LTFU), of which one of the events is of interest (LTFU) while the other is a competing event (mortality) which inhibits the outcome of interest from occurring. The competing events may not be independent and the baseline hazard may differ between them. The covariates used were time independent; however, we adjusted for time to take into account the period the country experienced an economic turmoil, and the adjusted sub-hazard ratios (AsHR) were reported. We censored on 31st December 2017 for those who had not experienced the event of interest and at the last observed date for those who had transferred to other health facilities.

We performed a sensitivity analysis of our key findings through multiple imputations of the missing explanatory covariates, assuming Bayesian estimation (BE) and maximum likelihood estimation (MLE) for Gombertz parametric models and fitting parametric Weilbull model with individual random effects. Model comparison between BE and MLE was based on the Akaike's information criterion (AIC), Bayesian information criterion (BIC) and Deviance information criterion (DIC) while model comparison between complete case analysis and imputed data analysis was based on the precision of the confidence interval for the estimates. All data management and analysis were performed using Stata 15.1 (StataCorp, 2017, Stata Statistical Software: Release 15.1, College Station, TX: StataCorp LLC)[15].

Patient and Public involvement

This study acknowledges the importance of patients and public involvement in research studies; however, this research was done without patient involvement on all research stages since it was a secondary data analysis.

For peer review only - http://bmjopen.bmj.com/site/about/guidelines.xhtml

Ethical Approval

Ethics approval was granted by The University of Witwatersrand's Human Research Ethics Committee (Medical) (Clearance Certificate No. M170673). Data approval was obtained from the Ministry of Health and Child Care who are the custodians of the countrywide database. Since this study was a secondary data analysis, we did not have direct contact with the participants. We received only anonymized patients' data for analyses.

RESULTS

Descriptive Analysis

At the end of study 268,896 (68.81%) patients were alive, 18,328(4.7%) had died, 88,744 (22.71%) were LTFU and 14,803(3.79%) had transferred out. Over 1,544,468 person-years of ART, mortality and LTFU rates were 1.19(95%CI: 1.17-1.20) and 5.75(95%CI: 5.71-5.78) per 100 person-years, respectively. The trend analysis graph of LTFU over time showed a general increase in LTFU over time with significant peaks in 2012, 2014 and 2016 (Figure 3).

[Insert Figure 3]

The descriptive characteristics of 390,771 patients from 538 health facilities are presented in Table 1**.** Patients were enrolled at PHC (45.1%), districts/mission (49.2%) or provincial/referral (5.7%) hospitals. Most patients were enrolled in rural health facilities (66.82%). At ART initiation, majority of the patients were female 117,567(66.7%) with mean (standard deviation) age of 37.5(10.2) years and weight of 60.2(12.3) Kg. Most patients were married (59.5%) with secondary education (31.8%), in WHO stage III/IV (54.2%) and within 35-44 years age group (34.18%). Patients were mainly on first-line ART treatment (97.7%) and few had tuberculosis infection (1.2%). There was missing data on some covariates including baseline CD4 cell count which was only available in 35.3% of the patients**.**

Table 1: Patients sociodemographic and clinical characteristics at ART initiation in Zimbabwe national ART programme, 2004-2017

Characteristics	Categories	Primary health care (PHC) n=176 253 (45.1%)	District/Mission hospitals n=192 238(49.2%)	Provincial/Referral hospitals n=22 280 (5.7%)	Totals N=390 771 (100%)
**Patient Demographics**					

For peer review only - http://bmjopen.bmj.com/site/about/guidelines.xhtml

Age (years) at ART initiation	15-24	20 314 (11.53%)	21 057 (10.95%)	2 601 (11.67%)	43 972 (11.25%)
	25-34	54 416 (30.87%)	58 874 (30.63%)	6 979 (31.32%)	120 269 (30.78%)
	35-44	59 284 (33.64%)	66 418 (34.55%)	7 875 (35.35%)	133 577 (34.18%)
	45+	42 239 (23.96%)	45 889 (23.87%)	4 825 (21.66%)	92 953 (23.79%)
Degree of urbanization	Rural	113 738 (66.92%)	137 380 (73.57%)	432 (2.01%)	251 550 (66.52%)
	Urban	56 223 (33.08%)	49 348 (26.43%)	21 055 (97.99%)	126 626 (33.48%)
Sex	Females	117 567 (66.70%)	124 141 (64.58%)	14 136 (63.45%)	255 844 (65.47%)
	Male	58 686 (33.30%)	68 097 (35.42%)	8 144 (36.55%)	134 927 (34.53%)
Education status at ART initiation	None	3 854 (2.19%)	4 136 (2.15%)	227 (1.02%)	8 217 (2.10%)
	Primary	33 735 (19.14%)	34 062 (17.72%)	2 248 (10.09%)	70 045 (17.92%)
	Secondary	59 181(33.58%)	58 349 (30.35)	6 593 (29.59%)	124 123 (31.76%)
	Tertiary	2 921(1.66%)	4 423(2.30%)	1 177(5.28%)	8 521 (2.18%)
	Missing	76 562(43.44%)	91 268 (47.48%)	12 035(54.02)	179 865 (46.03%)
Marital status at ART initiation	Single	24 438 (13.87%)	24 489 (12.74%)	3 530 (15.84%)	52 457 (13.42%)
	Married	105 504 (59.86%)	113 953 (59.28%)	12 935 (58.06%)	232 392 (59.47%)
	Widowed	25 798 (14.64%)	30 994 (16.12%)	3 523 (15.81%)	3 523 (15.81%)
	Divorced	13 429 (7.62%)	14 732 (7.66%)	1 677 (7.53%)	29 838 (7.64%)
	Missing	7 084(4.02%)	8 070 (4.20%)	615 (2.76%)	15 769 (4.04%)
Calendar time period of starting ART	2004-2007	6 635 (3.76%)	8 942 (4.65%)	2 962 (13.29%)	18539 (4.74%)
	2008-2011	38 990 (22.12%)	61 321 (31.85%)	7 899 (35.45%)	108120 (27.67%)
	2012-2014	67 759 (38.44%)	90 582 (47.12%)	9 472 (42.51%)	167 813 (42.94%)
	2015-2017	62 869 (35.67%)	31 483 (16.38%)	1 947 (8.74%)	96 299 (24.64%)
**Clinical factors**					
WHO staging at ART initiation	I/II	82 203 (46.64%)	69 629 (36.22%)	7 690 (34.52%)	159 519 (40.82%)
	III/IV	79 279 (44.98%)	118 113 (61.44%)	14 425 (64.74%)	211 817 (54.20%)
	Missing	14 771 (8.38%)	4 499 (2.34%)	165 (0.74%)	19 435 (4.97%)
Baseline CD4 (cell/uL) at ART initiation	Above 500	4 783 (2.71%)	4 950 (2.57%)	601 (2.70%)	10 334 (2.64%)
	351-500	8 227 (4.67%)	8 790 (4.57%)	940 (4.22%)	17 957 (4.60%)
	200-350	17 908 (10.16%)	24 895 (12.95%)	2 866 (12.86%)	45 669 (11.69%)
	Below 200	23 796 (13.50%)	35 020 (18.22%)	4 982 (22.36%)	63 798 (16.33%)
	Missing	121 539 (68.96%)	118 583 (61.69%)	12 891 (57.86%)	253 013 (64.75%)
Treatment regimen at time of data extraction	First line	170 662 (98.29%)	184 321 (97.37%)	20 724 (94.32%)	375 704 (97.65%)
	D4T(30)+3TC+NVP	625 (0.35%)	2 942 (1.53%)	629 (2.82%)	4 196 (1.07%)
	D4T(30)+ 3TC+EFV	42 (0.02%)	155 (0.08%)	20 (0.09)	217(0.06%)
	AZT+3TC+NVP	1 349 (0.77%)	1 933 (1.01%)	349 (1.57%)	3 631(0.93%)
	AZT+3TC+EFT	212 (0.12%)	425 (0.22%)	56 (0.25 %)	693 (0.18%)
	TDF+3TC+NVP	6 541 (3.71%)	16 334 (8.50%)	2 923 (13.12%)	25 798 (6.60%)
	TDF+3TC+EFV	161 288 (91.51%)	162 006 (84.27%)	16 628 (4.63%)	339 922 (86.99%)
	Other first lines	3 236 (1.84%)	3 474 (1.81%)	428 (1.92%)	7 138 (1.83%)
	Second line	2 960 (1.68%)	4 969 (2.58%)	1 247(5.60%)	9 176 (2.35%)
Tuberculosis status at ART initiation	Negative	156 072 (88.55%)	170 869 (88.88%)	20 218 (90.75%)	347 159 (88.84%)
	Positive	2 092 (1.19%)	2 423 (1.26%)	301 (1.35%)	4 816 (1.23%)
	Not assessed	15 743 (8 93%)	16 387 (8.52%)	1 347 (6.05%)	33 477 (8.57%)
	Missing	2 346 (1.33%)	2 559 (1.33%)	414 (1.86%)	5 319 (1.36%)
Functional status at ART initiation	Working	162 710 (92.32%)	180 417 (93.85%)	20 024 (89.87%)	363 151 (92.93%)
	Ambulatory	11 789 (6.69%)	10 454 (5.44%)	2 089 (9.38%)	24 332 (6.23%)
	Bedridden	331 (0.19%)	452 (0.24 %)	28(0.13%)	811 (0.21%)
	Missing	1 423 (0.81%)	915 (0.48%)	139(0.62%)	2 477 (0.63%)

The time to event rates for LTFU stratified by baseline characteristics are shown in Table 2. LTFU rate per 100-person-years was 5.92 for patients enrolled at district and mission hospitals; 6.60 amongst patients from rural health facilities and 17.09 among patients diagnosed with tuberculosis.

For peer review only

For peer review only - http://bmjopen.bmj.com/site/about/guidelines.xhtml

Table 2: Rates per 100 person-years of loss to follow-up and log rank tests for ART patients' baseline characteristics in Zimbabwe national ART programme, 2004-2017

Characteristics	Category	LTFU cases	Person-years	Rate per 100 person-years	95% CI of rate	Log rank p-value
Health facility level	Primary health care	34 958	6 200	5.6502	5.5913 to 5.7097	
	District/mission hospitals	48 188	8 100	5.9169	5.8643 to 5.9700	<0.001*
	Provincial/referral hospitals	5 598	1 100	5.0273	4.8973 to 5.1608	
Age (years) at ART initiation	15-24	32 790	4 500	7.3252	7.2463 to 7.4049	
	25-34	12 617	1 500	8.4301	8.2843 to 8.5785	
	35-44	27 504	5 500	4.9671	4.9087 to 5.0261	<0.001*
	45+	15 833	3 900	4.0242	3.9620 to 4.0874	
Degree of urbanization	Rural	52 629	10 000	5.2756	5.2307 to 5.3209	
	Urban	36 115	5 500	6.6039	6.5362 to 6.6724	<0.001*
Sex	Females	59 695	10 000	5.8703	5.8234 to 5.9175	
	Male	29 049	5 300	5.5063	5.4433 to 5.5700	<0.001*
Education status at ART initiation	None	1 470	354	4.1582	3.9509 to 4.3762	
	Primary	14 786	2 800	5.3558	5.2702 to 5.4429	
	Secondary	32 629	4 700	6.9961	6.9206 to 7.0725	<0.001*
	Tertiary	1 803	409	4.4097	4.2108 to 4.6180	
	Missing	38 056	7 300	5.2435	5.1911 to 5.2965	
Marital status at ART initiation	Single	13 078	1 900	6.9036	6.7863 to 7.0230	
	Married	7 548	1 100	6.6112	6.4637 to 6.7620	
	Widowed	9 375	2 900	3.1872	3.1233 to 3.2524	<0.001*
	Divorced	55 985	8 800	6.3313	6.2790 to 6.3839	
	Missing	2 758	625	4.4160	4.2543 to 4.5840	
Calendar time period of starting ART	2004-2007	790	1 900	0.4255	0.3968 to 0.4562	
	2008-2011	4 572	6 900	0.6593	0.6405 to 0.6787	
	2012-2014	43 565	5 300	8.1802	8.1038 to 8.2574	<0.001*
	2015-2017	39 817	1 300	29.9903	29.6972 to 30.2863	
WHO staging at ART initiation	I/II	47 410	5 100	9.2080	9.1255 to 9.2912	
	III/IV	38 919	9 500	4.0957	4.0552 to 4.1366	<0.001*
	Missing	2 415	794	3.0432	2.9242 to 3.1671	
Baseline CD4 (cell/uL) at ART initiation	Above 500	4 341	257	16.9190	16.4231 to 17.4299	
	351-500	7 614	455	16.7362	16.3645 to 17.1164	
	200-350	11 556	1 700	6.7428	6.6210 to 6.8669	<0.001*
	Below 200	15 271	2 700	5.7338	5.6436 to 5.8254	
	Missing	49 962	10 000	4.8245	4.7823 to 4.8669	
Treatment regimen at time of data extraction	First line	88 050	15 000	5.9066	5.8677 to 5.9457	
	D4T(30)+ 3TC+NVP	2 685	167.9730	15.9847	15.3914 to 16.6009	
	D4T(30)+ 3TC+EFV	141	10	14.7353	12.4932 to 17.3797	
	AZT+3TC+NVP	448	184	2.4374	2.2218 to 2.6738	
	AZT+3TC+EFT	158	30	5.3159	4.5484 to 6.2129	<0.001*
	TDF+3TC+NVP	9 652	1 100	9.1737	8.9925 to 9.3586	
	TDF+3TC+EFV	71 378	13 000	5.3710	5.3318 to 5.4106	
	Other first lines	3 588	175	20.5590	19.8972 to 21.2429	
	Second line	694	538	1.2900	1.1985 to 1.3908	
Tuberculosis status at ART intiation	Negative	81 119	14 000	5.9418	5.9010 to 5.9828	
	Positive	2 235	131	17.0906	16.3966 to 17.8141	
	Not assessed	4 188	1 400	2.9328	2.8453 to 3.0230	<0.001*
	Missing	1 202	234	5.1455	4.8627 to 5.4447	
Functional status at ART initiation	Working	81 122	14 000	5.6445	5.6057 to 5.6834	
	Ambulatory	7 059	930	7.5897	7.4147 to 7.7688	
	Bedridden	215	26	8.4201	7.3666 to 9.6243	<0.001*
	Missing	348	117	2.9723	2.6758 to 3.3015	

*Significant at 5%

For peer review only

For peer review only - http://bmjopen.bmj.com/site/about/guidelines.xhtml

The cumulative incidence probability (CIP) curve for mortality outcome was lower compared to the LTFU outcome (Figure 4A). The LTFU CIP curve increased in the first 3 years on ART and became steady thereafter. We observed overwhelmingly significant variation between LTFU and selected covariates, log-rank p-value<0.001. Higher risk of LTFU was observed amongst 15-24 years patients (Figure 4B), females (Figure 4C), patients enrolled at urban health facilities (Figure 4D); tuberculosis infected patients (Figure 4E) and WHO stage I/II patients (Figure 4F).

[Insert Figure 4]

Loss to Follow-up Outcome

To determine the independent risk factors associated with LTFU, seven models were fitted and the results are shown in Table 3. The data had approximately 10% of missing information in the explanatory covariates. There was no difference in the model estimates between imputed data and complete case data (model 1 vs model 2 and model 4 vs model 5). Comparing the MLE (model 3) and the BE (model 4) models, the estimates were almost similar for most regression parameters; and inclusion of the individual random effects did not improve the model estimates (model 4 vs model 6). Lastly, the competing risk model gave a better fit compared to other models.

The multivariable model revealed that patient factors predicting LTFU included: ART initiation at both provincial/referral (AsHR 1.22; 95%CI: 1.18-1.26) and district/mission (AsHR 1.47; 95%CI: 1.45-1.50) hospitals (reference: PHC), urban sites (AsHR 1.61; 95%CI:1.59-1.63) (reference: rural) and 15-24 years group (AsHR 1.19; 95%CI: 1.16-1.21) (reference: 35-44 years). We also detected overwhelming association between LTFU and tuberculosis infected patients (AsHR 1.53; 95%CI: 1.45-1.62) (reference: no tuberculosis) and a protective effect of LTFU for patients in WHO stage III/IV (AsHR 0.74; 95%CI: 0.73-0.75) (reference: WHO I/II). Patients who were on first-line ART treatment taking stavudine-combination therapy, i.e. D4T(30)+3TC+NVP (AsHR 5.47; 95%CI:5.52-5.75) or D4T(30)+3TC+EFV (AsHR 7.98; 95%CI: 6.41-9.94), had an increased risk of becoming LTFU relative to patients taking TDF+3TC+EFV combination.. We also observed that patients who initiated ART between 2015 and 2017 had a pronounced risk of becoming LTFU compared to patients who enrolled between 2012 and 2014 (AsHR 6.02; 95%CI: 5.91-6.12).

For peer review only - http://bmjopen.bmj.com/site/about/guidelines.xhtml

Table 3: Multiple regression models results of LTFU amongst ART patients in Zimbabwe national ART programme, 2004-2017 for different models

**Characteristics**	**Category**	**Cox proportional hazard**		**Parametric Gompertz model**			**Weibull model with random effects**	**Competing risk model of mortality on LTFU**
		**CC + MLE (model 1)**	**MI+ MLE (model 2)**	**CC+BE (model 3)**	**CC+MLE (model 4)**	**MI+MLE (model 5)**	**CC+MLE (model 6)**	**CC+MLE (model 7)**
Health facility level	Primary health care	1(reference)	1(reference)	1(reference)	1(reference)	1(reference)	1(reference)	1(reference)
	Secondary hospitals[a]	1.48(1.46-1.50)	1.51(1.49-1.53)	1.53(1.51-1.54)	1.53(1.51-1.55)	1.57(1.54-1.59)	1.48(1.45-1.51)	1.47(1.45-1.50)
	Tertiary hospitals[b]	1.17(1.14-1.21)	1.23(1.20-1.27)	1.23(1.21-1.25)	1.23(1.20-1.27)	1.30(1.26-1.34)	1.10(1.06-1.14)	1.22(1.18-1.26)
Age (years) at ART initiation	15-24	1.16(1.13-1.19)	1.17(1.14-1.19)	1.12(1.11-1.14)	1.13(1.10-1.15)	1.14(1.12-1.17)	1.23(1.20-1.27)	1.19(1.16-1.21)
	25-34	1.18(1.17-1.20)	1.19(1.17-1.21)	1.18(1.16-1.19)	1.18(1.16-1.20)	1.19(1.17-1.20)	1.24(1.21-1.27)	1.19(1.17-1.21)
	35-44	1(reference)	1(reference)	1(reference)	1(reference)	1(reference)	1(reference)	1(reference)
	45+	0.88(0.87-0.89)	0.87(0.86-0.89)	0.88(0.87-0.89)	0.88(0.86-0.90)	0.87(0.85-0.88)	0.86(0.84-0.88)	0.87(0.85-0.89)
Degree of urbanization	Rural	1(reference)	1(reference)	1(reference)	1(reference)	1(reference)	1(reference)	1(reference)
	Urban	1.70(1.68-1.72)	1.67(1.65-1.27)	1.67(1.65-1.69)	1.67(1.65-1.70)	1.64(1.61-1.66)	2.06(2.01-2.10)	1.61(1.59-1.63)
Calendar time period of starting ART	2004-2007	0.04(0.04-0.05)	0.04(0.04-0.05)	0.02(0.02-2.02)	0.02(0.01-0.02)	0.02(0.01-0.02)	0.01(0.01-0.01)	0.09(0.09-0.10)
	2008-2011	0.07(.0.7-0.08)	0.07(0.07-0.08)	0.04(0.04-0.04)	0.04(0.04-0.04)	0.05(0.04-0.05)	0.02(0.02-0.02)	0.11(0.10-0.11)
	2012-2014	1(reference)	1(reference)	1(reference)	1(reference)	1(reference)	1(reference)	1(reference)
	2015-2017	8.48(8.34-8.63)	8.40(8.26-8.54)	5.95(5.90-6.00)	5.97(5.88-6.06)	5.90(5.81-5.99)	16.09(15.56-16.63)	6.02(5.91-6.12)
WHO staging at ART initiation	I/II	1(reference)	1(reference)	1(reference)	1(reference)	1(reference)	1(reference)	1(reference)
	III/IV	0.73(0.72-0.74)	0.74(0.73-0.75)	0.73(0.73-0.74)	0.73(0.72-0.75)	0.74(0.73-0.75)	0.67(0.65-0.68)	0.74(0.73-0.75)
Treatment regimen at time of data extraction	D4T(30)+ 3TC+NVP	5.96(5.72-6.21)	6.00(5.77-6.25)	6.065.92-6.22)	6.09(5.84-6.34)	6.06(5.82-6.30)	15.05(13.98-16.21)	5.47(5.52-5.75)
	D4T(30)+ 3TC+EFV	8.55(7.21-10.12)	8.34(7.09-9.90)	8.71(8.21-9.20)	8.47(7.16-10.03)	8.29(7.01-9.79)	19.88(15.23-25.87)	7.98(6.41-9.94)
	AZT+3TC+NVP	0.73(0.67-0.80)	0.72(0.66-0.79)	0.74(0.71-0.78)	0.74(0.67-0.81)	0.72(0.65-0.79)	0.74(0.66-0.83)	0.75(0.68-0.83)
	AZT+3TC+EFT	1.19(1.02-1.40)	1.19(1.01-1.39)	1.19(1.16-1.23)	1.21(1.03-1.42)	1.21(1.03-1.40)	1.27(1.03-1.56)	1.09(0.91-1.30)
	TDF+3TC+NVP	2.41(2.36-2.47)	2.46(2.40-2.51)	2.46(2.42-2.50)	2.45(2.40-2.51)	2.50(2.44-2.55)	3.58(3.45-3.70)	2.29(2.23-2.34)
	TDF+3TC+EFV	1(reference)	1(reference)	1(reference)	1(reference)	1(reference)	1(reference)	1(reference)
	Other first lines	1.97(1.90-2.04)	1.97(1.91-2.04)	1.88(1.83-1.93)	1.89(1.82-1.95)	1.89(1.83-1.96)	2.44(2.32-2.57)	1.61(1.54-1.68)
	Second line	0.46(0.42-0.49)	0.45(0.42-0.49)	0.45(0.43-0.47)	0.44(0.41-0.48)	0.44(0.41-0.47)	0.39(0.36-0.42)	0.48(0.44-0.52)
Tuberculosis status at ART initiation	Negative	1(reference)	1(reference)	1(reference)	1(reference)	1(reference)	1(reference)	1(reference)
	Positive	2.14(2.05-2.24)	2.13(2.04-2.23)	2.03(1.99-2.09)	2.02(1.94-2.11)	2.01(1.93-2.10)	2.66(2.50-2.83)	1.53(1.45-1.62)
	Not assessed	0.58(0.56-0.59)	0.59(0.57-0.61)	0.60(0.59-0.60)	0.60(0.58-0.62)	0.61(0.59-0.63)	0.51(0.49-0.53)	0.60(0.58-0.62)
Functional status at ART initiation	Working	1(reference)	1(reference)	1(reference)	1(reference)	1(reference)	1(reference)	1(reference)
	Ambulatory	1.22(1.19-1.24)	1.23(1.20-1.26)	1.23(1.21-1.26)	1.23(1.20-1.27)	1.25(1.22-1.28)	1.28(1.23-1.32)	1.08(1.05-1.11)
	Bedridden	1.34(1.17-1.53)	1.33(1.16-1.52)	1.32(1.29-1.35)	1.34(1.16-1.53)	1.32(1.15-1.51)	1.42(1.18-1.72)	0.64(0.54-0.75)
	AIC	1964164			379948.9		445559.7	**199708.7**
	BIC	1964391			380197.6		445819.3	**199731.4**
	DIC			379925.9				

CC=complete case, MI=multiple imputation, BE=Bayesian estimation, MLE=maximum likelihood estimation, Bold face= low values of the information criterion

[a] District and mission hospitals; [b] Provincial and referral hospitals

For peer review only

DISCUSSION

This study aims to describe the competing risk of mortality on LTFU outcome adjusting for health care facility types amongst ART patients; and determine other potential independent factors using patient-level data from the Zimbabwe national ART database.

Our study followed similar studies adjusting for the competing effect of mortality on LTFU, recognising that those who die are no longer at risk of LTFU [16,17]. We found LTFU rate of 5.7 per 100- person-years which was lower than that reported in Ethiopia of 8.2 per 100 person-years [18]. However, these findings show a comparable and a considerable magnitude of LTFU outcome in ART programs which requires upscaling of interventions to retain patients since LTFU is a problem which may leads to ART resistance. We also observed a lower mortality rate per 100 person-years of 1.19 compared to 8.0 reported in Zambia [19]. This may be attributed to under reporting of mortality outcomes to ART clinics by family or friends; hence, poor unaccountability of death outcome. Moreover, participant selection bias during the ePMS data compilation process might have occurred resulting in the exclusion of those patients who had died.

Similar to other studies, LTFU independently increased at higher levels of care relative to PHC[5]. This could be explained by "silent transfers" to closer health facilities with cheaper transport cost and less congested environments[20]. However, in contrary higher levels of care facilities may receive much sicker patients with a high risk of death hoping to get better treatment through the health care referral system [21]. A similar study from this same cohort observed that much sicker patients with severe immune deterioration were more likely to become LTFU compared to those with no significant immunodeficiency [22]. Therefore, some of the reported LTFU in higher levels of care may be misclassification due to "silent transfers" or deaths among much ill patients through the referral system [23]. Patients enrolled at urban health facilities had increased risk to become LTFU compared to those at rural sites. This finding contradicts a study done in Ethiopia whereby rural residents had an increased risk of LTFU due to availability of transport costs, long distances to ART health facilities, social stigma as well as low levels of HIV knowledge in these communities[24]. However, our study finding can be explained by the decentralisation of health facilities to walkable distances in favour of rural

For peer review only - http://bmjopen.bmj.com/site/about/guidelines.xhtml

residents [4,25]. Mobility of residents in urban areas is disproportionate relative to rural residents; hence, most of these LTFU in urban areas could be part of the silent transfers to other nearer ART health facilities in their relocated areas.

Another interesting finding was high LTFU among the 15-24 years and 25-34 years age groups. The adolescents group is known to be associated with additional intricacy in their HIV management due to different types of chronic comorbidities; and this whole complexity may impact on their ART outcomes [26,27]. Adolescents are also much more vulnerable, often negatively affected by HIV-related stigma and discrimination; hence, it is difficult for this group to discuss HIV issues [28]. Interventions such as community-based support for parents or guardians have been found to be effective for these adolescents groups to help improve ART outcomes [29]. The high LTFU risk among the 25-34 years age group could be explained by lack income to foot travel and other indirect ART treatment costs [28] in addition to psychological distress.

We observed an overwhelming association of tuberculosis infection and becoming LTFU which concurs with an earlier study which reported that the risk of becoming LTFU was two times more likely in HIV patients with tuberculosis infection compared to those who did not have tuberculosis [18]. This finding can be explained by overlapping toxicity of HIV and tuberculosis drugs leading to adverse reactions which have a direct consequence to becoming LTFU [18]. Therefore, a push towards integrated care of HIV and tuberculosis service delivery would elevate HIV uptake and tuberculosis screening and reduce travel inconveniences on patients [21,30]. Also, tuberculosis treatment before ART initiation reduces the risk to LTFU [31]; hence, it is profound to increase efforts in tuberculosis screening and treatment of HIV patients. However, issues around tuberculosis drug resistance cannot be ignored. Nowadays, HIV studies also consider information on non-communicable diseases like cancer, diabetes and hypertensition; however, this information was missing from this current study database.

We also detected that patients in WHO stage III/IV were less likely to become LTFU relative to those with WHO stage I/II. However, our finding contradict with earlier studies which have reported that experiencing a severe AIDS-defining ailment was associated with reduced risk of becoming LTFU[16,32]. Our study finding may put forward the fact that there could have been

For peer review only - http://bmjopen.bmj.com/site/about/guidelines.xhtml

an upsurge of health-seeking behaviour amongst patients with advanced WHO clinical stages in this database cohort or this finding could be attributed to expansions of community awareness through health promotion in these health facilities communities.

Strengths and weaknesses of this study

This study analyses employed have some implications; we used the regularization variable selection technique to identify the important covariates to be used. The lasso model shrinks the variable coefficient to zero and produces a simpler model which can be interpreted easily. For the survival model, various model types were fitted including the standard Cox regression model; however, due to its overestimation of the risk in the presence of competing risks, we considered the Fine and Gray competing risk model [33]. We have seen that ignoring competing risks alters the probabilities of occurrence of the outcome giving biased estimates. Standard survival methods normally used in many studies censor the competing risk; however, this is improper because those who have died can no longer become LTFU. Such analysis will either overestimate or underestimate the risk ratios. We also used individual data which enabled exploration and adjustment for patient-level covariates associated with outcome to get robust information on the ART outcome patterns as compared to aggregated data[19,34]. The follow-up period in this study was relatively long (13years), and the dataset used was huge represented all the ten provinces in Zimbabwe without restriction to specific geographical areas. The data represented quaternary, tertiary, secondary and primary health facilities from private and public sectors. This makes our results be generalizable to the Zimbabwe setting since the data reflect a true representation of all HIV structured in the country. To the best of our knowledge, this study could be one of the largest dataset used for Zimbabwe to analyse adult ART outcomes at the patient level.

Our study results should be inferred in light of some limitations. Missing data was of concern, particularly to those variables that influence LTFU like viral load and CD4 cell count in addition to non-communicable diseases comorbidities. The presents of missing data could be explained by poor documentation of information [21] or limited access to the point of care machines for viral load and CD4 cell counts measurements [4,35,36]. The missing patterns for some covariates like viral load and CD4 cell counts measurements was likely to be informative and missing not at

For peer review only - http://bmjopen.bmj.com/site/about/guidelines.xhtml

random (MNAR) since differentiated care monitoring approach was used in some years covered by this study[37]. Therefore, in order to maintain a sufficient power, covariates with high percentage of missing observations were excluded at the multivariable analysis stage. In future, we recommend that the data clerks should be trained on the importance of capturing full information and to the programme managers to consider expanding the HIV routine data collection tool to capture non-communication diseases conditions of the HIV patients. The socio-demographic and clinical characteristics in the database were only taken at baseline or last observed at time of data extraction; hence, we could not incorporate the time dependency of some covariates. We also acknowledge that there could be differential misclassification of the LTFU outcome in this study since some patients might have silently transferred to other health facilities as a result of ART decentralisation; hence, this might have biased our LTFU estimates in this study. There is also a possibility of participant selection bias since we only considered patients whose information as captured through the ePMS; as a results the reported findings could be systematically different from those patients not included. However, it is crucial to note that the dataset used was very huge and representation of the health facilities in Zimbabwe was reflected in this dataset; hence, our findings could be generalized to all ART services health facilities in Zimbabwe.

While our findings for the common predictors of LTFU may be comparable to earlier studies conducted in Sub- Saharan Africa region, we had also hypothesised that those ART patients' who enrolled around 2008 would have a higher risk of becoming LTFU. This was done taking note of the hyperinflation that occurred in Zimbabwe during the part of the study period under review. The economic turmoil was allied to the increased mass departure of qualified health professionals and scarcities of vital medical supplies in health facilities [38,39]. Despite this situation, we detected a protective effect of becoming LTFU between 2008 and 2011 in this study. This could be explained by the support the country received through Global Fund [4] and ART sites increased from 282 sites in 2008 to 1,006 in 2012 [25].

Conclusion

The data for the 13-year window agree with the evident decline of the HIV incidence and prevalence in Zimbabwe, even from Demographic Health Survey. Our results indicate that

For peer review only - http://bmjopen.bmj.com/site/about/guidelines.xhtml

patient retention in care is still a drawback in HIV prevention and control. As much as most of the observed LTFU outcome at higher levels of care (district, provincial and central hospitals) could be "silent transfers", most patients who become LTFU are likely to have poor ART adherence if not dead. Poor ART adherence leads to poor viral suppression which has an effect towards achieving the third 90% UNAIDS targets which states that 90% of those patients on ART should be virally suppressed. Clinician should provide continual educational support on the importance to ART adherence to increase life expectancy among HIV population and other benefits of ART. Policy makers and program managers should come up with strategies which may retain patients on ART including increased patients surveillance and strengthen patients' tracing mechanisms to reduce LTFU at health facilities; strengthening community-based caregiver support [29], use of peers and nutritional aid support especially for adolescents and young (15-34years). In addition to interventions which minimise "leakages" in the HIV treatment cascade including use of ART adherence clubs strategies to reduce ART resistance [40] and sexual abstinence campaigns should remain the cornerstone of HIV prevention and control. For further studies, we recommend the use of time-varying covariates like regimen type, CD4 cell counts, WHO stages and viral load measurements; and the use of spatial analysis of ART outcome to identify high burdened regions which the policy makers may use to allocate resources, especially in resource constrained settings.

Author Contributions: **ZMZ** conceptualized the concept, cleaned and analysed the data and wrote the initial draft of the manuscript**. JT** and **TFC** advised on analysis, **EM** guided and oversaw the analysis and reviewed the manuscript. **ZMZ, JT, TC and EM** contributed to the interpretation of the results. All authors reviewed the final manuscript for submission.

Funding: This work was supported by the Developing Excellence in Leadership, Training and Science (DELTAS) Africa Initiative Sub-Saharan Africa Consortium for Advanced Biostatistics (SSACAB) [Grant No. 107754/Z/15/Z]. The DELTAS Africa Initiative is an independent funding scheme of the African Academy of Sciences (AAS) Alliance for Accelerating Excellence in Science in Africa (AESA) and is supported by the New Partnership for Africa's Development Planning and Coordinating Agency (NEPAD Agency) with funding from the Wellcome Trust [Grant No. 107754/Z/15/Z] and the United Kingdom government. The views expressed in this publication are those of the authors and not necessarily those of the AAS,

For peer review only - http://bmjopen.bmj.com/site/about/guidelines.xhtml

NEPAD Agency, Wellcome Trust, the UK government or the Ministry of Health and Child Care, Zimbabwe.

Acknowledgements: Our acknowledgements go to the Ministry of Health and Child Care, AIDS/TB Units Department for the support in data compilation and extraction for this study. We also thank the Division of Epidemiology and Biostatistics at the School of Public Health for their assistance in the getting ethical approval of this study. Thanks to Dr Nomathemba Chandiwana for technical assistance of this work.

Conflict of interest statement: All authors declare that they do not have a conflict of interest for this work.

Data sharing statement: The data used for this study can be found from a third party through an application process to the Zimbabwe Ministry of Health and Child Care through the HIV/AIDS Unit which oversees the data collection and compilation process for the ART program; therefore, the data is not publicly available.

Figure legends

Figure 1: Flowchart of the inclusion criteria for analyses of adult ART patients' enrolled in Zimbabwe ART program followed-up between 2004 and 2017

Figure 2: Schematic illustration of variable of importance selection steps and subsequent models fitted for sensitivity analysis of the results

Figure 3: Trend analysis of loss to follow-up outcome among patients receiving ART in Zimbabwe National ART Programme, 2004 to 2017

Figure 4: The cumulative incidence probability of mortality and loss to follow-up (A); and Kaplan-Meier curves for loss to follow-up stratified by baseline age categories (B), gender (C), level of urbanization of the health facilities (D), baseline tuberculosis infection status (E) and baseline World Health Organization clinical stages for ART patients from the Zimbabwe national ART Programme electronic patient monitoring data, 2004 to 2017

For peer review only - http://bmjopen.bmj.com/site/about/guidelines.xhtml

REFERENCES

[1] UNAIDS. Global AIDS UPDATE 2016 2016;17 Suppl 4:S3-11. http://www.who.int/hiv/pub/arv/global-aids-update-2016-pub/en/ [Accessed on 21 January 2018].

[2] Geng EH, Hare CB, Kahn JO, Jain V, Nunnery T Van, Christopoulos KA, et al. The Effect of a " Universal Antiretroviral Therapy " Recommendation on HIV RNA Levels Among HIV-Infected Patients Entering Care With a CD4 Count Greater Than 500 / μL in a Public Health Setting. Clinical Infectious Disease 2012;55:1690–7.

[3] Kitahata MM, Gange SJ, Abraham AG, Merriman B, Saag MS, Justice AC, et al. Effect of Early versus Deferred Antiretroviral Therapy for HIV on Survival. The New England Journal of Medicine 2009;360:1815–26.

[4] Mutasa-Apollo T, Shiraishi RW, Takarinda KC, Dzangare J, Mugurungi O, Murungu J, et al. Patient retention, clinical outcomes and attrition-associated factors of HIV-infected patients enrolled in Zimbabwe's National Antiretroviral Therapy Programme, 2007-2010. PLoS ONE 2014;9:2007–10.

[5] Rachlis B, Cole DC, van Lettow M, Escobar M, Muula AS, Ahmad F, et al. Follow-Up Visit Patterns in an Antiretroviral Therapy (ART) Programme in Zomba, Malawi. PLoS ONE 2014;9:e101875.

[6] Farahani M, Vable A, Lebelonyane R, Seipone K, Anderson M, Avalos A, et al. Outcomes of the Botswana national HIV/AIDS treatment programme from 2002 to 2010: a longitudinal analysis. The Lancet Global Health 2014;2:e44-50.

[7] Franke MF, Kaigamba F, Socci AR, Hakizamungu M, Patel A, Bagiruwigize E, et al. Improved Retention Associated With Community-Based Accompaniment for Antiretroviral Therapy Delivery in Rural Rwanda. Clinical Infectious Diseases 2013;56:1319–26. doi:10.1093/cid/cis1193.

[8] Somi G, Keogh SC, Todd J, Kilama B, Wringe A, van den Hombergh J, et al. Low mortality risk but high loss to follow-up among patients in the Tanzanian national HIV care and treatment programme. Tropical Medicine & International Health 2012;17:497–506.

[9] UNAIDS. 90-90-90 An ambitious treatment target to help end AIDS epidemic. United Nations 2014. http://www.unaids.org/sites/default/files/media_asset/90-90-90_en.pdf[Accessed 25 January 2019].

[10] Ministry of Health and Child Care NAC. Global Aids Response Progress Report 2018 Gam Zimbabwe Country Report 2017:1–24. http://www.unaids.org/sites/default/files/country/documents/ZWE_2018_countryreport.pdf [Accessed on 27 September 2018].

For peer review only - http://bmjopen.bmj.com/site/about/guidelines.xhtml

[11] Suthar AB, Rutherford GW, Horvath T, Doherty MC, Negussie EK. Improving antiretroviral therapy scale-up and effectiveness through service integration and decentralization. AIDS 2014;28:S175–85.

[12] Ministry of Health and Child Care. Guidelines for Antiretroviral Therapy for the Prevention and Treatment of HIV in Guidelines for Prevention and Treatment of HIV in Zimbabwe 2013:1–88.

[13] Ministry of Health and Child Care. Zimbabwe Electronic Patient Management System Strategic Plan Document 2012:1–52.

[14] Fine JP, Gray RJ. A Proportional Hazards Model for the Subdistribution of a Competing Risk. Journal of the American Statistical Association 1999;94:496–509.

[15] StataCorp. Stata Statistical Software: Release 15. College Station, TX: StataCorp LLC 2017.

[16] Schöni-Affolter F, Keiser O, Mwango A, Stringer J, Ledergerber B, Mulenga L, et al. Estimating loss to follow-up in HIV-infected patients on antiretroviral therapy: The effect of the competing risk of death in Zambia and Switzerland. PLoS ONE 2011;6:2–8.

[17] Assemie MA, Muchie KF, Ayele TA. Incidence and predictors of loss to follow up among HIV - infected adults at Pawi General Hospital , northwest Ethiopia : competing risk regression model. BMC Research Notes 2018;11:1–6.

[18] Tadesse K, Haile F. Predictors of Loss to Follow Up of Patients Enrolled on Antiretroviral Therapy : A Retrospective Cohort Study. AIDS & Clinical Research 2014;5:1–6.

[19] Weigel R, Estill J, Egger M, Harries AD, Makombe S, Tweya H, et al. Mortality and loss to follow-up in the first year of ART. AIDS 2012;26:365–73.

[20] Geng EH, Odeny TA, Lyamuya R, Nakiwogga-Muwanga A, Diero L, Bwana M, et al. Retention in care and patient-reported reasons for undocumented transfer or stopping care among HIV-infected patients on antiretroviral therapy in Eastern Africa: Application of a sampling-based approach. Clinical Infectious Diseases 2016;62:935–44.

[21] Molfino L, Kumar AM, Isaakidis P, Van den Bergh R, Khogali M, Hinderaker SG, et al. High attrition among HIV-infected patients with advanced disease treated in an intermediary referral center in Maputo, Mozambique. Glob Health Action 2014;7:23758.

[22] Matsena Zingoni Z, Chirwa TF, Todd J, Musenge E. HIV Disease Progression Among Antiretroviral Therapy Patients in Zimbabwe : A Multistate Markov Model. Frontiers in Public Health 2019;7:1–15. doi:10.3389/fpubh.2019.00326.

[23] Fox MP, Rosen S. Patient retention in antiretroviral therapy programs up to three years on treatment in sub-Saharan Africa, 2007-2009: Systematic review. Tropical Medicine and International Health 2010;15:1–15.

[24] Megerso A, Garoma S, Eticha TT, Workineh T, Daba S, Habtamu Z, et al. Predictors of loss to follow-up in antiretroviral treatment for adult patients in the Oromia region,

For peer review only - http://bmjopen.bmj.com/site/about/guidelines.xhtml

Ethiopia. HIV/AIDS - Research and Palliative Care 2016;8:83–92.

[25] Ministry of Health and Child Care. National HIV Care and Treatment Strategic Plan 2013-2017 and Three Year Operational Plan 2013:1–80.

[26] McHugh G, Simms V, Dauya E, Bandason T, Chonzi P, Metaxa D, et al. Clinical outcomes in children and adolescents initiating antiretroviral therapy in decentralized healthcare settings in Zimbabwe. Journal of the International AIDS Society 2017;20:1–9.

[27] Ferrand RA, Bandason T, Musvaire P, Larke N, Nathoo K, Mujuru H, et al. Causes of acute hospitalization in adolescence: Burden and spectrum of HIV-related morbidity in a country with an early-onset and severe HIV epidemic: A prospective survey. PLoS Medicine 2010;7:1–11.

[28] Maskew M, Fox MP, Evans D, Govindasamy D, Jamieson L, Malete G, et al. Insights into Adherence among a Cohort of Adolescents Aged 12-20 Years in South Africa: Reported Barriers to Antiretroviral Treatment. AIDS Research and Treatment 2016;2016:1–13. doi:http://dx.doi.org/10.1155/2016/4161738.

[29] Ferrand RA, Simms V, Dauya E, Bandason T, Mchugh G, Mujuru H, et al. The effect of community-based support for caregivers on the risk of virological failure in children and adolescents with HIV in Harare, Zimbabwe (ZENITH): an open-label, randomised controlled trial. The Lancet Child and Adolescent Health 2017;1:175–83.

[30] Takarinda KC, Sandy C, Masuka N, Hazangwe P, Choto RC, Mutasa-Apollo T, et al. Factors Associated with Mortality among Patients on TB Treatment in the Southern Region of Zimbabwe, 2013. Hindawi Tuberculosis Research and Treatment Journal 2017;2017:1–11.

[31] Alvarez-Uria G, Naik PK, Pakam R, Midde M. Factors associated with attrition, mortality, and loss to follow up after antiretroviral therapy initiation: Data from an HIV cohort study in India. Global Health Action 2013;6:1–8.

[32] Berheto TM, Haile DB, Mohammed S. Predictors of loss to follow-up in patients living with HIV/AIDS after initiation of antiretroviral therapy. North American Journal of Medical Sciences 2014;6:453–459.

[33] Andersen PK, Z SA, Susanne Rosthoj. Competing risks as a multi-state model. Statistical Methods in Medical Research 2002;11:203–15.

[34] Fatti G, Grimwood A, Bock P. Better antiretroviral therapy outcomes at primary healthcare facilities: An evaluation of three tiers of ART services in four South African provinces. PLoS ONE 2010;5:1–10.

[35] Layer EH, Kennedy CE, Beckham SW, Mbwambo JK, Likindikoki S, Davis WW, et al. Multi-level factors affecting entry into and engagement in the HIV continuum of care in Iringa, Tanzania. PLoS ONE 2014;9. doi:10.1371/journal.pone.0104961.

[36] Nyika H, Mugurungi O, Shambira G, Gombe NT, Bangure D, Mungati M, et al. Factors associated with late presentation for HIV / AIDS care in Harare City , Zimbabwe , 2015.

For peer review only - http://bmjopen.bmj.com/site/about/guidelines.xhtml

BMC Public Health 2016;16:1–7. doi:10.1186/s12889-016-3044-7.

[37] Phillips A, Vojnov L, Cohn J, Roberts T, Ellman T, Bonner K, et al. Sustainable HIV Treatment in Africa through Viral Load-Informed Differentiated Care. Nature 2016;528:S68–S76.

[38] World Health Organization. Issues and challenges - Progress on the Health-Related MDGs 2015. http://www.aho.afro.who.int/profiles_information/index.php/Zimbabwe:Issues_and_challenges_-_Progress_on_the_Health-Related_MDGs [Accessed 10 October 2018].

[39] Meldrum A. Zimbabwe's health-care system struggles on. Lancet 2008;371:1059–60.

[40] Bock P, Gunst C, Maschilla L, Holtman R, Grobbelaar N, Wademan D, et al. Retention in care and factors critical for effectively implementing antiretroviral adherence clubs in a rural district in South Africa. Journal of the International AIDS Society 2019;22:5–9.

For peer review only

For peer review only - http://bmjopen.bmj.com/site/about/guidelines.xhtml

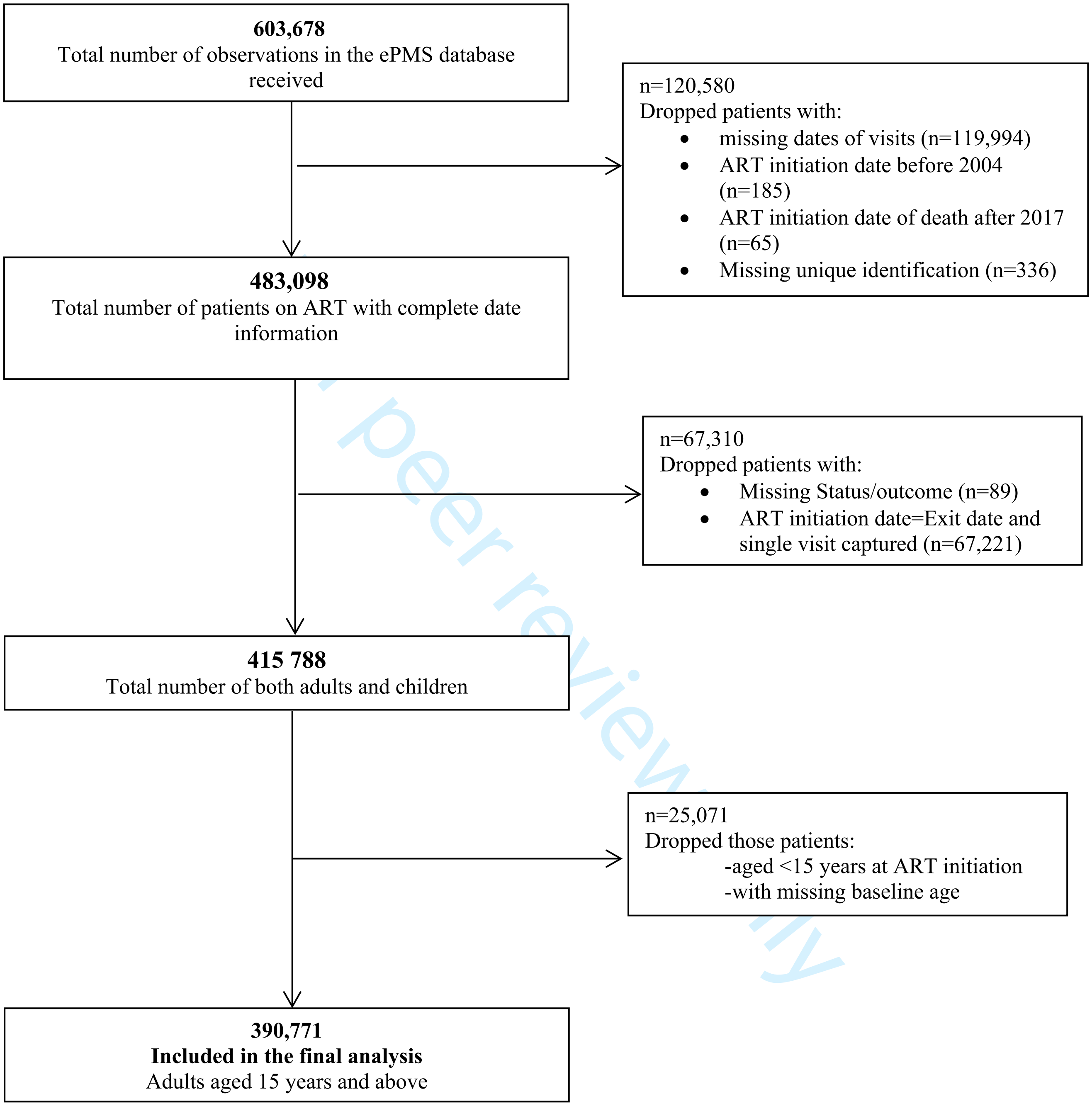

Figure 1: Flowchart of the inclusion criteria for analyses of adult ART patients' enrolled in Zimbabwe ART program followed-up between 2004 and 2017

For peer review only - http://bmjopen.bmj.com/site/about/guidelines.xhtml

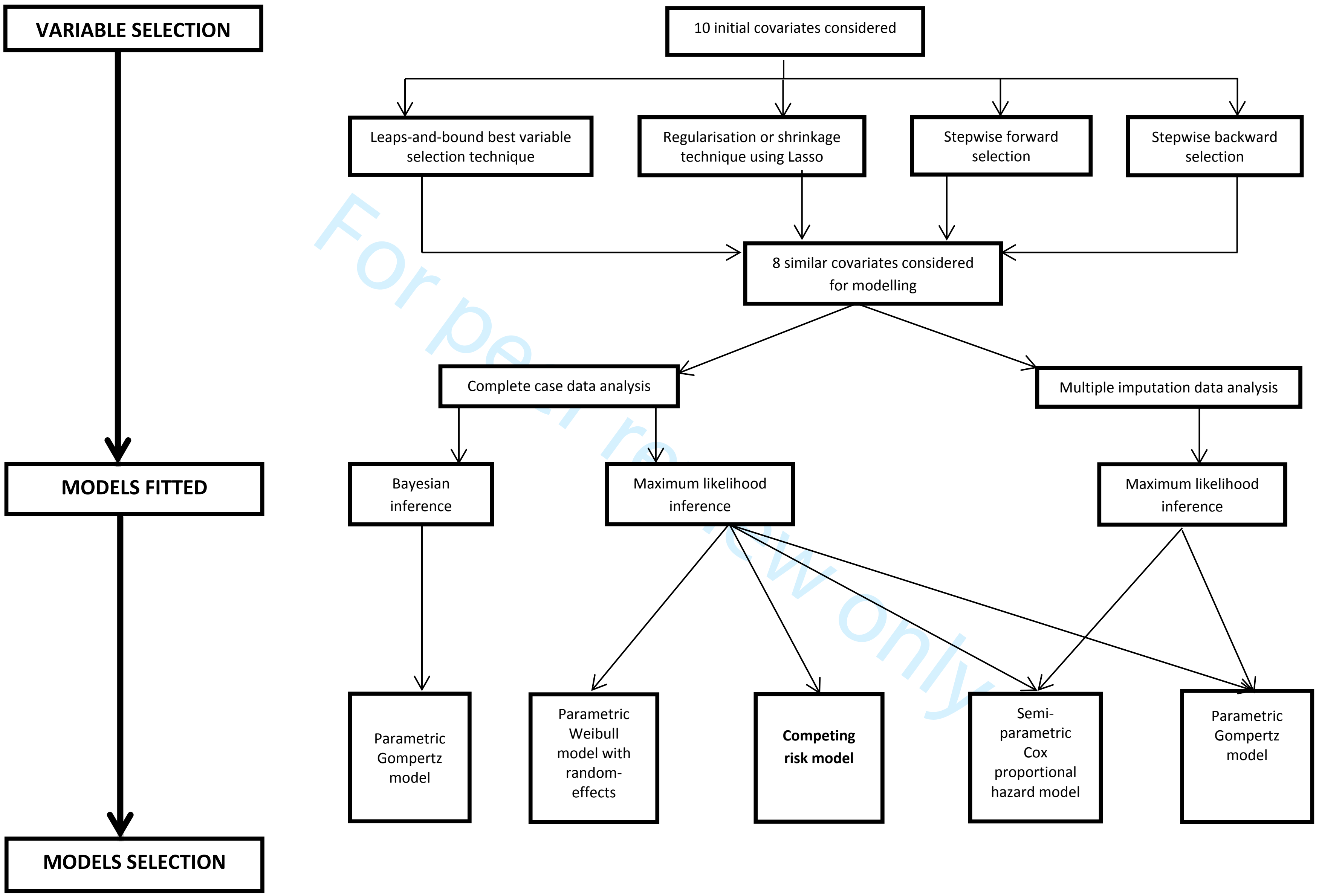

Figure 2: Schematic illustration of variable of importance selection steps and subsequent models fitted for sensitivity analysis of the results

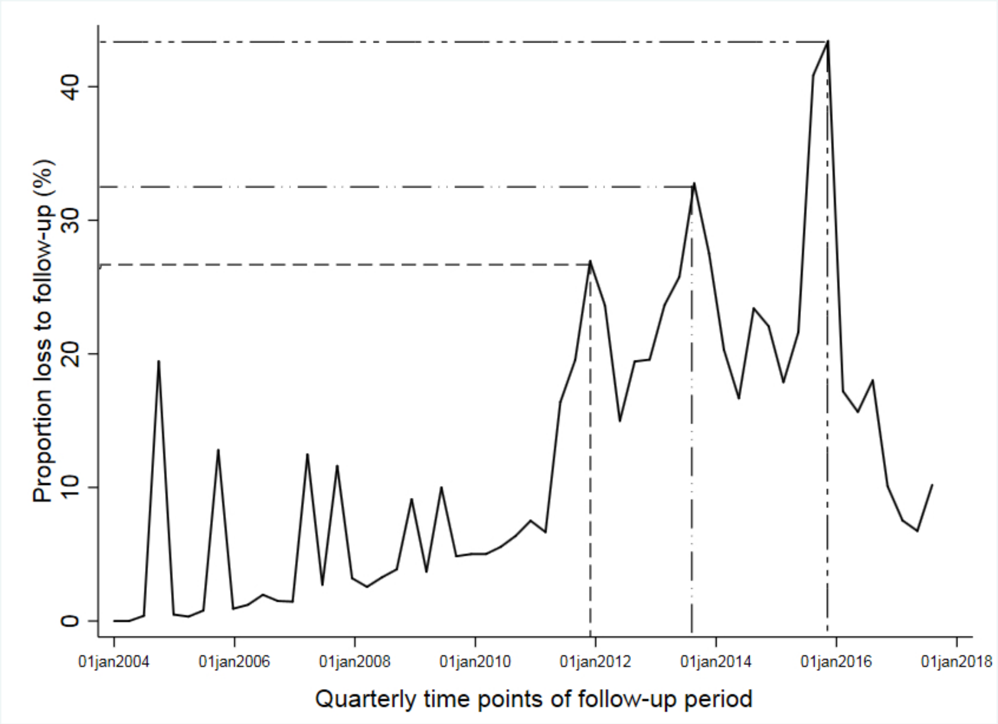

Figure 3: Trend analysis of loss to follow-up outcome among patients receiving ART in Zimbabwe National ART Programme, 2004 to 2017

298x217mm (72 x 72 DPI)

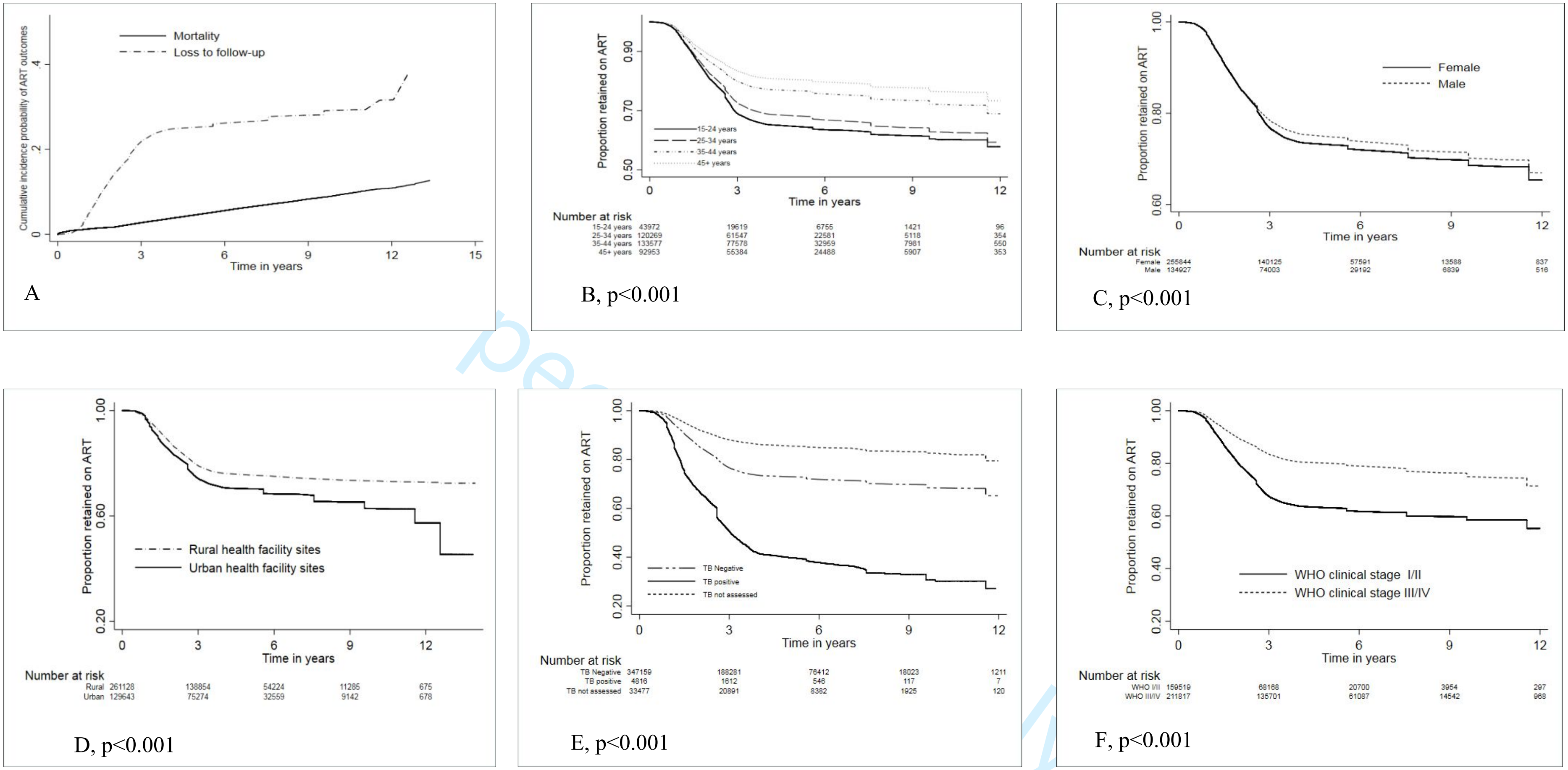

Figure 4: The cumulative incidence probability of mortality and loss to follow-up (A); and Kaplan-Meier curves for loss to follow-up stratified by baseline age categories (B), gender (C), level of urbanization of the health facilities (D), baseline tuberculosis infection status (E) and baseline World Health Organization clinical stages for ART patients from the Zimbabwe national ART Programme electronic patient monitoring data, 2004 to 2017

APPENDICES

A1; Cox proportional hazard

Traditionally for time-to-event analysis, researchers tend to fit the semi-parametric cause-specific proportional hazard model which was proposed by Cox (1972) because of its less stringent assumption on the hazard form and survival function (62). For the conventional cause-specific proportion hazard, we did a non-informative censoring of LTFU and transfer-out. We assumed that the hazard function is arbitrary, unspecified, non-negative function of time (62).

A2: Survival Function

Let *T* be a non-negative random variable denoting the survival time of an individual. The survival function [*S(t)*] is used to represent the probability that an individual survives from time origin to sometime beyond *t*

$$S(t) = P(T \geq t) \tag{1}$$

If we assume that *T* is a continuous random variable $[P(T \geq t) = P(T > t)]$, the different values that *T* can take have an underlying probability density function (pdf) given by:

$$S(t) = P(T > t) = \int_0^t f(x)dx = 1 - F(t) \tag{2}$$

Where *F(t)* is the cumulative distribution function. The cumulative distribution function can be expressed as:

$$F(t) = P(T < t) = \int_0^t f(x)dx \tag{3}$$

Given a survival function, we can calculate the density function by differentiating the cumulative distribution function as:

$$f(t) = -\frac{dF(t)}{dt} \tag{4}$$

A3: The Hazard function

The hazard function is the probability that an individual dies at time t conditional on having survived to that time. The hazard function therefore represents an instantaneous death (failure) rate for an individual who survived to that time. In other words, the hazard function consider the probability that a random variable is associated with an individual's survival time lies between *t* and *t*+ δt conditional on *T* being greater than or equal to *t*:

$$\begin{aligned} h(t) &= P(t < T < t + \delta t \mid T \geq t) \\ &= \frac{F(t+\delta t) - F(t)}{S(t)} \end{aligned} \tag{5}$$

where *F(t)* is the cumulative distribution function. The hazard function is the limiting value of the above probability in Equation 1.5 divided by the time interval δt, as δt tends to zero. This can be re-written as:

$$
\begin{aligned}
h(t) = \mu(t) &= \lim_{\delta t \to 0} \frac{P[t \le T < t + \delta t \mid T \ge t]}{\delta t} \\
&= \lim_{\delta t \to 0} \frac{P[t \le T < t + \delta t]}{\delta t P(T > t)} \\
&= \lim_{\delta t \to 0} \frac{F[t + \delta t - F(t)]}{\delta t S(t)} \\
&= \frac{1}{S(t)} \left\{ \lim_{\delta t \to 0} \frac{F[t + \delta t - F(t)]}{\delta t} \right\}
\end{aligned} \tag{6}
$$

But $\left\{ \lim_{\delta t \to 0} \frac{F[t + \delta t - F(t)]}{\delta t} \right\} = f(t)$ and since T is a continuous random variable, then

$$
h(t) = \frac{f(t)}{S(t)} = -\frac{d}{dt} \log[S(t)] \tag{7}
$$

The cumulative hazard is

$$
H(t) = \int_0^t h(u)du = -\log[S(x)] \tag{8}
$$

Thus for a continuous lifetimes

$$
\begin{aligned}
S(t) &= \exp\{-H(t)\} \\
&= \exp\left[-\int_0^t h(u)du \right]
\end{aligned} \tag{9}
$$

A4: The Cox regression model

This model is also known as the Proportional hazard regression model, which models the incidence or hazard rate (mortality rate in our case). This model assumes independence of survival times between distinct individuals in a sample and a constant hazard ratio regardless of time. The model can be written as:

$$
\begin{aligned}
\log\left(\frac{h(t)}{h_0(t)} \right) &= \beta_1 X_1 + \beta_2 X_2 + \ldots + \beta_k X_k \\
\left(\frac{h(t)}{h_0(t)} \right) &= \exp(\beta_1 X_1 + \beta_2 X_2 + \ldots + \beta_k X_k) \\
h(t) &= h_0(t) \exp(\beta_1 X_1 + \beta_2 X_2 + \ldots + \beta_k X_k)
\end{aligned} \tag{10}
$$

This model can be simplified in a matric form as:

$$
h(t / \mathbf{Z}) = h_0(t) \exp(\boldsymbol{\beta}\ \mathbf{Z}^T)
$$

where $h(t/\mathbf{Z})$ is the expected hazard at time *t* conditional to the covariate matrix $\mathbf{Z}$. The expected hazard is a product of the baseline hazard ($h_0(t)$) (which is a function of duration time only) and the exponential function of the regressors ($\boldsymbol{\beta} = (\beta_1, \beta_2, ..., \beta_k)$; a 1 x *k* parameter vector) for k covariates. Then the explanatory variables for n persons, $\mathbf{Z}$ matrix, will be:

$$\mathbf{Z} = \begin{bmatrix} X_{11}, & X_{12}, & . & . & ., & X_{1n} \\ X_{21}, & X_{22}, & . & . & ., & X_{2n} \\ . & . & . & . & . & . \\ . & . & . & . & . & . \\ . & . & . & . & . & . \\ X_{k1}, & X_{k2}, & . & . & ., & X_{kn} \end{bmatrix}$$

Thus for the individuals covariates $Z_i = (X_{i1},...., X_{ik})^T$, the baseline hazard ($h_0(t)$ is equal to *h(t)* when all the regressors are equal to zero, the function of the regressors will be equal to 1. The Cox model is termed semi-parametric because it is made up of the parametric part for the covariates adjustment and the non-parametric baseline hazard as there are no assumptions for the underlying hazard function.

A5: Likelihood function of the Cox regression

The Cox model parameters are estimated by maximizing the partial log-likelihood function. Letting the minimum censoring time Cj and survival time T_j be denoted by $t_{(j)}$. The censoring time is right censored at C after observing that T_j > Cj. Let $R(t_j)$ denotes the set of lives which are at risk before the *j*th observed lifetime. Then the partial likelihood is a product of the event times of a quotient that compares the hazard of individuals with the event at time $t_{(j)}$ to all the hazard of all individuals at risk at time $t_{(j)}$ can be derived in terms of these censoring times. If we observe $\left\{\left(T_j, \delta_j\right)\right\}_{j=1}^{n}$ where T_j is still the observed time and δ_j the censoring indicator taking values 1 if the failure time is observed for individuals $j = 1, 2, ..., n$ where n is the total number of observations and 0 if right censoring, then the likelihood can be easily expressed as a product of the probability density function (pdf) and the cumulative survival function expressed as $LH \propto \prod_{j=1}^{n} f\left(t_j\right)^{\delta_j} S\left(t_j +\right)^{1-\delta_j}$. This function can be expressed as well as a partial likelihood function in terms of covariates and regression coefficients matrices as shown below:

For peer review only

$$L_p(\beta \mid D_{obs}) = \prod_{j=1}^{n} \left[\frac{\exp(\boldsymbol{Z}_j^T \boldsymbol{\beta})}{\sum_{k \in R(t_j)} \exp(\boldsymbol{Z}_k^T \boldsymbol{\beta})} \right]^{\delta_j}, \quad (11)$$

where $D_{obs} = \{(t_j, \delta_j, \boldsymbol{Z}) : j = 1, 2, ..., n\}$ is the observed univariate right censored survival data. Considering the Breslow concept when ties are present, the partial likelihood may be expressed as:

$$L_{pt}(\beta \mid D_{obs}) = \prod_{j=1}^{d} \frac{\exp(\boldsymbol{Z}_j^T \boldsymbol{\beta})}{\left[\sum_{k \in R(t_j)} \exp(\boldsymbol{Z}_k^T \boldsymbol{\beta}) \right]^{d_j}},$$

where $d = \sum_{j=i}^{n} \delta_j, z_j = \sum_{k \in D_j} \boldsymbol{x}_j, d_j =$ the number of events at $t_{(j)}$ and $R(t_j)$ is a set of all individuals who have the event at the defined time $t_{(j)}$. Thus, this can be written as

$$L_{pt}(\beta \mid D_{obs}) = \prod_{j=1}^{n} \frac{\exp(\delta_j \boldsymbol{Z}_j^T \boldsymbol{\beta})}{\left[\sum_{k \in R(t_j)} \exp(\boldsymbol{Z}_k^T \boldsymbol{\beta}) \right]^{\delta_j}},$$

Expanding the Breslow equation gives:

$$\log L(\beta \mid D_{obs}) = \sum_{j=1}^{D} \left[\sum_{i \in D_j}^{n} \boldsymbol{Z}_j^T \boldsymbol{\beta} - d_j \log \left\{ \sum_{k \in R_j}^{n} \exp(\boldsymbol{Z}_j^T \boldsymbol{\beta}) \right\} \right] \quad (12)$$

where *j* indexes the ordered failure times $t_{(j)}, j = 1, ..., D$; D_j is the set of d_j observations that fail at time $t_{(j)}$; d_j is the number of failures at time $t_{(j)}$; and R_j is the set of observations *k* that are at risk at time $t_{(j)}$ (that is , all k such that $t_{0k} < t_{(j)} \le t_k$). This can be simplified in set natation form as $k \in R_j$ and $d_j \in D_j$.

A6: Competing risk model specification (29)

In epidemiological studies, more than one cause of failure is possible, hence the estimates of the targeted outcome probabilities are either over-estimated with the Cox model (63) hence the drive for the competing risk analysis to get marginal probabilities of mortality as proposed by Fine and Gray (27) . Competing risk is when we have more than one cause of failure outcomes and we are only interested in one of them. In this case mortality was the competing event to LTFU. The Kaplan-Meier estimate is normally used, where the competing risk events are treated as censored observations but this is biased since the independent assumption is violated. An individual who is censored because of the competing event will certainly not experience the outcome of interest hence the Kaplan-

For peer review only

Meier may overestimate the probability of failure especially if completion is high. Hence, using the Kaplan-Meier estimate in this situation would result in the biased estimates.

The competing risk inference is the joint distribution of time of death or failure (T) and failure outcome, in our case death (D) conditional to some covariates, X. The cause-specific hazard (q cause of death) function will be

$$h_q(t) = \lim_{\delta x \to 0} \frac{P(t \le T < t + \delta t, D = q \mid T \ge t)}{\delta t} \tag{13}$$

The cumulative cause-specific hazard is written as:

$$H_q(t) = \int_0^t h_q(u) du \tag{14}$$

And define

$$S_q(t) = \exp(H_q(t)) \tag{15}$$

The cumulative incidence function or the sub distribution function for death is defined as:

$$I_q(t) = P(T \le t, D = q) = \int_0^t h_q(u) S(u) du \tag{16}$$

This function is sub-distribution since the cumulative probability of death is always below one, that is, $I_q(\infty) = P(D = q) < 1$

The model specification would be:

$$h_q(t) = \frac{d \log(1 - I_q(t))}{dt}$$

is considered the sub-distribution hazard and the model is expressed as

$$h_q(t / \mathbf{Z}) = h_{q,0}(t) \exp(\boldsymbol{\beta}_q \mathbf{Z}^T) \tag{17}$$

where $h_{q,0}$ is the baseline sub-distribution hazard of cause q and the vector $\boldsymbol{\beta}_q$ represents the covariates effects of cause q . To estimate the unknown $\boldsymbol{\beta}_q$ parameters, we maximize the following log-pseudolikelihood function:

$$\log L(\beta \mid D_{obs}) = \sum_{i=1}^{n} \delta_i w_i \left[\boldsymbol{Z}_i^T \boldsymbol{\beta} + offset_i - \log \left\{ \sum_{k \in R_i}^{n} w_i \alpha_{ik} \exp(\boldsymbol{Z}_k^T \boldsymbol{\beta} + offset_k) \right\} \right] \tag{18}$$

where δ_i is death for an ith individual, R_i are the set of observations, q at risk at time t_i . The only difference with the Cox is the α_{iq} weights. These weights keep individuals who experience the competing event to be in subsequent risk sets and to reduce their weight over time hence their likelihood of being censored increases.

For peer review only

STROBE 2007 (v4) Statement—Checklist of items that should be included in reports of *cohort studies*

Section/Topic	Item #	Recommendation	Reported on page #
**Title and abstract**	1	(*a*) Indicate the study's design with a commonly used term in the title or the abstract	1
		(*b*) Provide in the abstract an informative and balanced summary of what was done and what was found	1-2
**Introduction**			
Background/rationale	2	Explain the scientific background and rationale for the investigation being reported	3
Objectives	3	State specific objectives, including any prespecified hypotheses	4
**Methods**			
Study design	4	Present key elements of study design early in the paper	4
Setting	5	Describe the setting, locations, and relevant dates, including periods of recruitment, exposure, follow-up, and data collection	4-5
Participants	6	(*a*) Give the eligibility criteria, and the sources and methods of selection of participants. Describe methods of follow-up	4
		(*b*) For matched studies, give matching criteria and number of exposed and unexposed	n/a
Variables	7	Clearly define all outcomes, exposures, predictors, potential confounders, and effect modifiers. Give diagnostic criteria, if applicable	5
Data sources/ measurement	8*	For each variable of interest, give sources of data and details of methods of assessment (measurement). Describe comparability of assessment methods if there is more than one group	
Bias	9	Describe any efforts to address potential sources of bias	5
Study size	10	Explain how the study size was arrived at	4
Quantitative variables	11	Explain how quantitative variables were handled in the analyses. If applicable, describe which groupings were chosen and why	4
Statistical methods	12	(*a*) Describe all statistical methods, including those used to control for confounding	5
		(*b*) Describe any methods used to examine subgroups and interactions	n/a
		(*c*) Explain how missing data were addressed	6
		(*d*) If applicable, explain how loss to follow-up was addressed	n/a
		(*e*) Describe any sensitivity analyses	6
**Results**			

For peer review only

Participants	13*	(a) Report numbers of individuals at each stage of study—eg numbers potentially eligible, examined for eligibility, confirmed eligible, included in the study, completing follow-up, and analysed	4
		(b) Give reasons for non-participation at each stage	n/a
		(c) Consider use of a flow diagram	4
Descriptive data	14*	(a) Give characteristics of study participants (eg demographic, clinical, social) and information on exposures and potential confounders	7-8
		(b) Indicate number of participants with missing data for each variable of interest	8
		(c) Summarise follow-up time (eg, average and total amount)	7
Outcome data	15*	Report numbers of outcome events or summary measures over time	7
Main results	16	(*a*) Give unadjusted estimates and, if applicable, confounder-adjusted estimates and their precision (eg, 95% confidence interval). Make clear which confounders were adjusted for and why they were included	10
		(*b*) Report category boundaries when continuous variables were categorized	n/a
		(*c*) If relevant, consider translating estimates of relative risk into absolute risk for a meaningful time period	n/a
Other analyses	17	Report other analyses done—eg analyses of subgroups and interactions, and sensitivity analyses	10
**Discussion**			
Key results	18	Summarise key results with reference to study objectives	12
**Limitations**			
Interpretation	20	Give a cautious overall interpretation of results considering objectives, limitations, multiplicity of analyses, results from similar studies, and other relevant evidence	12-15
Generalisability	21	Discuss the generalisability (external validity) of the study results	15
**Other information**			
Funding	22	Give the source of funding and the role of the funders for the present study and, if applicable, for the original study on which the present article is based	15

*Give information separately for cases and controls in case-control studies and, if applicable, for exposed and unexposed groups in cohort and cross-sectional studies.

Note: An Explanation and Elaboration article discusses each checklist item and gives methodological background and published examples of transparent reporting. The STROBE checklist is best used in conjunction with this article (freely available on the Web sites of PLoS Medicine at http://www.plosmedicine.org/, Annals of Internal Medicine at http://www.annals.org/, and Epidemiology at http://www.epidem.com/). Information on the STROBE Initiative is available at www.strobe-statement.org.

For peer review only

BMJ Open

Competing risk of mortality on loss to follow-up outcome among HIV patients on ART: A retrospective cohort study from the Zimbabwe National ART Programme

Journal:	*BMJ Open*
Manuscript ID	bmjopen-2019-036136.R1
Article Type:	Original research
Date Submitted by the Author:	13-Jun-2020
Complete List of Authors:	Matsena Zingoni, Zvifadzo; University of the Witwatersrand Faculty of Health Sciences, Division of Epidemiology and Biostatistcs; Ministry of Health and Child Care, National Institute of Health Research<br>Chirwa, Tobias; University of the Witwatersrand Faculty of Health Sciences, Division of Epidemiology and Biostatistics<br>Todd, Jim; LSHTM, EPH<br>Musenge, Eustasius; University of the Witwatersrand Faculty of Health Sciences, Division of Epidemiology and Biostatistics
**Primary Subject Heading**:	HIV/AIDS
Secondary Subject Heading:	Public health, Epidemiology, Health services research, Infectious diseases, Health policy
Keywords:	Epidemiology < INFECTIOUS DISEASES, HIV & AIDS < INFECTIOUS DISEASES, Public health < INFECTIOUS DISEASES

SCHOLARONE™
Manuscripts

I, the Submitting Author has the right to grant and does grant on behalf of all authors of the Work (as defined in the below author licence), an exclusive licence and/or a non-exclusive licence for contributions from authors who are: i) UK Crown employees; ii) where BMJ has agreed a CC-BY licence shall apply, and/or iii) in accordance with the terms applicable for US Federal Government officers or employees acting as part of their official duties; on a worldwide, perpetual, irrevocable, royalty-free basis to BMJ Publishing Group Ltd ("BMJ") its licensees and where the relevant Journal is co-owned by BMJ to the co-owners of the Journal, to publish the Work in this journal and any other BMJ products and to exploit all rights, as set out in our licence.

The Submitting Author accepts and understands that any supply made under these terms is made by BMJ to the Submitting Author unless you are acting as an employee on behalf of your employer or a postgraduate student of an affiliated institution which is paying any applicable article publishing charge ("APC") for Open Access articles. Where the Submitting Author wishes to make the Work available on an Open Access basis (and intends to pay the relevant APC), the terms of reuse of such Open Access shall be governed by a Creative Commons licence – details of these licences and which Creative Commons licence will apply to this Work are set out in our licence referred to above.

Other than as permitted in any relevant BMJ Author's Self Archiving Policies, I confirm this Work has not been accepted for publication elsewhere, is not being considered for publication elsewhere and does not duplicate material already published. I confirm all authors consent to publication of this Work and authorise the granting of this licence.

For peer review only

Competing risk of mortality on loss to follow-up outcome among HIV Patients on ART: A retrospective cohort study from the Zimbabwe National ART Programme

Zvifadzo Matsena Zingoni[§, 1, 2], Tobias Freeman Chirwa[1], Jim Todd[3] and Eustasius Musenge[1]

[1]Division of Epidemiology and Biostatistics, University of the Witwatersrand, Johannesburg, South Africa

[2]National Institute of Health Research, Ministry of Health and Child Care, Harare, Zimbabwe

[3]Department of Population Health, London School of Hygiene and Tropical Medicine, London, United Kingdom

[§]Corresponding author: Zvifadzo Matsena Zingoni
27 St Andrews' Road
Parktown, 2193 Johannesburg, South Africa
email: zmatsena28@gmail.com
ORCID number: 0000-0002-7993-1187

Authors email addresses of authors:
Tobias Freeman Chirwa (TFC): Tobias.Chirwa@wits.ac.za
Jim Todd (JT): jim.todd@lshtm.ac.uk
Eustasius Musenge (EM): Eustasius.Musenge@wits.ac.za

For peer review only

Word count

Abstract: 299
Main body: 3975

ABSTRACT

Objective: To determine the loss to follow-up (LTFU) rates at different health care levels after ART services decentralisation among ART patients who initiated ART between 2004 and 2017 using the competing risk model in addition to the Kaplan-Meier and Cox regressions analysis.

Design: A retrospective cohort study

Setting: The study was done in Zimbabwe using a nationwide routinely collected HIV patient-level data from various health levels of care facilities compiled through the electronic patient management system (ePMS).

Participants: We analyse 390,771 participants aged 15 years and above from 538 health facilities.

For peer review only - http://bmjopen.bmj.com/site/about/guidelines.xhtml

Outcomes: The primary endpoint was LTFU defined as a failure of a patient to report for drug refill for at least 90 days from last appointment date or if the patient missed the next scheduled visit date and never showed up again. Mortality was considered a secondary outcome if a patient was reported to have died.

Results: The total exposure time contributed was 1,544,468 person-years. LTFU rate was 5.75(95% confidence interval (CI): 5.71-5.78) per 100 person-years. Adjustment for the competing event independently increased LTFU rate ratio in provincial and referral (adjusted sub-hazard ratios (AsHR) 1.24; 95%CI: 1.20-1.28) and district and mission (AsHR 1.47; 95%CI: 1.45-1.45) hospitals (reference: primary health care); in urban sites (AsHR 1.58; 95%CI: 1.55-1.60) (reference: rural); and among adolescence and young adults (15-24 years) group (AsHR 1.17; 95%CI: 1.15-1.19) (reference: 35-44 years). We also detected overwhelming association between LTFU and tuberculosis infected patients (AsHR 1.55; 95%CI: 1.47-1.64) (reference: no tuberculosis).

Conclusions: We have observed considerable findings that "leakages" within the ART treatment cascade persist even after the decentralisation of health services. Risk factors for LTFU reflect those found in sub-Saharan Africa studies. Interventions which retain patients in care by minimising any "leakages" along the treatment cascade are essential in attaining the UNAIDS 90-90-90 fast track targets.

Keywords: Antiretroviral therapy, Competing risk analysis, Loss to follow-up, Mortality, Retention, Risk factors,

Article Summary: Strength and limitations of the study

- The analysis is based on a huge national routinely collected individual-level dataset which has a big follow-up period making our findings generalisable to the HIV patients on ART in Zimbabwe.
- This study fitted a competing risk model based on the cumulative incidence function (CIF) to explore the association between selected covariates and the absolute risk which is an essential modelling approach for medical decision-making so that appropriate sub-population intervention can be implemented.

For peer review only

For peer review only - http://bmjopen.bmj.com/site/about/guidelines.xhtml

- Variable selection of independent factors was performed using the regularisation (shrinkage) lasso regression technique alongside the stepwise section methods.
- Important factors known to influence LTFU like CD4 cell counts and viral load were not accounted for in the model due to the high percentage of missingness, and the independent factors adjusted for were not time-dependent.
- This study has a possibility of participant selection bias since only participants enrolled in health facilities linked to the electronic patients' management database were conveniently sampled for analysis.

INTRODUCTION

Globally, remarkable progress has been made on the treatment of HIV-infected people using antiretroviral therapy(ART), particularly in sub-Saharan Africa(SSA)[1]. In Zimbabwe, the ART programme is a comprehensive care and support package aiming to slow disease progression[2], improve survival and quality of life [3]; and minimise HIV transmission by reducing the viral load. Therefore, it is imperative to monitor individuals on ART to ensure treatment efficacy, support adherence; and identify treatment failure and resistance.

At the national level, aggregated health facility data is usually used for monitoring ART programme; however, rigorous analyses are restricted. This promoted the steady evolving of patient-level data worldwide[4–8]. With efforts to achieve zero HIV incidences by 2030, the 90-90-90 UNAIDS targets were launched, whereby 90% of the people living with HIV (PLWHIV) should know their status, 90% of those diagnosed should be initiated on ART, and 90% of those on ART should achieve viral suppression[9]. In Zimbabwe, strides have been made towards achieving these targets; however, limitations exist. For instance, only 73% of PLWHIV in Zimbabwe who are on ART have achieved viral suppression [10]. To improve the trailing last 90% indicator, it is crucial to come up with broad efforts which minimise long-term ART attrition due to loss to follow-up (LTFU), mortality, drop-outs and withdrawals.

In Zimbabwe, the health service delivery can be categorised into four levels of care, namely the primary, secondary, tertiary and quaternary[11]. The primary care consists of clinics and facilities offering basic preventive and curative services. Majority of these primary health care facilities are in the rural areas, and health problems beyond the scope of these facilities are

For peer review only - http://bmjopen.bmj.com/site/about/guidelines.xhtml

referred to the secondary health care centres, which are mainly the district and mission hospitals. In the secondary care category, private and company health facilities may fit in if they can handle emergency services as the district hospitals. The tertiary care consists of the provincial hospitals which received referral patients from the district hospitals[12]. The quaternary care is the last referral care level which received patients from provincial hospitals. These facilities are mainly in the two largest cities in Zimbabwe[12]. The quaternary health care offers much more advanced health care; hence, there is modern equipment, medical specialists and pharmaceutical, which could be missing in the lower levels of care[11].

The ART services were initially centralised at the higher levels of care; however, with the aim to increased treatment coverage and easy access to those in need, the ART services were decentralised gradually around 2010/11 [13]. The differentiated care models were implemented in Zimbabwe to improve the retention in care of PLWHIV, and the impact of ART decentralisation has been well documented in the SSA region[14]. Understanding ART retention patterns after the implementation of differential models is vital to guide the ART programme and policy as well as reflecting on some of the gaps which may contribute to the tailing third 90% target. One of the main threats of patients' retention in care is LTFU outcome which is more likely to persist even after the decentralisation of ART care.

Therefore, this study aims to determine the loss to LTFU rates at different health care levels after ART services decentralisation among ART patients who initiated ART between 2004 and 2017. The LTFU rates were compared between the different health care facility types (primary health care (PHC), district/mission and provincial/referral hospitals) in Zimbabwe using routinely collected patients-level data from the Zimbabwe National ART database. We further adjusted for the individual-level demographic and clinical characteristics as predisposing or independent factors for LTFU in the multiple adjusted competing risk model of mortality on the LTFU outcome.

METHODS

Study Population

This study was conducted in Zimbabwe, a landlocked country in SSA with roughly 1.4 million PLWHIV and HIV prevalence of 13.7% among adults in 2017[10]. Individual patients aged 15

For peer review only - http://bmjopen.bmj.com/site/about/guidelines.xhtml

years and above, who initiated ART between 1 January 2004 and 31 December 2017, were eligible for this analysis. A full description of how the final sample size was achieved is illustrated in Figure 1.

[Insert Figure 1]

Study design and Data sources

This study was a secondary data analysis from a retrospective cohort study of routinely collected patient data from the Zimbabwe nation ART programme. The data came from the national ART programme, which started in 2004 with ART sites expanded to 1,566 by 2017[10]. Treatment guidelines followed WHO recommendations; hence, the eligibility criteria and first-line regimens changed over time. The initial regimen for an ART naïve patient is the first-line ART, a combination of two nucleoside reverse transcriptase inhibitors(NRTI), the first drug which can be Zidovudine(AZT), Tenofovir(TDF) (the most common) or Stavudine(D4T) and the second drug is mainly Lamivudine (3TC); and one non-NRTI which can either be Nevirapine(NVP) or Efavirenz(EFV). In this study, majority of the participants were receiving three-drug combination therapy of TDF+3TC+EFV therapy as the first-line treatment. Once the patient experienced treatment failure or intolerance, switch to second-line ART (a three-drug combination of protease inhibitor and two NRTIs) can be made[15].

Monitoring and Evaluation System

The monitoring and evaluation system of the ART programme in Zimbabwe has been described elsewhere[16]. Briefly, since the inception of ART, all patient monitoring records (PMR) have been collected manually using a paper-based system, kept at the health facility where the patient is registered. However, by 2008, the paper-based system was no longer functioning properly as a result of high volumes of patients who require ART which was estimated at 1,119,909 patients by the end of 2017 [10]. This upsurge in patients' volumes began to affect the accurate monitoring and reporting of patients on ART as the paper-based system could not cope. Moreover, the paper-based system translated into huge workloads and eventually became strenuous to the already over-burdened health workers. All this gave birth to the inception of the through the electronic patient management system (ePMS) in 2012 with the ultimate goal of phasing out the paper-based system nationwide.

For peer review only - http://bmjopen.bmj.com/site/about/guidelines.xhtml

The ePMS primary aim was to increase efficiency and effective management of ART patients; helps reduce the lack of follow-up of patients on ART through providing an easier tracking system which subsequently increases the likelihood of ART adherence [16]. The implementation of this electronic system meant that improved data quality of patients' history information becomes available since clinicians require an evidence-based approach to make a well-informed clinical decision[10]. The ePMS led to automated patient care and treatment operations and improved emergency response [10]. The electronic system provided a platform to compile quality patient-level data to accurately make forecast and evaluate the ART intervention, enabling a more effective program.

The ePMS was rolled out in systematic phased approach with priority given to those HIV sites with high volumes of HIV patients and also maintaining a good representation of the four health care levels in the country [16]. From the ePMS Strategic Plan document, the first phase target was 83 sites in 2013 comprising of central, provincial and district hospitals which covered approximately 61% of patients on ART nationwide while in 2014, addition 267 city polyclinics, rural/large mission hospitals sites were included to make a total of 350 sites by the end of that same year [16]. The last phase included 184 static ART follow up sites to bring the total to 534 sites by the end of 2017. With these sites covered, approximately 97% of all patients receiving ART were considered to be covered. By the end of 2018, there were 622 sites with a functional ePMS with the inclusion of private hospitals [10].

The final database of the ePMS has patients' information from all levels of care (quaternary, tertiary, secondary and primary health care levels). It, therefore, reflects the overall ART management services in the country. At enrolment, the patient's demographic data are captured. At each subsequent clinic visit, information on ART regimen type, clinical information, laboratory investigations and physical examination is captured. Outcomes are assessed and captured as death, LTFU, transfer-out to another health facility or alive and active on treatment. Already existing patient's information from the paper-based system was retrospectively transcribed into the electronic database at each clinic, and all subsequent clinic visits were recorded prospectively with a real-time entry of patient records.

The introduction of the ePMS for monitoring HIV patients in 2012 provided a platform for huge data repository in the country which may be used to answer some of the public health questions

For peer review only - http://bmjopen.bmj.com/site/about/guidelines.xhtml

on the HIV aspects using statistical models. Moreover, the ePMS platform provides readily available data archiving for robust statistical analysis approaches to guide evidence-based decision-making and programme interventions planning at patients-level while eliminating the aggregated data. Therefore, this rich, huge patient-level database was of preference and suitable to answer our study objective.

Study Endpoints

The primary endpoint was LTFU defined as a failure of a patient to report for drug replenishment for at least 90 days from last appointment date or if the patient missed the next scheduled visit date and never showed up again. Patients, who returned after having been LTFU, were classified as alive and active on treatment. We also consider the mortality outcome as a competing event of LTFU if a patient was reported to have died.

Data Analysis Methods

Descriptive summaries were reported as frequencies and proportions stratified by health facility type. Calendar trend pattern of LTFU was explored and incidence rates calculated from the ART initiation date to outcome occurrence date on a quarterly time scale. Kaplan-Meier non-parametric curves (Equations 1-4 in Appendix) were used to describe the probability of LTFU over time stratified by selected covariates. The log-rank test was used to assess the equality of these patient retention probabilities. Estimated hazard rates (HR) (Equation 5-9 in Appendix) were reported as per 100 person-years.

Important variable selection was done using the lasso regression regularisation technique. Variable selection sensitivity analysis was done using the subset selection techniques (best subset selection, stepwise forward variable selection and stepwise backward variable selection methods). All these methods yield the same independent factors considered in the seven different multiple adjusted time-to-event models fitted, Figure 2. The predictors adjusted for were all categorical, namely: health facility type, health facility site, age groups, tuberculosis status, body functional status, WHO clinical stage, calendar period and regimen type. The stratification of the health facility type into primary health care, district/mission hospitals, and provincial/referral hospitals was based on the level of care classification of the corresponding health facility an individual was enrolled in. For those individuals who had moved from one health facility to the

For peer review only - http://bmjopen.bmj.com/site/about/guidelines.xhtml

other during the study period covered, the last recorded health facility site was considered for this analysis. Therefore, the health level of care classification was mutually exclusive as one individual was assigned to the last captured health facility.

[Insert Figure 2]

The standard Cox regression model defined in Equation 10-12 in the Appendix is normally used for a time to event analysis; however, this method may give biased estimates in the presence of competing events. We fitted the competing risk (CR) model of mortality on LTFU through the cumulative incidence function (CIF) define in Equation 13-18 in Appendix) [17]. CR analysis assumes that patients are exposed to at least two risks (mortality and LTFU). One of the events is of interest (LTFU) while the other is a competing event (mortality) which inhibits the outcome of interest from occurring. The competing events may not be independent, and the baseline hazard may differ between them. The covariates used were time-independent; however, we adjusted for time to take into account the period the country experienced an economic turmoil around 2008 which resulted in a mass withdrawal of health personnel in the public sectors and an increased scarcity of vital medical supplies in health system including HIV medication [18,19]. The adjusted sub-hazard ratios (AsHR) were reported. We censored on 31 December 2017 for those who had not experienced the event of interest and at the last observed date for those who had transferred to other health facilities.

We performed a sensitivity analysis of our key findings through multiple imputations of the missing explanatory covariates using Rubin's method, which assumes that the data are missing at random (MAR) [20]. We compared imputed data and the complete case data estimates based on the maximum likelihood estimation (MLE) perspective for both the Cox proportional hazard and Gombertz parametric models. Within the Gombertz parametric models, estimates from the Bayesian estimation (BE) and MLE were compared [21]. We assumed non-informative normal priors for all unknown parameters to be estimated with a mean of zero and variance of 100 or precision of 0.001 for the parametric Bayesian model. We fitted a parametric Weibull model with random effects to account for any individual-heterogeneity[22]. Model comparison between BE and MLE was done using the Akaike's information criterion (AIC), Bayesian information criterion (BIC) and Deviance information criterion (DIC). In contrast, model comparison between complete case analysis and imputed data analysis was done using the precision of the

For peer review only - http://bmjopen.bmj.com/site/about/guidelines.xhtml

confidence interval for the estimates. All data management and analysis were performed using Stata 15.1 (StataCorp, 2017, Stata Statistical Software: Release 15.1, College Station, TX: StataCorp LLC)[23].

Patient and Public involvement

This study acknowledges the importance of patients and public involvement in research studies; however, this research was done without patient involvement in all research stages since it was secondary data analysis.

Ethical Approval

Ethics approval for this study was granted by The University of Witwatersrand's Human Research Ethics Committee (Medical) (Clearance Certificate No. M170673). Data approval was obtained from the Ministry of Health and Child Care in Zimbabwe, who are the custodians of the countrywide database. Since this study was secondary data analysis, we did not have direct contact with the participants. We received only anonymised data for analyses.

RESULTS

Descriptive Analysis

At the end of study 268,896 (68.81%) patients were alive, 18,328(4.7%) had died, 88,744 (22.71%) were LTFU and 14,803(3.79%) had transferred out. Over 1,544,468 person-years of ART, mortality and LTFU rates were 1.19(95%CI: 1.17-1.20) and 5.75(95%CI: 5.71-5.78) per 100 person-years, respectively. The quarterly trend analysis graph of LTFU over time showed a general increase in LTFU over time with significant peaks in 2012, 2014 and 2016 (Figure 3).

[Insert Figure 3]

The descriptive characteristics of 390,771 patients from 538 health facilities are presented in Table 1. The study participants were enrolled at PHC (45.1%), districts/mission (49.2%) or provincial/referral (5.7%) hospitals. Most patients were enrolled in rural health facilities (66.82%). At ART initiation, majority of the patients were female 117,567(66.7%) with mean (standard deviation) age of 37.5(10.2) years and weight of 60.2(12.3) Kg.

For peer review only - http://bmjopen.bmj.com/site/about/guidelines.xhtml

Table 1: Patients sociodemographic and clinical characteristics at ART initiation in Zimbabwe national ART programme, 2004-2017

Characteristics	Categories	Primary health care (PHC) n=176 253 (45.1%)	District/Mission hospitals n=192 238(49.2%)	Provincial/Referral hospitals n=22 280 (5.7%)	Totals N=390 771 (100%)
**Patient Demographics**					
Age (years) at ART initiation	15-24	20 314 (11.53%)	21 057 (10.95%)	2 601 (11.67%)	43 972 (11.25%)
	25-34	54 416 (30.87%)	58 874 (30.63%)	6 979 (31.32%)	120 269 (30.78%)
	35-44	59 284 (33.64%)	66 418 (34.55%)	7 875 (35.35%)	133 577 (34.18%)
	45+	42 239 (23.96%)	45 889 (23.87%)	4 825 (21.66%)	92 953 (23.79%)
Degree of urbanisation	Rural	113 738 (66.92%)	137 380 (73.57%)	432 (2.01%)	251 550 (66.52%)
	Urban	56 223 (33.08%)	49 348 (26.43%)	21 055 (97.99%)	126 626 (33.48%)
Sex	Females	117 567 (66.70%)	124 141 (64.58%)	14 136 (63.45%)	255 844 (65.47%)
	Male	58 686 (33.30%)	68 097 (35.42%)	8 144 (36.55%)	134 927 (34.53%)
Education status at ART initiation	None	3 854 (2.19%)	4 136 (2.15%)	227 (1.02%)	8 217 (2.10%)
	Primary	33 735 (19.14%)	34 062 (17.72%)	2 248 (10.09%)	70 045 (17.92%)
	Secondary	59 181(33.58%)	58 349 (30.35)	6 593 (29.59%)	124 123 (31.76%)
	Tertiary	2 921(1.66%)	4 423(2.30%)	1 177(5.28%)	8 521 (2.18%)
	Missing	76 562(43.44%)	91 268 (47.48%)	12 035(54.02)	179 865 (46.03%)
Marital status at ART initiation	Single	24 438 (13.87%)	24 489 (12.74%)	3 530 (15.84%)	52 457 (13.42%)
	Married	105 504 (59.86%)	113 953 (59.28%)	12 935 (58.06%)	232 392 (59.47%)
	Widowed	25 798 (14.64%)	30 994 (16.12%)	3 523 (15.81%)	3 523 (15.81%)
	Divorced	13 429 (7.62%)	14 732 (7.66%)	1 677 (7.53%)	29 838 (7.64%)
	Missing	7 084(4.02%)	8 070 (4.20%)	615 (2.76%)	15 769 (4.04%)
Calendar time of starting ART	2004-2007	6 635 (3.76%)	8 942 (4.65%)	2 962 (13.29%)	18539 (4.74%)
	2008-2011	38 990 (22.12%)	61 321 (31.85%)	7 899 (35.45%)	108120 (27.67%)
	2012-2014	67 759 (38.44%)	90 582 (47.12%)	9 472 (42.51%)	167 813 (42.94%)
	2015-2017	62 869 (35.67%)	31 483 (16.38%)	1 947 (8.74%)	96 299 (24.64%)
**Clinical factors**					
WHO staging at ART initiation	I/II	82 203 (46.64%)	69 629 (36.22%)	7 690 (34.52%)	159 519 (40.82%)
	III/IV	79 279 (44.98%)	118 113 (61.44%)	14 425 (64.74%)	211 817 (54.20%)
	Missing	14 771 (8.38%)	4 499 (2.34%)	165 (0.74%)	19 435 (4.97%)
Baseline CD4 (cell/uL) at ART initiation	Above 500	4 783 (2.71%)	4 950 (2.57%)	601 (2.70%)	10 334 (2.64%)
	351-500	8 227 (4.67%)	8 790 (4.57%)	940 (4.22%)	17 957 (4.60%)
	200-350	17 908 (10.16%)	24 895 (12.95%)	2 866 (12.86%)	45 669 (11.69%)
	Below 200	23 796 (13.50%)	35 020 (18.22%)	4 982 (22.36%)	63 798 (16.33%)
	Missing	121 539 (68.96%)	118 583 (61.69%)	12 891 (57.86%)	253 013 (64.75%)
Treatment regimen at the time of data extraction	First-line	170 662 (98.29%)	184 321 (97.37%)	20 724 (94.32%)	375 704 (97.65%)
	D4T(30)+3TC+NVP	625 (0.35%)	2 942 (1.53%)	629 (2.82%)	4 196 (1.07%)
	D4T(30)+ 3TC+EFV	42 (0.02%)	155 (0.08%)	20 (0.09)	217(0.06%)
	AZT+3TC+NVP	1 349 (0.77%)	1 933 (1.01%)	349 (1.57%)	3 631(0.93%)
	AZT+3TC+EFT	212 (0.12%)	425 (0.22%)	56 (0.25 %)	693 (0.18%)
	TDF+3TC+NVP	6 541 (3.71%)	16 334 (8.50%)	2 923 (13.12%)	25 798 (6.60%)
	TDF+3TC+EFV	161 288 (91.51%)	162 006 (84.27%)	16 628 (4.63%)	339 922 (86.99%)
	Other first lines	3 236 (1.84%)	3 474 (1.81%)	428 (1.92%)	7 138 (1.83%)
	Second line	2 960 (1.68%)	4 969 (2.58%)	1 247(5.60%)	9 176 (2.35%)
Tuberculosis status at ART initiation	Negative	156 072 (88.55%)	170 869 (88.88%)	20 218 (90.75%)	347 159 (88.84%)
	Positive	2 092 (1.19%)	2 423 (1.26%)	301 (1.35%)	4 816 (1.23%)
	Not assessed	15 743 (8 93%)	16 387 (8.52%)	1 347 (6.05%)	33 477 (8.57%)
	Missing	2 346 (1.33%)	2 559 (1.33%)	414 (1.86%)	5 319 (1.36%)
Functional status at ART initiation	Working	162 710 (92.32%)	180 417 (93.85%)	20 024 (89.87%)	363 151 (92.93%)
	Ambulatory	11 789 (6.69%)	10 454 (5.44%)	2 089 (9.38%)	24 332 (6.23%)
	Bedridden	331 (0.19%)	452 (0.24 %)	28(0.13%)	811 (0.21%)
	Missing	1 423 (0.81%)	915 (0.48%)	139(0.62%)	2 477 (0.63%)

For peer review only

For peer review only - http://bmjopen.bmj.com/site/about/guidelines.xhtml

Most patients were married (59.5%) with secondary education (31.8%) while 54.2% were classified in WHO stage III or IV and 34.18% were in the 35-44 years age group. Majority of the participants were mainly on first-line ART treatment (97.7%), and few had confirmed tuberculosis infection (1.2%). There were missing data on some covariates, including baseline CD4 cell counts recorded for only 35.3% of the study participants**.**

The time-to-event rates for LTFU stratified by baseline characteristics are shown in Table 2. LTFU rate per 100-person-years was 5.92 for patients enrolled at district and mission hospitals; 6.60 amongst patients from rural health facilities and 17.09 among patients diagnosed with tuberculosis.

Table 2: Rates per 100 person-years of loss to follow-up and log-rank tests for ART patients' baseline characteristics in Zimbabwe national ART programme, 2004-2017

Characteristics	Category	LTFU cases	Person-years	Rate per 100 person-years	95% CI of rate	Log-rank p-value
Health facility level	Primary health care	34 958	6 200	5.6502	5.5913 to 5.7097	
	District/mission hospitals	48 188	8 100	5.9169	5.8643 to 5.9700	<0.001*
	Provincial/referral hospitals	5 598	1 100	5.0273	4.8973 to 5.1608	
Age (years) at ART initiation	15-24	32 790	4 500	7.3252	7.2463 to 7.4049	
	25-34	12 617	1 500	8.4301	8.2843 to 8.5785	
	35-44	27 504	5 500	4.9671	4.9087 to 5.0261	<0.001*
	45+	15 833	3 900	4.0242	3.9620 to 4.0874	
Degree of urbanisation	Rural	52 629	10 000	5.2756	5.2307 to 5.3209	
	Urban	36 115	5 500	6.6039	6.5362 to 6.6724	<0.001*
Sex	Females	59 695	10 000	5.8703	5.8234 to 5.9175	
	Male	29 049	5 300	5.5063	5.4433 to 5.5700	<0.001*
Education status at ART initiation	None	1 470	354	4.1582	3.9509 to 4.3762	
	Primary	14 786	2 800	5.3558	5.2702 to 5.4429	
	Secondary	32 629	4 700	6.9961	6.9206 to 7.0725	<0.001*
	Tertiary	1 803	409	4.4097	4.2108 to 4.6180	
	Missing	38 056	7 300	5.2435	5.1911 to 5.2965	
Marital status at ART initiation	Single	13 078	1 900	6.9036	6.7863 to 7.0230	
	Married	7 548	1 100	6.6112	6.4637 to 6.7620	
	Widowed	9 375	2 900	3.1872	3.1233 to 3.2524	<0.001*
	Divorced	55 985	8 800	6.3313	6.2790 to 6.3839	
	Missing	2 758	625	4.4160	4.2543 to 4.5840	
Calendar time of starting ART	2004-2007	790	1 900	0.4255	0.3968 to 0.4562	
	2008-2011	4 572	6 900	0.6593	0.6405 to 0.6787	
	2012-2014	43 565	5 300	8.1802	8.1038 to 8.2574	<0.001*
	2015-2017	39 817	1 300	29.9903	29.6972 to 30.2863	
WHO staging at ART initiation	I/II	47 410	5 100	9.2080	9.1255 to 9.2912	
	III/IV	38 919	9 500	4.0957	4.0552 to 4.1366	<0.001*
	Missing	2 415	794	3.0432	2.9242 to 3.1671	
Baseline CD4 (cell/uL) at ART initiation	Above 500	4 341	257	16.9190	16.4231 to 17.4299	
	351-500	7 614	455	16.7362	16.3645 to 17.1164	
	200-350	11 556	1 700	6.7428	6.6210 to 6.8669	<0.001*
	Below 200	15 271	2 700	5.7338	5.6436 to 5.8254	

For peer review only - http://bmjopen.bmj.com/site/about/guidelines.xhtml

	Missing	49 962	10 000	4.8245	4.7823 to 4.8669	
Treatment regimen at the time of data extraction	First line	88 050	15 000	5.9066	5.8677 to 5.9457	
	D4T(30)+ 3TC+NVP	2 685	168	15.9847	15.3914 to 16.6009	
	D4T(30)+ 3TC+EFV	141	10	14.7353	12.4932 to 17.3797	
	AZT+3TC+NVP	448	184	2.4374	2.2218 to 2.6738	
	AZT+3TC+EFT	158	30	5.3159	4.5484 to 6.2129	<0.001*
	TDF+3TC+NVP	9 652	1 100	9.1737	8.9925 to 9.3586	
	TDF+3TC+EFV	71 378	13 000	5.3710	5.3318 to 5.4106	
	Other first lines	3 588	175	20.5590	19.8972 to 21.2429	
	Second line	694	538	1.2900	1.1985 to 1.3908	
Tuberculosis status at ART initiation	Negative	81 119	14 000	5.9418	5.9010 to 5.9828	
	Positive	2 235	131	17.0906	16.3966 to 17.8141	
	Not assessed	4 188	1 400	2.9328	2.8453 to 3.0230	<0.001*
	Missing	1 202	234	5.1455	4.8627 to 5.4447	
Functional status at ART initiation	Working	81 122	14 000	5.6445	5.6057 to 5.6834	
	Ambulatory	7 059	930	7.5897	7.4147 to 7.7688	
	Bedridden	215	26	8.4201	7.3666 to 9.6243	<0.001*
	Missing	348	117	2.9723	2.6758 to 3.3015	

*Significant at 5%

The cumulative incidence probability (CIP) curve for mortality outcome was lower compared to the LTFU outcome (Figure 4A). The LTFU CIP curve increased in the first 3 years on ART and became steady after that. We observed overwhelmingly significant variation between LTFU and selected covariates, log-rank p-value<0.001. Higher risk of LTFU was observed amongst 15-24 years patients (Figure 4B), females (Figure 4C), patients enrolled at urban health facilities (Figure 4D); tuberculosis-infected patients (Figure 4E) and WHO stage I/II patients (Figure 4F).

[Insert Figure 4]

Loss to Follow-up Outcome

To determine the possible independent risk factors associated with LTFU, seven models were fitted, and the results are shown in Table 3. An additional pictorial view of the estimates is provided in the Appendix section (Figure A1) to compare the different model estimates effectively. The data had approximately 10% of missing information in the explanatory covariates. There was no difference in the model estimates between imputed data and complete case data (model 1 vs model 2 and model 4 vs model 5). Comparing the MLE (model 3) and the BE (model 4) models, the estimates were almost similar for most regression parameters; and inclusion of the individual random effects did not improve the model estimates (model 4 vs model 6). Lastly, adjusting for the competing effects of mortality on LTFU showed some significant improvement to the model based on the reduced

For peer review only

For peer review only - http://bmjopen.bmj.com/site/about/guidelines.xhtml

Table 3: Multiple regression models results of LTFU amongst ART patients in Zimbabwe national ART programme, 2004-2017 for different models

Characteristics	Category	Cox proportional hazard		Parametric Gompertz model			Weibull model with random effects	Competing risk model of mortality on LTFU
		CC + MLE (model 1)	MI+ MLE (model 2)	CC+BE (model 3)	CC+MLE (model 4)	MI+MLE (model 5)	CC+MLE (model 6)	CC+MLE (model 7)
Health facility level	Primary health care	1(reference)	1(reference)	1(reference)	1(reference)	1(reference)	1(reference)	1(reference)
	Secondary hospitals[a]	1.48(1.46-1.50)	1.51(1.49-1.53)	1.53(1.51-1.54)	1.53(1.51-1.55)	1.57(1.54-1.59)	1.48(1.45-1.51)	1.47(1.45-1.50)
	Tertiary hospitals[b]	1.17(1.14-1.21)	1.23(1.20-1.27)	1.23(1.21-1.25)	1.23(1.20-1.27)	1.30(1.26-1.34)	1.10(1.06-1.14)	1.22(1.18-1.26)
Age (years) at ART initiation	15-24	1.16(1.13-1.19)	1.17(1.14-1.19)	1.12(1.11-1.14)	1.13(1.10-1.15)	1.14(1.12-1.17)	1.23(1.20-1.27)	1.19(1.16-1.21)
	25-34	1.18(1.17-1.20)	1.19(1.17-1.21)	1.18(1.16-1.19)	1.18(1.16-1.20)	1.19(1.17-1.20)	1.24(1.21-1.27)	1.19(1.17-1.21)
	35-44	1(reference)	1(reference)	1(reference)	1(reference)	1(reference)	1(reference)	1(reference)
	45+	0.88(0.87-0.89)	0.87(0.86-0.89)	0.88(0.87-0.89)	0.88(0.86-0.90)	0.87(0.85-0.88)	0.86(0.84-0.88)	0.87(0.85-0.89)
Degree of urbanisation	Rural	1(reference)	1(reference)	1(reference)	1(reference)	1(reference)	1(reference)	1(reference)
	Urban	1.70(1.68-1.72)	1.67(1.65-1.27)	1.67(1.65-1.69)	1.67(1.65-1.70)	1.64(1.61-1.66)	2.06(2.01-2.10)	1.61(1.59-1.63)
Calendar time of starting ART	2004-2007	0.04(0.04-0.05)	0.04(0.04-0.05)	0.02(0.02-2.02)	0.02(0.01-0.02)	0.02(0.01-0.02)	0.01(0.01-0.01)	0.09(0.09-0.10)
	2008-2011	0.07(.0.7-0.08)	0.07(0.07-0.08)	0.04(0.04-0.04)	0.04(0.04-0.04)	0.05(0.04-0.05)	0.02(0.02-0.02)	0.11(0.10-0.11)
	2012-2014	1(reference)	1(reference)	1(reference)	1(reference)	1(reference)	1(reference)	1(reference)
	2015-2017	8.48(8.34-8.63)	8.40(8.26-8.54)	5.95(5.90-6.00)	5.97(5.88-6.06)	5.90(5.81-5.99)	16.09(15.56-16.63)	6.02(5.91-6.12)
WHO staging at ART initiation	I/II	1(reference)	1(reference)	1(reference)	1(reference)	1(reference)	1(reference)	1(reference)
	III/IV	0.73(0.72-0.74)	0.74(0.73-0.75)	0.73(0.73-0.74)	0.73(0.72-0.75)	0.74(0.73-0.75)	0.67(0.65-0.68)	0.74(0.73-0.75)
Treatment regimen at the time of data extraction	D4T(30)+ 3TC+NVP	5.96(5.72-6.21)	6.00(5.77-6.25)	6.065.92-6.22)	6.09(5.84-6.34)	6.06(5.82-6.30)	15.05(13.98-16.21)	5.47(5.52-5.75)
	D4T(30)+ 3TC+EFV	8.55(7.21-10.12)	8.34(7.09-9.90)	8.71(8.21-9.20)	8.47(7.16-10.03)	8.29(7.01-9.79)	19.88(15.23-25.87)	7.98(6.41-9.94)
	AZT+3TC+NVP	0.73(0.67-0.80)	0.72(0.66-0.79)	0.74(0.71-0.78)	0.74(0.67-0.81)	0.72(0.65-0.79)	0.74(0.66-0.83)	0.75(0.68-0.83)
	AZT+3TC+EFV	1.19(1.02-1.40)	1.19(1.01-1.39)	1.19(1.16-1.23)	1.21(1.03-1.42)	1.21(1.03-1.40)	1.27(1.03-1.56)	1.09(0.91-1.30)
	TDF+3TC+NVP	2.41(2.36-2.47)	2.46(2.40-2.51)	2.46(2.42-2.50)	2.45(2.40-2.51)	2.50(2.44-2.55)	3.58(3.45-3.70)	2.29(2.23-2.34)
	TDF+3TC+EFV	1(reference)	1(reference)	1(reference)	1(reference)	1(reference)	1(reference)	1(reference)
	Other first lines	1.97(1.90-2.04)	1.97(1.91-2.04)	1.88(1.83-1.93)	1.89(1.82-1.95)	1.89(1.83-1.96)	2.44(2.32-2.57)	1.61(1.54-1.68)
	Second line	0.46(0.42-0.49)	0.45(0.42-0.49)	0.45(0.43-0.47)	0.44(0.41-0.48)	0.44(0.41-0.47)	0.39(0.36-0.42)	0.48(0.44-0.52)
Tuberculosis status at ART initiation	Negative	1(reference)	1(reference)	1(reference)	1(reference)	1(reference)	1(reference)	1(reference)
	Positive	2.14(2.05-2.24)	2.13(2.04-2.23)	2.03(1.99-2.09)	2.02(1.94-2.11)	2.01(1.93-2.10)	2.66(2.50-2.83)	1.53(1.45-1.62)
	Not assessed	0.58(0.56-0.59)	0.59(0.57-0.61)	0.60(0.59-0.60)	0.60(0.58-0.62)	0.61(0.59-0.63)	0.51(0.49-0.53)	0.60(0.58-0.62)
Functional status at ART initiation	Working	1(reference)	1(reference)	1(reference)	1(reference)	1(reference)	1(reference)	1(reference)
	Ambulatory	1.22(1.19-1.24)	1.23(1.20-1.26)	1.23(1.21-1.26)	1.23(1.20-1.27)	1.25(1.22-1.28)	1.28(1.23-1.32)	1.08(1.05-1.11)
	Bedridden	1.34(1.17-1.53)	1.33(1.16-1.52)	1.32(1.29-1.35)	1.34(1.16-1.53)	1.32(1.15-1.51)	1.42(1.18-1.72)	0.64(0.54-0.75)
	AIC	196416.4			379948.9		445559.7	**195708.7**
	BIC	196439.1			380197.6		445819.3	**195731.4**
	DIC			379925.9				

CC=complete case, MI=multiple imputation, BE=Bayesian estimation, MLE=maximum likelihood estimation, Bold face= low values of the information criterion

[a] District and mission hospitals; [b] Provincial and referral hospitals

For peer review only - http://bmjopen.bmj.com/site/about/guidelines.xhtml

AIC and BIC values compared to the Cox proportional hazard model. Hence, the competing risk model was considered a better fit compared to other models; and results interpretation will be based on this model.

The multivariable competing risk model revealed that patient factors predicting LTFU included: being enrolled at a provincial/referral (AsHR 1.22; 95%CI: 1.18-1.26) or district/mission (AsHR 1.47; 95%CI: 1.45-1.50) hospitals (reference: PHC), urban sites (AsHR 1.61; 95%CI:1.59-1.63) (reference: rural) and 15-24 years group (AsHR 1.19; 95%CI: 1.16-1.21) (reference: 35-44 years). We also detected an overwhelming association between LTFU and tuberculosis-infected patients (AsHR 1.53; 95%CI: 1.45-1.62) (reference: no tuberculosis) and a protective effect of LTFU for patients classified in WHO stage III or IV (AsHR 0.74; 95%CI: 0.73-0.75) (reference: WHO I/II). Patients who were taking stavudine-combination therapy as a first-line ART treatment had an increased risk of becoming LTFU relative to patients taking TDF+3TC+EFV first-line combination therapy, that is, D4T(30)+3TC+NVP (AsHR 5.47; 95%CI:5.52-5.75) and D4T(30)+3TC+EFV (AsHR 7.98; 95%CI: 6.41-9.94). We also observed that patients who initiated ART between 2015 and 2017 had a pronounced risk of becoming LTFU compared to patients who enrolled between 2012 and 2014 (AsHR 6.02; 95%CI: 5.91-6.12).

DISCUSSION

This study aims to determine the loss to follow-up (LTFU) rates at different health care levels after ART services decentralisation among ART patients who initiated ART between 2004 and 2017 and determine other potential independent factors using the competing risk model approach. Our study followed similar studies adjusting for the competing effect of mortality on LTFU, recognising that those who die are no longer at risk of LTFU [24,25]. We found the LTFU rate of 5.7 per 100- person-years, which was lower than that reported in Ethiopia of 8.2 per 100 person-years [26]. The lower LTFU rates in our study put forward the positive impact of the differential models implemented in Zimbabwe to increase patients' retention in HIV care. However, there is still a considerable magnitude of LTFU outcome in ART programs contributing to the "leakages" or attrition of patients within the continuum of care cascade in Zimbabwe. This requires continual upscaling of interventions which minimise the LTFU rates and retain patients in care since patients who are LTFU are associated with increased treatment

For peer review only - http://bmjopen.bmj.com/site/about/guidelines.xhtml

interruptions which can trigger ART treatment defaulting, treatment non-adherence, treatment failure or treatment resistance [27,28].

Similar to other studies, LTFU independently increased at higher levels of care (provincial and referral (AsHR 1.22; 95%CI: 1.18-1.26) and district and mission (AsHR 1.47; 95%CI: 1.45-1.50) hospitals) relative to PHC in this study [5]. This finding supports the fact that decentralisation is associated with better patients' retention in care as there are high risks of LTFU rate in higher levels of care compared to the PHC facilities. This finding supports the differential care mechanism that if patients seek care from health facilities closer to their homes with cheaper transport cost and less congested hospital environments, there is improved patient retention as compared to seeking care to faraway health facilities [29]. Moreover, the high rates of LTFU in secondary and tertiary/quaternary levels of care could be explained by "silent transfers" to these decentralised PHC facilities based on individual preferences of where to seek care which could be a good indication of the upsurge of differential care mechanism [29]. However, such "silent transfers" may result in inflated LTFU rates in higher levels of care as most these patients would be classified as LTFU when in fact they might have self-transferred and the patient tracking system is not efficient enough to pick such scenarios due to significantly high patients' volumes persisting at these health facilities [5]. Similarly, the quarterly trend analysis of LTFU rates showed an increase over time, peaking in the period when deferential models were implemented. The same issue of "silent transfers" of patients to nearer health facilities can also explain this trend pattern finding.

On the contrary, not everyone would prefer to seek care from these decentralised health facilities. Some may intentionally avoid these nearby health facilities to conceal their HIV status from the community. These individuals remain enrolled in faraway health facilities and most probably the higher levels of care. These individuals may contribute to the observed high rated of LTFU in this cohort as they may still face transport cost challenges to replenish their ART supplies[30]. Moreover, tertiary and quaternary levels of care may receive much sicker patients with a high risk of death, hoping to get better treatment through the health care referral system [31]. This means some of these much ill patients may become LTFU eventually as severe immune deterioration hinders one to keep up to date with their treatment replenishments. Earlier studies have also reported that much sicker patients with severe immune deterioration are more likely to

For peer review only - http://bmjopen.bmj.com/site/about/guidelines.xhtml

become LTFU compared to those with no significant immunodeficiency [32]. Other studies have also shown that a significant portion of these severely ill patients who eventually become LTFU from higher levels of care health facilities, if tracked, could be found dead [33]. Our findings put forward that though the decentralisation uptake might have been taken up by the majority of the ART patients and improved patient's retention in the ART program in overall; those groups of individuals who still seek health care from the higher levels of care are still more at risk of becoming LTFU; hence, interventions which may retain patients in the higher levels of care facilities are a priority.

Patients enrolled at urban health facilities had increased risk to become LTFU compared to those at rural sites. This finding contradicts a study done in Ethiopia whereby rural residents had an increased risk of LTFU due to availability of transport costs, long distances to ART health facilities, social stigma as well as low levels of HIV knowledge in these communities[34]. However, our study finding can be explained by the decentralisation of health facilities to walkable distances in favour of rural residents [4,35]. Mobility of residents in urban areas is disproportionate relative to rural residents; hence, most of these LTFU in urban areas could be part of the silent transfers to other nearer ART health facilities in their relocated areas.

Another interesting finding was high LTFU among the 15-24 years group. The adolescent's group is known to be associated with additional intricacy in their HIV management due to different types of chronic comorbidities, and this whole complexity may impact on their ART outcomes including LTFU [36,37]. The high risk of becoming LTFU among adolescents can be attributed to structural deprivation due to medical and psychological reasons which contribute to poor adherence in this subpopulation group as a result of becoming LTFU [38]. The most common medical barrier which has been found to affect ART adherence include treatment side effects, multiple doses and health system dissatisfaction. Research has found that ART has improved perinatal HIV infection into a controllable disease through optimal ART adherence. This opens up an additional challenge among adolescents who were prenatally or perinatally infected in the transition stage to adults care to experience ART adherence lapses and develop worse clinical outcomes as they become LTFU [38]. The transition from paediatric care to adult care would mean changing care providers, absence of youth-friendly facilities, tight and hectic schedules, new responsibilities and deprivation of caregiver support for some of these

For peer review only - http://bmjopen.bmj.com/site/about/guidelines.xhtml

adolescents. Some of the social deprivation issues among adolescents include inadequate financial support to cover transport costs to health facilities for treatment refill since this group heavily depends on their caregivers [39]. Some of the social deprivations faced by adolescents include lack of family support as some adolescents come from disrupted family structures with depraved caregiver-child relationships; fear perceived HIV-related stigma and discrimination to disclose their status [34,39,40].

Therefore, this study recommends the policymakers to strengthen the social protection support policy which addresses complex vulnerability, disadvantages and risks faced by HIV positive adolescents. This strategy also may foster resiliencies in general, HIV positive adolescents' population. The social protection support can interrupt some of the social deprivations faced by adolescents through poverty reduction and economic development, improved access to health care, access to health services, reduced stigma and discrimination and improved caregiver psychosocial and physical well-being. Another recommendation is the use of health facility-based peer-supporters programmes for adolescents to improve ART adherence and ultimately, viral suppression[41,42]. The peer support activities involve peer-to-peer counselling, peer support groups and treatment buddy programmes among adolescents with the aim of ART adherence and ultimately achieving viral suppression, reducing the HIV-related illnesses among adolescents. As adolescents may exit the HIV care through becoming LTFU or withdrawals due to transition adjustments challenges from paediatric care to adult care; an early multidisciplinary and developmentally suitable transition preparedness mechanism is an alternative. The mechanism should be a comprehensive package addressing inherent adolescents' health issues like cognitive health, ART adherence, stigma, disclosure and socioeconomic issues [36,42]. Besides, a simple regimen, directly observed therapy and technological interventions like cell phone reminders have been found to increase ART adherence in adolescents.

We observed an overwhelming association of tuberculosis infection and becoming LTFU which concurs with an earlier study which reported that the risk of becoming LTFU was two times more likely in HIV patients with tuberculosis infection compared to those who did not have tuberculosis [26]. This finding can be explained by overlapping toxicity of HIV and tuberculosis drugs, leading to adverse reactions which have a direct consequence of becoming LTFU [26]. Therefore, a push towards integrated care of HIV and tuberculosis service delivery would elevate

For peer review only

For peer review only - http://bmjopen.bmj.com/site/about/guidelines.xhtml

HIV uptake and tuberculosis screening and reduce travel inconveniences on patients [31,43]. Also, tuberculosis treatment before ART initiation reduces the risk to LTFU [40]; hence, it is profound to increase efforts in tuberculosis screening and treatment of HIV patients. However, issues around tuberculosis drug resistance cannot be ignored. Nowadays, HIV studies also consider information on non-communicable diseases like cancer, diabetes and hypertension; however, this information was missing from this current study database.

We also detected that patients classified in WHO stage III or IV were less likely to become LTFU relative to those classified in WHO stage I or II. However, our finding contradicts with earlier studies which have reported that experiencing a severe AIDS-defining ailment was associated with reduced risk of becoming LTFU[24,44]. Our study finding may put forward the fact that there could have been an upsurge of health-seeking behaviour amongst patients with advanced WHO clinical stages in this database cohort or this finding could be attributed to expansions of community awareness through health promotion in these health facilities communities.

Strengths and weaknesses of this study

This study analyses employed have some implications; we used the regularisation variable selection technique to identify the important covariates to be used. The lasso model shrinks the variable coefficient to zero and produces a simpler model which can be interpreted easily. For the survival model, various model types were fitted including the standard Cox regression model; however, due to its overestimation of the risk in the presence of competing risks, we considered the Fine and Gray competing risk model [45]. We have seen that ignoring competing risks alters the probabilities of occurrence of the outcome giving biased estimates. Standard survival methods normally used in many studies censor the competing risk; however, this is improper because those who have died can no longer become LTFU. Such analysis will either overestimate or underestimate the risk ratios. We also used individual data which enabled exploration and adjustment for patient-level covariates associated with the outcome to get robust information on the ART outcome patterns as compared to aggregated data[30,46]. The follow-up period in our study was relatively long (13years), and the dataset used was hugely represented all the ten provinces in Zimbabwe without restriction to specific geographical areas. The data

For peer review only - http://bmjopen.bmj.com/site/about/guidelines.xhtml

represented quaternary, tertiary, secondary and primary health facilities from private and public sectors. This makes our results generalisable to the Zimbabwe setting since the data reflect a true representation of all HIV structured in the country. To the best of our knowledge, this study could be one of the largest dataset used for Zimbabwe to analyse adult ART outcomes at the patient-level.

Our study results should be inferred in light of some limitations. Missing data was of concern, particularly to those variables that influence LTFU like viral load and CD4 cell count in addition to non-communicable diseases comorbidities. The presents of missing data could be explained by poor documentation of information [31] or limited access to the point of care machines for viral load, and CD4 cell counts measurements [4,47,48]. The missing patterns for some covariates like viral load and CD4 cell count measurements were likely to be informative and missing not at random (MNAR) since differentiated care monitoring approach was used in some years covered by this study[49]. Therefore, to maintain sufficient power, covariates with a high percentage of missing observations were excluded at the multivariable analysis stage. In future, we recommend that the data clerks should be trained on the importance of capturing full information and to the programme managers to consider expanding the HIV routine data collection tool to capture non-communication diseases conditions of the HIV patients. The socio-demographic and clinical characteristics in the database were only taken at baseline or last observed at the time of data extraction; hence, we could not incorporate the time dependency of some covariates. We also acknowledge that there could be differential misclassification of the LTFU outcome in this study in the sense that some of those recorded as LTFU may be “silent transfers” who have enrolled in a different health facility as a result of ART decentralisation and have been given a different unique identifier number; hence, this might have biased our LTFU estimates in this study. There is also a possibility of participant selection bias since we only considered patients whose information as captured through the ePMS; as a result, the reported findings could be systematically different from those patients not included. However, it is of paramount importance to note that the dataset used was huge, and representation of the health facilities in Zimbabwe was reflected; hence, our findings could be generalised to all ART services health facilities in Zimbabwe.

For peer review only - http://bmjopen.bmj.com/site/about/guidelines.xhtml

While our findings for the common predictors of LTFU may be comparable to earlier studies conducted in Sub- Saharan Africa region, we had also hypothesised that those ART patients' who enrolled around 2008 would have a higher risk of becoming LTFU. This was done taking note of the hyperinflation that occurred in Zimbabwe during the part of the study period under review. The economic turmoil was allied to the increased mass departure of qualified health professionals and scarcities of vital medical supplies in health facilities [18,19]. Despite this situation, we detected a protective effect of becoming LTFU between 2008 and 2011 in this study. This could be explained by the support the country received through Global Fund [4], and ART sites increased from 282 sites in 2008 to 1,006 in 2012 [35].

Conclusion

The data for the 13-year window agree with the evident decline of the HIV incidence and prevalence in Zimbabwe, even from Demographic Health Survey. Our results indicate that patient retention in care is still a drawback in HIV prevention and control. As much as most of the observed LTFU outcome at higher levels of care (district, provincial and central hospitals) could be "silent transfers", most patients who become LTFU are likely to have poor ART adherence if not dead. Poor ART adherence leads to poor viral suppression which affects achieving the third 90% UNAIDS targets, which state that 90% of those patients on ART should be virally suppressed. Clinicians should provide continual educational support on the importance of ART adherence to increase life expectancy among the HIV population and other benefits of ART. Policymakers and program managers should come up with strategies which may retain patients on ART including increased patients surveillance and strengthen patients' tracing mechanisms to reduce LTFU at health facilities; strengthening community-based caregiver support [14], use of peers and nutritional aid support especially for adolescents and young (15-34years). In addition to interventions which minimise "leakages" in the HIV treatment cascade including use of ART adherence clubs strategies to reduce ART resistance [50] and sexual abstinence, campaigns should remain the cornerstone of HIV prevention and control. For further studies, we recommend the use of time-varying covariates like regimen type, CD4 cell counts, WHO stages and viral load measurements; and the use of spatial analysis of ART outcome to identify high burdened regions which the policymakers may use to allocate resources, especially in resource-constrained settings.

For peer review only - http://bmjopen.bmj.com/site/about/guidelines.xhtml

Supplementary materials

Additional theoretical aspects of survival models: Cox regression model and Competing risk model

[Insert Appendix 1-supplementary file here]

Hazard ratio plots for the different survival models fitted on complete case data and imputed time to LTFU data

[Insert Appendix 2- Figure A1 here]

Figure A1: Hazard ratio plots for the different survival models fitted on the complete case data and imputed time to becoming LTFU data among ART patients.

Author Contributions: **ZMZ** conceptualised the concept, cleaned and analysed the data and wrote the initial draft of the manuscript**. JT** and **TFC** advised on analysis, **EM** guided and oversaw the analysis and reviewed the manuscript. **ZMZ, JT, TC and EM** contributed to the interpretation of the results. All the authors reviewed the final manuscript for submission.

Funding: This work was supported by the Developing Excellence in Leadership, Training and Science (DELTAS) Africa Initiative Sub-Saharan Africa Consortium for Advanced Biostatistics (SSACAB) [Grant No. 107754/Z/15/Z]. The DELTAS Africa Initiative is an independent funding scheme of the African Academy of Sciences (AAS) Alliance for Accelerating Excellence in Science in Africa (AESA) and is supported by the New Partnership for Africa's Development Planning and Coordinating Agency (NEPAD Agency) with funding from the Wellcome Trust [Grant No. 107754/Z/15/Z] and the United Kingdom government. The views expressed in this publication are those of the authors and not necessarily those of the AAS, NEPAD Agency, Wellcome Trust, the UK government or the Ministry of Health and Child Care, Zimbabwe.

Acknowledgements: Our acknowledgements go to the Ministry of Health and Child Care, AIDS/TB Units Department for the support in data compilation and extraction for this study. We also thank the Division of Epidemiology and Biostatistics at the School of Public Health for their

For peer review only - http://bmjopen.bmj.com/site/about/guidelines.xhtml

assistance in the getting ethical approval of this study. Thanks to Dr Nomathemba Chandiwana for technical assistance of this work.

Conflict of interest statement: All authors declare that they do not have a conflict of interest for this work.

Data sharing statement: The data used for this study can be found from a third party through an application process to the Zimbabwe Ministry of Health and Child Care through the HIV/AIDS Unit which oversees the data collection and compilation process for the ART program; therefore, the data is not publicly available.

Figure legends

Figure 1: Flowchart of the inclusion criteria for analyses of adult ART patients' enrolled in Zimbabwe ART program followed-up between 2004 and 2017

Figure 2: Schematic illustration of the variable of importance selection steps and subsequent models fitted for sensitivity analysis of the results

Figure 3: Trend analysis of loss to follow-up outcome among patients receiving ART in Zimbabwe National ART Programme on a quarterly time scale, 2004 to 2017

Figure 4: The cumulative incidence probability of mortality and loss to follow-up (A); and Kaplan-Meier curves for loss to follow-up stratified by baseline age categories (B), gender (C), level of urbanisation of the health facilities (D), baseline tuberculosis infection status (E) and baseline World Health Organization clinical stages for ART patients from the Zimbabwe national ART Programme electronic patient monitoring data, 2004 to 2017

REFERENCES

[1] UNAIDS. Global AIDS UPDATE 2016. UNAIDS 2016;17 Suppl 4:S3-11. http://www.who.int/hiv/pub/arv/global-aids-update-2016-pub/en/ [Accessed on 21 January 2018].

[2] Geng EH, Hare CB, Kahn JO, Jain V, Nunnery T Van, Christopoulos KA, et al. The Effect of a " Universal Antiretroviral Therapy " Recommendation on HIV RNA Levels Among HIV-Infected Patients Entering Care With a CD4 Count Greater Than 500 / μL in a Public Health Setting. Clinical Infectious Disease 2012;55:1690–7.

For peer review only - http://bmjopen.bmj.com/site/about/guidelines.xhtml

[3] Kitahata MM, Gange SJ, Abraham AG, Merriman B, Saag MS, Justice AC, et al. Effect of Early versus Deferred Antiretroviral Therapy for HIV on Survival. The New England Journal of Medicine 2009;360:1815–26.

[4] Mutasa-Apollo T, Shiraishi RW, Takarinda KC, Dzangare J, Mugurungi O, Murungu J, et al. Patient retention, clinical outcomes and attrition-associated factors of HIV-infected patients enrolled in Zimbabwe's National Antiretroviral Therapy Programme, 2007-2010. PLoS ONE 2014;9:2007–10.

[5] Rachlis B, Cole DC, van Lettow M, Escobar M, Muula AS, Ahmad F, et al. Follow-Up Visit Patterns in an Antiretroviral Therapy (ART) Programme in Zomba, Malawi. PLoS ONE 2014;9:e101875.

[6] Farahani M, Vable A, Lebelonyane R, Seipone K, Anderson M, Avalos A, et al. Outcomes of the Botswana national HIV/AIDS treatment programme from 2002 to 2010: a longitudinal analysis. The Lancet Global Health 2014;2:e44-50.

[7] Franke MF, Kaigamba F, Socci AR, Hakizamungu M, Patel A, Bagiruwigize E, et al. Improved Retention Associated With Community-Based Accompaniment for Antiretroviral Therapy Delivery in Rural Rwanda. Clinical Infectious Diseases 2013;56:1319–26. doi:10.1093/cid/cis1193.

[8] Somi G, Keogh SC, Todd J, Kilama B, Wringe A, van den Hombergh J, et al. Low mortality risk but high loss to follow-up among patients in the Tanzanian national HIV care and treatment programme. Tropical Medicine & International Health 2012;17:497–506.

[9] UNAIDS. 90-90-90 An ambitious treatment target to help end AIDS epidemic. United Nations 2014. http://www.unaids.org/sites/default/files/media_asset/90-90-90_en.pdf[Accessed 25 January 2019].

[10] Ministry of Health and Child Care, National AIDS Council. Global Aids Response Progress-Fact Track Commitment to End by 2030- Gam Zimbabwe Country Report 2017. UNAIDS 2017:1–24. http://www.unaids.org/sites/default/files/country/documents/ZWE_2018_countryreport.pdf [Accessed on 27 September 2018].

[11] Osika J, Altman D, Ekbladh L, Katz I, Nguyen H, Rosenfeld J, et al. ZIMBABWE HEALTH SYSTEM ASSESSMENT 2010. 2010.

[12] MINISTER OF HEALTH AND CHILD CARE. Zimbabwe Service Availability and Readiness Assessment 2015 Report. Ministry of Health and Child Care 2015:1–145. http://ncuwash.org/newfour/wp-content/uploads/2017/08/Zimbabwe-Service-Availability-and-Readiness-Assessment-Report.pdf [Accessed date 25 May 2020].

[13] Suthar AB, Rutherford GW, Horvath T, Doherty MC, Negussie EK. Improving antiretroviral therapy scale-up and effectiveness through service integration and decentralisation. AIDS 2014;28:S175–85.

For peer review only - http://bmjopen.bmj.com/site/about/guidelines.xhtml

[14] Ferrand RA, Simms V, Dauya E, Bandason T, Mchugh G, Mujuru H, et al. The effect of community-based support for caregivers on the risk of virological failure in children and adolescents with HIV in Harare, Zimbabwe (ZENITH): an open-label, randomised controlled trial. The Lancet Child and Adolescent Health 2017;1:175–83.

[15] Ministry of Health and Child Care. Guidelines for Antiretroviral Therapy for the Prevention and Treatment of HIV in Guidelines for Prevention and Treatment of HIV in Zimbabwe 2013:1–88.

[16] Ministry of Health and Child Care. Zimbabwe Electronic Patient Management System Strategic Plan Document 2012:1–52.

[17] Fine JP, Gray RJ. A Proportional Hazards Model for the Subdistribution of a Competing Risk. Journal of the American Statistical Association 1999;94:496–509.

[18] World Health Organization. Issues and challenges - Progress on the Health-Related MDGs 2015. http://www.aho.afro.who.int/profiles_information/index.php/Zimbabwe:Issues_and_challenges_-_Progress_on_the_Health-Related_MDGs [Accessed 10 October 2018].

[19] Meldrum A. Zimbabwe's health-care system struggles on. Lancet 2008;371:1059–60.

[20] Rubin DB. Inference and missing data. Biometrika 1976;63:581–92. doi:10.1093/biomet/63.3.581.

[21] Little RJ. Calibrated Bayes: A Bayes/frequentist roadmap. American Statistician, 2006;60:213–23. doi:10.1198/000313006X117837.

[22] Pourhoseingholi MA, Hajizadeh E, Moghimi Dehkordi B, Safaee A, Abadi A, Zali MR. Comparing Cox regression and parametric models for survival of patients with gastric carcinoma. Asian Pacific Journal of Cancer Prevention 2007;8:412–6.

[23] StataCorp. Stata Statistical Software: Release 15. College Station, Texas, USA 2017.

[24] Schöni-Affolter F, Keiser O, Mwango A, Stringer J, Ledergerber B, Mulenga L, et al. Estimating loss to follow-up in HIV-infected patients on antiretroviral therapy: The effect of the competing risk of death in Zambia and Switzerland. PLoS ONE 2011;6:2–8.

[25] Assemie MA, Muchie KF, Ayele TA. Incidence and predictors of loss to follow up among HIV - infected adults at Pawi General Hospital, northwest Ethiopia : competing risk regression model. BMC Research Notes 2018;11:1–6.

[26] Tadesse K, Haile F. Predictors of Loss to Follow Up of Patients Enrolled on Antiretroviral Therapy : A Retrospective Cohort Study. AIDS & Clinical Research 2014;5:1–6.

[27] Babo YD, Alemie GA, Fentaye FW. Predictors of first-line antiretroviral therapy failure amongst HIV-infected adult clients at Woldia Hospital, Northeast Ethiopia. PLoS ONE 2017;12:e0187694.

[28] Dewing SF, Mathews C, Lurie M, Kagee A, Padayachee T, Lombard CJ, et al. Predictors

For peer review only - http://bmjopen.bmj.com/site/about/guidelines.xhtml

of poor adherence among people on antiretroviral treatment in Cape Town, South Africa: A case-control study. AIDS Care 2015;27:342–9. doi:10.1080/09540121.2014.994471.Predictors.

[29] Geng EH, Odeny TA, Lyamuya R, Nakiwogga-Muwanga A, Diero L, Bwana M, et al. Retention in care and patient-reported reasons for undocumented transfer or stopping care among HIV-infected patients on antiretroviral therapy in Eastern Africa: Application of a sampling-based approach. Clinical Infectious Diseases 2016;62:935–44.

[30] Fatti G, Grimwood A, Bock P. Better antiretroviral therapy outcomes at primary healthcare facilities: An evaluation of three tiers of ART services in four South African provinces. PLoS ONE 2010;5:1–10.

[31] Molfino L, Kumar AM, Isaakidis P, Van den Bergh R, Khogali M, Hinderaker SG, et al. High attrition among HIV-infected patients with advanced disease treated in an intermediary referral center in Maputo, Mozambique. Global Health Action 2014;7:23758.

[32] Matsena Zingoni Z, Chirwa TF, Todd J, Musenge E. HIV Disease Progression Among Antiretroviral Therapy Patients in Zimbabwe : A Multistate Markov Model. Frontiers in Public Health 2019;7:1–15. doi:10.3389/fpubh.2019.00326.

[33] Fox MP, Rosen S. Patient retention in antiretroviral therapy programs up to three years on treatment in sub-Saharan Africa, 2007-2009: Systematic review. Tropical Medicine and International Health 2010;15:1–15.

[34] Megerso A, Garoma S, Eticha TT, Workineh T, Daba S, Habtamu Z, et al. Predictors of loss to follow-up in antiretroviral treatment for adult patients in the Oromia region, Ethiopia. HIV/AIDS - Research and Palliative Care 2016;8:83–92.

[35] Ministry of Health and Child Care. National HIV Care and Treatment Strategic Plan 2013-2017 and Three Year Operational Plan 2013:1–80.

[36] McHugh G, Simms V, Dauya E, Bandason T, Chonzi P, Metaxa D, et al. Clinical outcomes in children and adolescents initiating antiretroviral therapy in decentralised healthcare settings in Zimbabwe. Journal of the International AIDS Society 2017;20:1–9.

[37] Ferrand RA, Bandason T, Musvaire P, Larke N, Nathoo K, Mujuru H, et al. Causes of acute hospitalisation in adolescence: Burden and spectrum of HIV-related morbidity in a country with an early-onset and severe HIV epidemic: A prospective survey. PLoS Medicine 2010;7:1–11.

[38] Zanoni BC, Mayer KH. The adolescent and young adult HIV cascade of care in the United States: Exaggerated health disparities. AIDS Patient Care and STDs 2014;28:128–35. doi:10.1089/apc.2013.0345.

[39] Maskew M, Fox MP, Evans D, Govindasamy D, Jamieson L, Malete G, et al. Insights into Adherence among a Cohort of Adolescents Aged 12-20 Years in South Africa: Reported Barriers to Antiretroviral Treatment. AIDS Research and Treatment 2016;2016:1–13. doi:http://dx.doi.org/10.1155/2016/4161738.

For peer review only - http://bmjopen.bmj.com/site/about/guidelines.xhtml

[40] Alvarez-Uria G, Naik PK, Pakam R, Midde M. Factors associated with attrition, mortality, and loss to follow up after antiretroviral therapy initiation: Data from an HIV cohort study in India. Global Health Action 2013;6:1–8.

[41] PATA. The Dar es Salaam Call to Action – Peers to Zero. PATA 2016:6. http://teampata.org/wp-content/uploads/2017/10/Dar-Declaration_WEB.pdf[Accessed on 3 February 2020].

[42] Mark D, Lovich R, Walker D, Burdock T, Ronan A, Hatane L. Providing peer support for adolescents and young people living with HIV. Child Survival Working Group 2020:3–6. http://www.childrenandaids.org/sites/default/files/2018-07/12-ProvidingPeerSupport-CSWG.pdf[Accessed on 26 January 2020].

[43] Takarinda KC, Sandy C, Masuka N, Hazangwe P, Choto RC, Mutasa-Apollo T, et al. Factors Associated with Mortality among Patients on TB Treatment in the Southern Region of Zimbabwe, 2013. Hindawi Tuberculosis Research and Treatment Journal 2017;2017:1–11.

[44] Berheto TM, Haile DB, Mohammed S. Predictors of loss to follow-up in patients living with HIV/AIDS after initiation of antiretroviral therapy. North American Journal of Medical Sciences 2014;6:453–459.

[45] Andersen PK, Z SA, Susanne Rosthoj. Competing risks as a multi-state model. Statistical Methods in Medical Research 2002;11:203–15.

[46] Weigel R, Estill J, Egger M, Harries AD, Makombe S, Tweya H, et al. Mortality and loss to follow-up in the first year of ART. AIDS 2012;26:365–73.

[47] Layer EH, Kennedy CE, Beckham SW, Mbwambo JK, Likindikoki S, Davis WW, et al. Multi-level factors affecting entry into and engagement in the HIV continuum of care in Iringa, Tanzania. PLoS ONE 2014;9. doi:10.1371/journal.pone.0104961.

[48] Nyika H, Mugurungi O, Shambira G, Gombe NT, Bangure D, Mungati M, et al. Factors associated with late presentation for HIV / AIDS care in Harare City, Zimbabwe, 2015. BMC Public Health 2016;16:1–7. doi:10.1186/s12889-016-3044-7.

[49] Phillips A, Vojnov L, Cohn J, Roberts T, Ellman T, Bonner K, et al. Sustainable HIV Treatment in Africa through Viral Load-Informed Differentiated Care. Nature 2016;528:S68–S76.

[50] Bock P, Gunst C, Maschilla L, Holtman R, Grobbelaar N, Wademan D, et al. Retention in care and factors critical for effectively implementing antiretroviral adherence clubs in a rural district in South Africa. Journal of the International AIDS Society 2019;22:e25396.

For peer review only - http://bmjopen.bmj.com/site/about/guidelines.xhtml

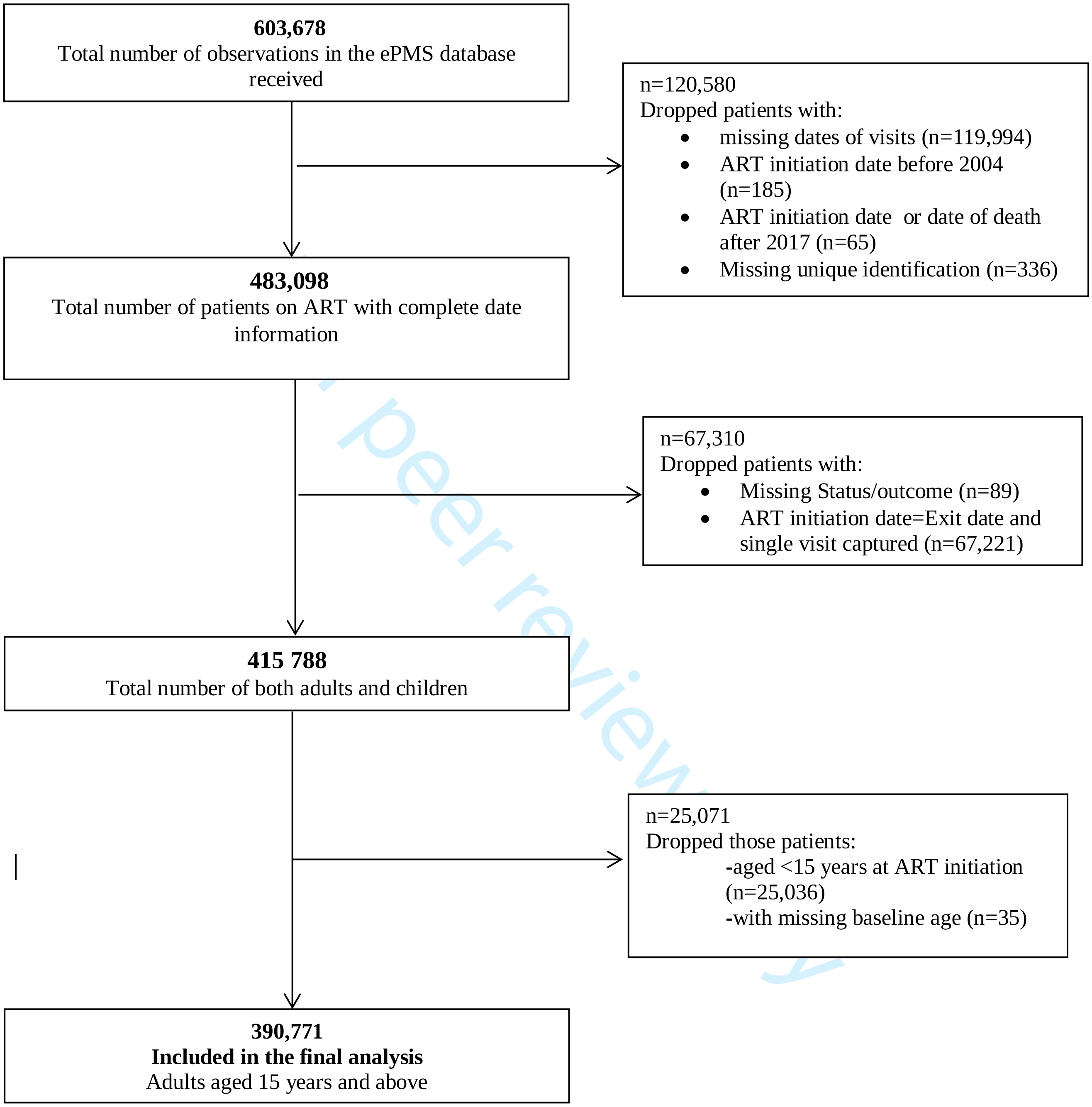

Figure 1: Flowchart of the inclusion criteria for analyses of adult ART patients' enrolled in Zimbabwe ART program followed-up between 2004 and 2017

For peer review only - http://bmjopen.bmj.com/site/about/guidelines.xhtml

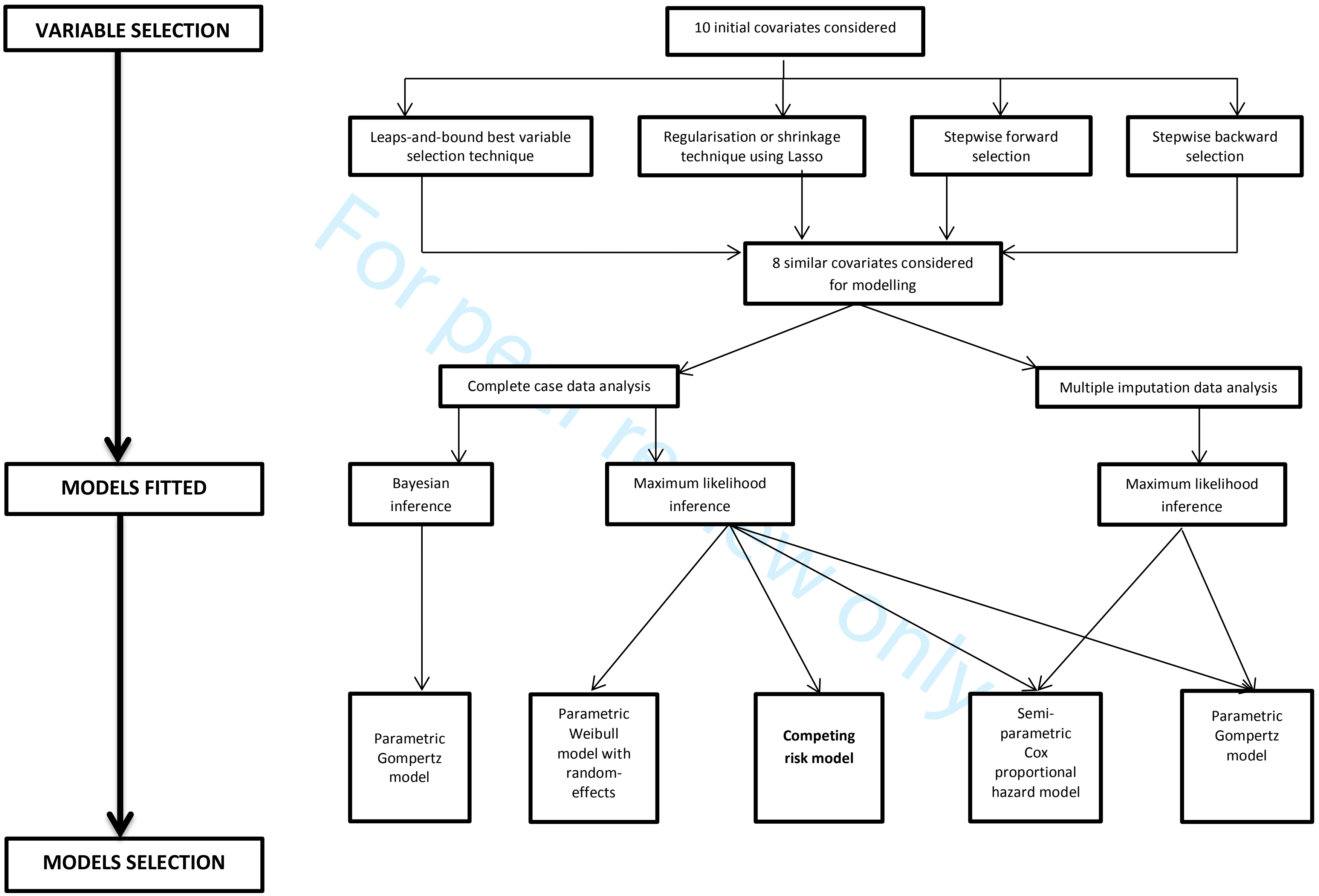

Figure 2: Schematic illustration of variable of importance selection steps and subsequent models fitted for sensitivity analysis of the results

For peer review only - http://bmjopen.bmj.com/site/about/guidelines.xhtml

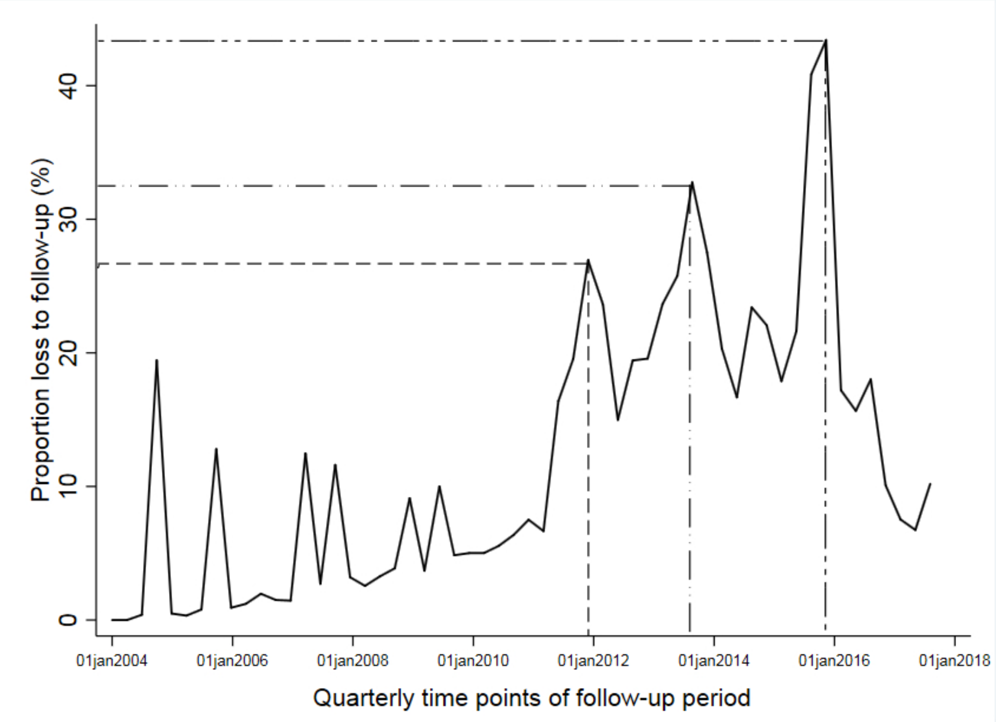

Figure 3: Trend analysis of loss to follow-up outcome among patients receiving ART in Zimbabwe National ART Programme, 2004 to 2017

298x217mm (72 x 72 DPI)

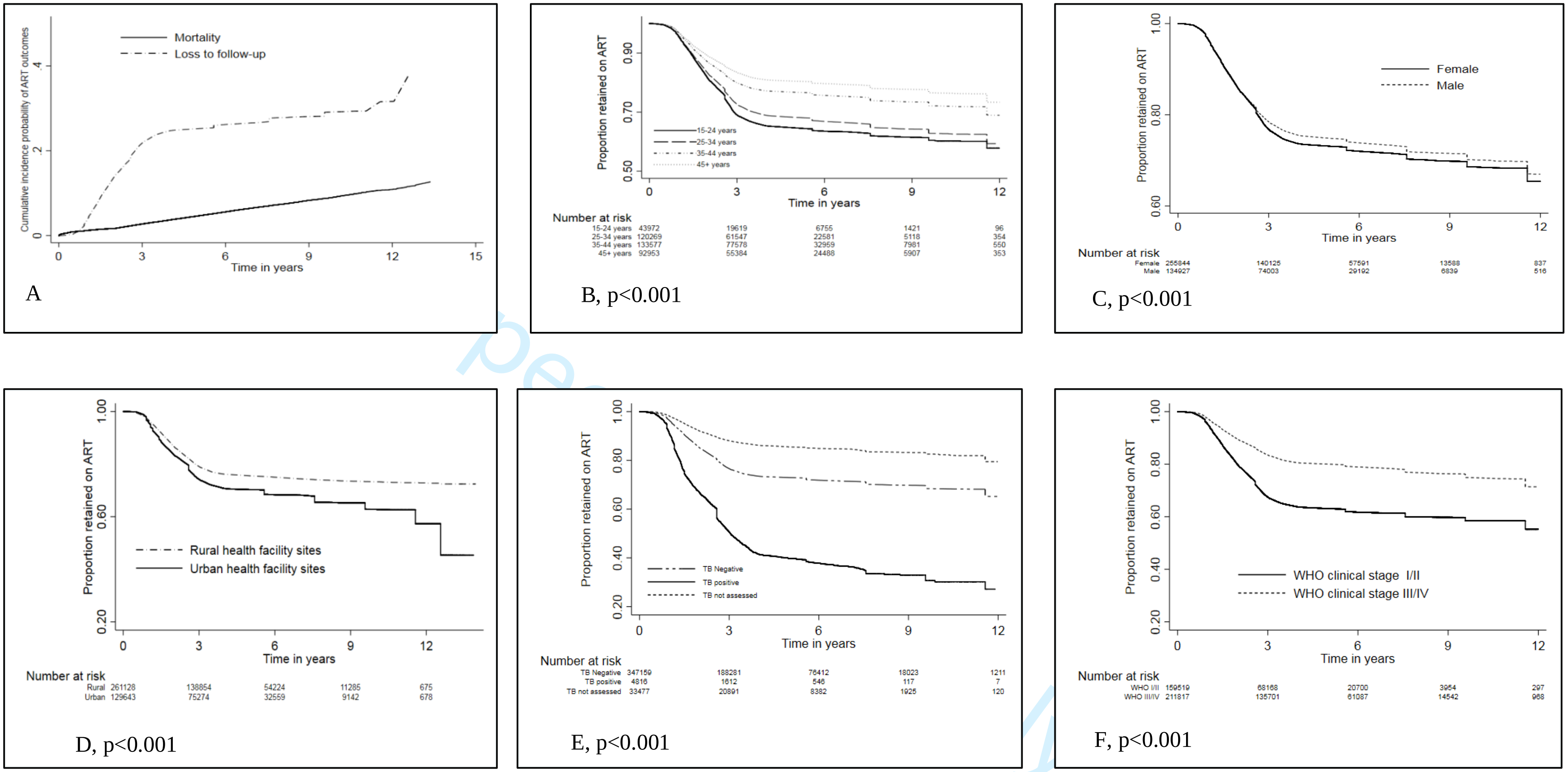

Figure 4: The cumulative incidence probability of mortality and loss to follow-up (A); and Kaplan-Meier curves for loss to follow-up stratified by baseline age categories (B), gender (C), level of urbanization of the health facilities (D), baseline tuberculosis infection status (E) and baseline World Health Organization clinical stages for ART patients from the Zimbabwe national ART Programme electronic patient monitoring data, 2004 to 2017

A1: Cox proportional hazard

Traditionally for time-to-event analysis, researchers tend to fit the semi-parametric cause-specific proportional hazard model which was proposed by Cox (1972) because of its less stringent assumption on the hazard form and survival function [22]. For the conventional cause-specific proportion hazard, we did a non-informative censoring of LTFU and transfer-out. We assumed that the hazard function is an arbitrary, unspecified, non-negative function of time [22].

A2: Survival Function

Let T be a non-negative random variable defining the survival time of an individual. The survival function [$S(t)$] is used to represent the probability that an individual survives from time origin to some time beyond t

$$S(t) = P(T \geq t) \qquad (1)$$

If we assume that T is a continuous random variable $[P(T \geq t) = P(T > t)]$, the different values that T can take have an underlying probability density function (pdf) given by:

$$S(t) = P(T > t) = \int_0^t f(x)dx = 1 - F(t) \qquad (2)$$

where $F(t)$ is the cumulative distribution function (CDF) expressed as:

$$F(t) = P(T < t) = \int_0^t f(x)dx \qquad (3)$$

If one has a survival function, the density function can be calculated by differentiating the cumulative distribution function as:

$$f(t) = -\frac{dF(t)}{dt} \qquad (4)$$

A3: The Hazard function

The hazard function is the probability that an individual dies at time t conditional to having survived to that time. The hazard function, therefore, represents an instantaneous death (failure) rate for an individual who survived to that time. In other words, the hazard function considers the probability that a random variable is associated with an individual's survival time lies between t and $t + \delta t$ conditional to T being greater than or equal to t:

$$h(t) = P(t < T < t + \delta t \mid T \geq t) \\ = \frac{F(t+\delta t) - F(t)}{S(t)} \quad (5)$$

where *F(t)* is the cumulative distribution function. The hazard function is the limiting value of the above probability in Equation 5 divided by the time interval δt, as δt tends to zero. This can be re-written as:

$$\begin{aligned} h(t) = \mu(t) &= \lim_{\delta t \to 0} \frac{P[t \leq T < t + \delta t \mid T \geq t]}{\delta t} \\ &= \lim_{\delta t \to 0} \frac{P[t \leq T < t + \delta t]}{\delta t P(T > t)} \\ &= \lim_{\delta t \to 0} \frac{F[t + \delta t - F(t)]}{\delta t S(t)} \\ &= \frac{1}{S(t)} \left\{ \lim_{\delta t \to 0} \frac{F[t + \delta t - F(t)]}{\delta t} \right\} \end{aligned} \quad . \quad (6)$$

But $\left\{ \lim_{\delta t \to 0} \frac{F[t + \delta t - F(t)]}{\delta t} \right\} = f(t)$ and since T is a continuous random variable, then

$$h(t) = \frac{f(t)}{S(t)} = -\frac{d}{dt} \log[S(t)] \quad . \quad (7)$$

The cumulative hazard is

$$H(t) = \int_0^t h(u)du = -\log[S(x)] \quad . \quad (8)$$

Thus for a continuous lifetimes

$$\begin{aligned} S(t) &= \exp\{-H(t)\} \\ &= \exp\left[-\int_0^t h(u)du \right] \end{aligned} \quad . \quad (9)$$

A4: The Cox regression model

This model is also known as the Proportional hazard regression model, which models the incidence or hazard rate (mortality rate in our case). This model assumes independence of

survival times between distinct individuals in a sample and a constant hazard ratio regardless of time. The model can be written as:

$$\log\left(\frac{h(t)}{h_0(t)}\right) = \beta_1 X_1 + \beta_2 X_2 + \ldots + \beta_k X_k$$
$$\left(\frac{h(t)}{h_0(t)}\right) = \exp(\beta_1 X_1 + \beta_2 X_2 + \ldots + \beta_k X_k) \quad (10)$$
$$h(t) = h_0(t)\exp(\beta_1 X_1 + \beta_2 X_2 + \ldots + \beta_k X_k)$$

This model can be simplified in a matric form as:

$$h(t / \mathbf{Z}) = h_0(t)\exp(\boldsymbol{\beta}\,\mathbf{Z}^T)$$

where $h(t / \mathbf{Z})$ is the expected hazard at time t conditional to the covariate matrix $\mathbf{Z}$. The expected hazard is a product of the baseline hazard ($h_0(t)$) (which is a function of duration time only) and the exponential function of the regressors ($\boldsymbol{\beta} = (\beta_1, \beta_2, \ldots, \beta_k)$; a 1 x k parameter vector) for k covariates. Then the explanatory variables for n persons, $\mathbf{Z}$ matrix, will be:

$$\mathbf{Z} = \begin{bmatrix} X_{11}, & X_{12}, & . & . & ., & X_{1n} \\ X_{21}, & X_{22}, & . & . & ., & X_{2n} \\ . & . & . & . & . & . \\ . & . & . & . & . & . \\ . & . & . & . & . & . \\ X_{k1}, & X_{k2}, & . & . & ., & X_{kn} \end{bmatrix}$$

Thus for the individual covariates, $Z_i = (X_{i1}, \ldots., X_{ik})^T$, the baseline hazard ($h_0(t)$ is equal to *h(t)* when all the regressors are equal to zero, the function of the regressors will be equal to 1. The Cox model is termed semi-parametric because it is made up of the parametric part for the covariates adjustment and the non-parametric baseline hazard as there are no assumptions for the underlying hazard function.

A5: Likelihood function of the Cox regression

The Cox model parameters are estimated by maximising the partial log-likelihood function. Letting the minimum censoring time C_j and survival time T_j be denoted by $t_{(j)}$. The censoring

For peer review only

time is right censored at C after observing that $T_j > C_j$. Let $R(t_j)$ denotes the set of lives which are at risk before the *j*th observed lifetime. Then the partial likelihood is a product of the event times of a quotient that compares the hazard of individuals with the event at a time $t_{(j)}$ to all the hazard of all individuals at risk at a time $t_{(j)}$ can be derived in terms of these censoring times. If we observe $\left\{\left(T_j, \delta_j\right)\right\}_{j=1}^{n}$ where T_j is still the observed time and δ_j the censoring indicator taking values 1 if the failure time is observed for individuals $j = 1, 2, ..., n$ where n is the total number of observations and 0 if right censoring, then the likelihood can be easily expressed as a product of the pdf and the cumulative survival function expressed as $LH \propto \prod_{j=1}^{n} f\left(t_j\right)^{\delta_j} S\left(t_j +\right)^{1-\delta_j}$. This function can be expressed as well as a partial likelihood function in terms of covariates and regression coefficients matrices, as shown below:

$$L_p(\beta \mid D_{obs}) = \prod_{j=1}^{n} \left[\frac{\exp(\boldsymbol{Z}_j^T \boldsymbol{\beta})}{\sum_{k \in R(t_j)} \exp(\boldsymbol{Z}_k^T \boldsymbol{\beta})} \right]^{\delta_j}, \qquad (11)$$

where $D_{obs} = \{(t_j, \delta_j, \boldsymbol{Z}) : j = 1, 2, ..., n\}$ is the observed univariate right-censored survival data. Considering the Breslow concept when ties are present, the partial likelihood may be expressed as:

$$L_{pt}(\beta \mid D_{obs}) = \prod_{j=1}^{d} \frac{\exp(\boldsymbol{Z}_j^T \boldsymbol{\beta})}{\left[\sum_{k \in R(t_j)} \exp(\boldsymbol{Z}_k^T \boldsymbol{\beta}) \right]^{d_j}},$$

where $d = \sum_{j=i}^{n} \delta_j, \boldsymbol{z}_j = \sum_{k \in D_j} \boldsymbol{x}_j, d_j =$ the number of events at $t_{(j)}$ and $R(t_j)$ is a set of all individuals who have the event at the defined time $t_{(j)}$. Thus, this can be written as

$$L_{pt}(\beta \mid D_{obs}) = \prod_{j=1}^{n} \frac{\exp(\delta_j \boldsymbol{Z}_j^T \boldsymbol{\beta})}{\left[\sum_{k \in R(t_j)} \exp(\boldsymbol{Z}_k^T \boldsymbol{\beta}) \right]^{\delta_j}},$$

Expanding the Breslow equation gives:

$$\log L(\beta \mid D_{obs}) = \sum_{j=1}^{D}\left[\sum_{i\in D_j}^{n} \boldsymbol{Z}_j^T \boldsymbol{\beta} - d_j \log\left\{\sum_{k\in R_j}^{n} \exp(\boldsymbol{Z}_j^T \boldsymbol{\beta})\right\}\right] \quad (12)$$

where j indexes the ordered failure times $t_{(j)}, j = 1,\ldots,D$; D_j is the set of d_j observations that fail at time $t_{(j)}$; d_j is the number of failures at time $t_{(j)}$; and R_j is the set of observations k that are at risk at time $t_{(j)}$(that is, all k such that $t_{0k} < t_{(j)} \le t_k$). This can be simplified in set notation form as $k \in R_j$ and $d_j \in D_j$.

A6: Competing risk model specification [49]

In epidemiological studies, more than one cause of failure is possible, hence the estimates of the targeted outcome probabilities are either over-estimated with the Cox model [50] the drive for the competing risk analysis to get marginal probabilities of mortality as proposed by Fine and Gray [17]. Competing risk is when we have more than one cause of failure outcomes, and we are only interested in one of them. In this case, mortality was the competing event to LTFU. The Kaplan-Meier estimate is normally used, where the competing risk events are treated as censored observations, but this is biased since the independence assumption is violated. An individual who is censored because of the competing event will certainly not experience the outcome of interest; hence, the Kaplan-Meier may overestimate the probability of failure, especially if completion is high. Hence, using the Kaplan-Meier estimate in this situation would result in the biased estimates. The competing risk inference is the joint distribution of time of death or failure (T) and failure outcome, in our case death (D) conditional to some covariates, X. The cause-specific hazard (q cause of death) function will be

$$h_q(t) = \lim_{\delta x \to 0} \frac{P(t \le T < t + \delta t, D = q \mid T \ge t)}{\delta t} \quad (13)$$

The cumulative cause-specific hazard is written as:

$$H_q(t) = \int_0^t h_q(u)du \quad (14)$$

And define

$$S_q(t) = \exp(H_q(t)) \quad (15)$$

For peer review only

The cumulative incidence function (CIF) or the sub-distribution function for death is defined as:

$$I_q(t) = P(T \le t, D = q) = \int_0^t h_q(u)S(u)du \qquad (16)$$

This function is sub-distribution since the cumulative probability of death is always below one, that is, $I_q(\infty) = P(D = q) < 1$. The hazard specification would be $h_q(t) = \frac{d\log(1 - I_q(t))}{dt}$, and the hazard model is expressed as

$$h_q(t / \mathbf{Z}) = h_{q,0}(t)\exp(\boldsymbol{\beta}_q \mathbf{Z}^T) \qquad (17)$$

where $h_{q,0}$ is the baseline sub-distribution hazard of cause q and the vector $\boldsymbol{\beta}_q$ represents the matrix of covariates effects of cause q . To estimate the unknown $\boldsymbol{\beta}_q$ parameters, we maximise the following log-pseudolikelihood function:

$$\log L(\beta \mid D_{obs}) = \sum_{i=1}^{n} \delta_i w_i \left[\mathbf{Z}_i^T \boldsymbol{\beta} + offset_i - \log\left\{ \sum_{k \in R_i}^{n} w_i \alpha_{ik} \exp(\mathbf{Z}_k^T \boldsymbol{\beta} + offset_k) \right\} \right] \qquad (18)$$

where δ_i is death for an ith individual, R_i is the set of observations, q at risk at time t_i. The only difference with the Cox is the α_{iq} weights. These weights keep individuals who experience the competing event to be in subsequent risk sets and to reduce their weight over time hence their likelihood of being censored increases.

For peer review only

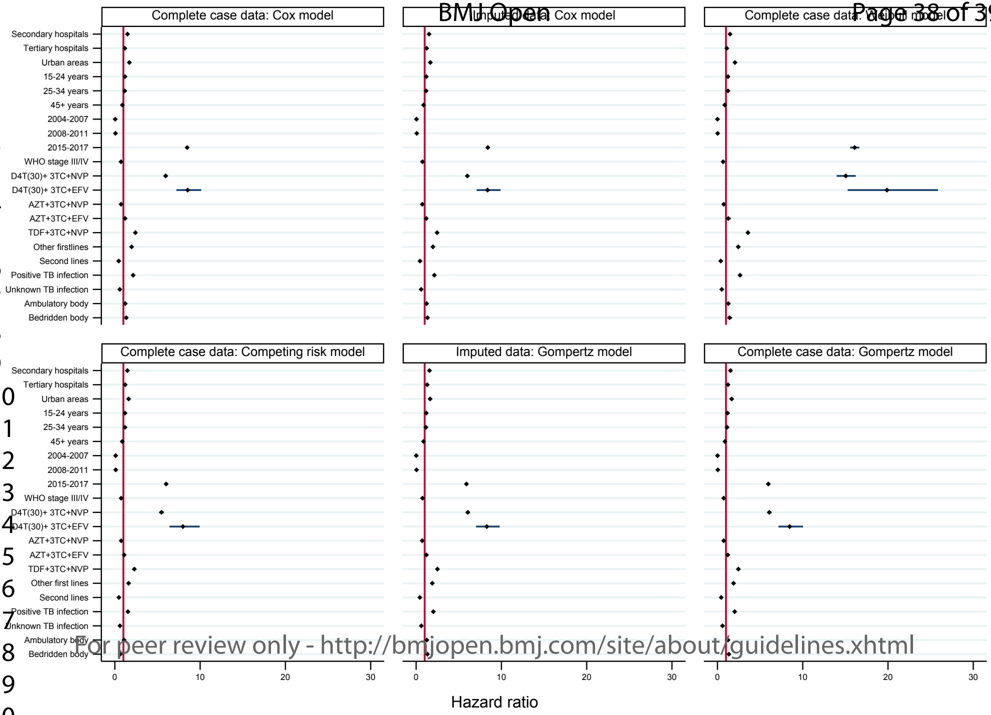

For peer review only - http://bmjopen.bmj.com/site/about/guidelines.xhtml

STROBE 2007 (v4) Statement—Checklist of items that should be included in reports of *cohort studies*

Section/Topic	Item #	Recommendation	Reported on page #
**Title and abstract**	1	(*a*) Indicate the study's design with a commonly used term in the title or the abstract	1
		(*b*) Provide in the abstract an informative and balanced summary of what was done and what was found	1-2
**Introduction**			
Background/rationale	2	Explain the scientific background and rationale for the investigation being reported	3
Objectives	3	State specific objectives, including any prespecified hypotheses	4
**Methods**			
Study design	4	Present key elements of study design early in the paper	4
Setting	5	Describe the setting, locations, and relevant dates, including periods of recruitment, exposure, follow-up, and data collection	4-5
Participants	6	(*a*) Give the eligibility criteria, and the sources and methods of selection of participants. Describe methods of follow-up	4
		(*b*) For matched studies, give matching criteria and number of exposed and unexposed	n/a
Variables	7	Clearly define all outcomes, exposures, predictors, potential confounders, and effect modifiers. Give diagnostic criteria, if applicable	5
Data sources/ measurement	8*	For each variable of interest, give sources of data and details of methods of assessment (measurement). Describe comparability of assessment methods if there is more than one group	
Bias	9	Describe any efforts to address potential sources of bias	5
Study size	10	Explain how the study size was arrived at	4
Quantitative variables	11	Explain how quantitative variables were handled in the analyses. If applicable, describe which groupings were chosen and why	4
Statistical methods	12	(*a*) Describe all statistical methods, including those used to control for confounding	5
		(*b*) Describe any methods used to examine subgroups and interactions	n/a
		(*c*) Explain how missing data were addressed	6
		(*d*) If applicable, explain how loss to follow-up was addressed	n/a
		(*e*) Describe any sensitivity analyses	6
**Results**			

For peer review only

Participants	13*	(a) Report numbers of individuals at each stage of study—eg numbers potentially eligible, examined for eligibility, confirmed eligible, included in the study, completing follow-up, and analysed	4
		(b) Give reasons for non-participation at each stage	n/a
		(c) Consider use of a flow diagram	4
Descriptive data	14*	(a) Give characteristics of study participants (eg demographic, clinical, social) and information on exposures and potential confounders	7-8
		(b) Indicate number of participants with missing data for each variable of interest	8
		(c) Summarise follow-up time (eg, average and total amount)	7
Outcome data	15*	Report numbers of outcome events or summary measures over time	7
Main results	16	(*a*) Give unadjusted estimates and, if applicable, confounder-adjusted estimates and their precision (eg, 95% confidence interval). Make clear which confounders were adjusted for and why they were included	10
		(*b*) Report category boundaries when continuous variables were categorized	n/a
		(*c*) If relevant, consider translating estimates of relative risk into absolute risk for a meaningful time period	n/a
Other analyses	17	Report other analyses done—eg analyses of subgroups and interactions, and sensitivity analyses	10
**Discussion**			
Key results	18	Summarise key results with reference to study objectives	12
**Limitations**			
Interpretation	20	Give a cautious overall interpretation of results considering objectives, limitations, multiplicity of analyses, results from similar studies, and other relevant evidence	12-15
Generalisability	21	Discuss the generalisability (external validity) of the study results	15
**Other information**			
Funding	22	Give the source of funding and the role of the funders for the present study and, if applicable, for the original study on which the present article is based	15

*Give information separately for cases and controls in case-control studies and, if applicable, for exposed and unexposed groups in cohort and cross-sectional studies.

Note: An Explanation and Elaboration article discusses each checklist item and gives methodological background and published examples of transparent reporting. The STROBE checklist is best used in conjunction with this article (freely available on the Web sites of PLoS Medicine at http://www.plosmedicine.org/, Annals of Internal Medicine at http://www.annals.org/, and Epidemiology at http://www.epidem.com/). Information on the STROBE Initiative is available at www.strobe-statement.org.

For peer review only

BMJ Open

Competing risk of mortality on loss to follow-up outcome among HIV Patients on ART: A retrospective cohort study from the Zimbabwe National ART Programme

Journal:	*BMJ Open*
Manuscript ID	bmjopen-2019-036136.R2
Article Type:	Original research
Date Submitted by the Author:	24-Aug-2020
Complete List of Authors:	Matsena Zingoni, Zvifadzo; University of the Witwatersrand Faculty of Health Sciences, Division of Epidemiology and Biostatistcs; Ministry of Health and Child Care, National Institute of Health Research<br>Chirwa, Tobias; University of the Witwatersrand Faculty of Health Sciences, Division of Epidemiology and Biostatistics<br>Todd, Jim; LSHTM, EPH<br>Musenge, Eustasius; University of the Witwatersrand Faculty of Health Sciences, Division of Epidemiology and Biostatistics
**Primary Subject Heading**:	HIV/AIDS
Secondary Subject Heading:	Public health, Epidemiology, Health services research, Infectious diseases, Health policy
Keywords:	Epidemiology < INFECTIOUS DISEASES, HIV & AIDS < INFECTIOUS DISEASES, Public health < INFECTIOUS DISEASES

SCHOLARONE™
Manuscripts

I, the Submitting Author has the right to grant and does grant on behalf of all authors of the Work (as defined in the below author licence), an exclusive licence and/or a non-exclusive licence for contributions from authors who are: i) UK Crown employees; ii) where BMJ has agreed a CC-BY licence shall apply, and/or iii) in accordance with the terms applicable for US Federal Government officers or employees acting as part of their official duties; on a worldwide, perpetual, irrevocable, royalty-free basis to BMJ Publishing Group Ltd ("BMJ") its licensees and where the relevant Journal is co-owned by BMJ to the co-owners of the Journal, to publish the Work in this journal and any other BMJ products and to exploit all rights, as set out in our licence.

The Submitting Author accepts and understands that any supply made under these terms is made by BMJ to the Submitting Author unless you are acting as an employee on behalf of your employer or a postgraduate student of an affiliated institution which is paying any applicable article publishing charge ("APC") for Open Access articles. Where the Submitting Author wishes to make the Work available on an Open Access basis (and intends to pay the relevant APC), the terms of reuse of such Open Access shall be governed by a Creative Commons licence – details of these licences and which Creative Commons licence will apply to this Work are set out in our licence referred to above.

Other than as permitted in any relevant BMJ Author's Self Archiving Policies, I confirm this Work has not been accepted for publication elsewhere, is not being considered for publication elsewhere and does not duplicate material already published. I confirm all authors consent to publication of this Work and authorise the granting of this licence.

For peer review only

Competing risk of mortality on loss to follow-up outcome among HIV Patients on ART: A retrospective cohort study from the Zimbabwe National ART Programme

Zvifadzo Matsena Zingoni[§, 1, 2], Tobias Freeman Chirwa[1], Jim Todd[3] and Eustasius Musenge[1]

[1]Division of Epidemiology and Biostatistics, University of the Witwatersrand, Johannesburg, South Africa

[2]National Institute of Health Research, Ministry of Health and Child Care, Harare, Zimbabwe

[3]Department of Population Health, London School of Hygiene and Tropical Medicine, London, United Kingdom

[§]Corresponding author: Zvifadzo Matsena Zingoni (ZMZ)
27 St Andrews' Road
Parktown, 2193 Johannesburg, South Africa
email: zmatsena28@gmail.com
ORCID number: 0000-0002-7993-1187

Authors email addresses of authors:
Tobias Freeman Chirwa (TFC): Tobias.Chirwa@wits.ac.za
Jim Todd (JT): jim.todd@lshtm.ac.uk
Eustasius Musenge (EM): Eustasius.Musenge@wits.ac.za

Word count

Abstract: 299
Main body: 3975

ABSTRACT

Objective: To determine the loss to follow-up (LTFU) rates at different health care levels after ART services decentralisation among ART patients who initiated ART between 2004 and 2017 using the competing risk model in addition to the Kaplan-Meier and Cox regressions analysis.

Design: A retrospective cohort study

Setting: The study was done in Zimbabwe using a nationwide routinely collected HIV patient-level data from various health levels of care facilities compiled through the electronic patient management system (ePMS).

Participants: We analysed 390,771 participants aged 15years and above from 538 health facilities.

For peer review only

Outcomes: The primary endpoint was LTFU defined as a failure of a patient to report for drug refill for at least 90 days from last appointment date or if the patient missed the next scheduled visit date and never showed up again. Mortality was considered a secondary outcome if a patient was reported to have died.

Results: The total exposure time contributed was 1,544,468 person-years. LTFU rate was 5.75 (95% confidence interval (CI): 5.71-5.78) per 100 person-years. Adjustment for the competing event independently increased LTFU rate ratio in provincial and referral (adjusted sub-hazard ratios (AsHR) 1.24; 95%CI: 1.20-1.28) and district and mission (AsHR 1.47; 95%CI: 1.45-1.45) hospitals (reference: primary health care); in urban sites (AsHR 1.58; 95%CI: 1.55-1.60) (reference: rural); and among adolescence and young adults (15-24years) group (AsHR 1.17; 95%CI: 1.15-1.19) (reference: 35-44years). We also detected overwhelming association between LTFU and tuberculosis infected patients (AsHR 1.55; 95%CI: 1.47-1.64) (reference: no tuberculosis).

Conclusions: We have observed considerable findings that "leakages" (LTFU) within the ART treatment cascade persist even after the decentralisation of health services. Risk factors for LTFU reflect those found in sub-Saharan Africa studies. Interventions which retain patients in care by minimising any "leakages" along the treatment cascade are essential in attaining the UNAIDS 90-90-90 fast track targets.

Keywords: Antiretroviral therapy, Competing risk analysis, Loss to follow-up, Mortality, Retention, Risk factors,

Article Summary: Strength and limitations of the study

- The analysis is based on a huge national routinely collected individual-level dataset which has a big follow-up period making our findings generalisable to the HIV patients on ART in Zimbabwe.
- This study fitted a competing risk model based on the cumulative incidence function (CIF) to explore the association between selected covariates and the absolute risk which is an essential modelling approach for medical decision-making so that appropriate sub-population intervention can be implemented.

For peer review only

For peer review only - http://bmjopen.bmj.com/site/about/guidelines.xhtml

- Variable selection of independent factors was performed using the regularisation (shrinkage) lasso regression technique alongside the stepwise section methods.
- Important factors known to influence LTFU like CD4 cell counts and viral load were not accounted for in the model due to the high percentage of missingness, and the independent factors adjusted for were not time-dependent.
- This study has a possibility of participant selection bias since only participants enrolled in health facilities linked to the electronic patients' management database were conveniently sampled for analysis.

INTRODUCTION

Globally, remarkable progress has been made on the treatment of HIV-infected people using antiretroviral therapy(ART), particularly in sub-Saharan Africa(SSA)[1]. In Zimbabwe, the ART programme is a comprehensive care and support package aiming to slow disease progression[2], improve survival and quality of life [3]; and minimise HIV transmission by reducing the viral load. Therefore, it is imperative to monitor individuals on ART to ensure treatment efficacy, support adherence; and identify treatment failure and resistance.

At the national level, aggregated health facility data is usually used for monitoring the ART programme. However, there is an inherent difficulty in using aggregated data to make statistical inferences about individual-level outcomes. This promoted the increasing use of patient-level data worldwide to assess the impact of the ART programme [4–8]. With efforts to achieve zero HIV incidences by 2030, the 90-90-90 UNAIDS targets were launched, whereby 90% of the people living with HIV (PLWHIV) should know their status, 90% of those diagnosed should be initiated on ART, and 90% of those on ART should achieve viral suppression[9]. In Zimbabwe, strides have been made towards achieving these targets; however, limitations exist. For instance, in Zimbabwe, only 73% of PLWHIV who are on ART have achieved viral suppression [10]. To improve the proportion of those who achieve viral suppression, it is crucial to come up with broad efforts which minimise long-term ART attrition due to loss to follow-up (LTFU), mortality, drop-outs and withdrawals.

In Zimbabwe, the health service delivery can be categorised into four levels of care, namely the primary, secondary, tertiary and quaternary[11]. The primary care consists of clinics and

For peer review only - http://bmjopen.bmj.com/site/about/guidelines.xhtml

facilities offering basic preventive and curative services. Majority of these primary health care facilities are in the rural areas, and health problems beyond the scope of these facilities are referred to the secondary health care centres, which are mainly the district and mission hospitals. In the secondary care category, private and company health facilities may fit in if they can handle emergency services as the district hospitals. The tertiary care consists of the provincial hospitals which received referral patients from the district hospitals[12]. The quaternary care is the last referral care level which received patients from provincial hospitals. These facilities are mainly in the two largest cities in Zimbabwe[12]. The quaternary health care offers much more advanced health care; hence, there is modern equipment, medical specialists and pharmaceutical, which could be missing in the lower levels of care[11].

The ART services were initially centralised at the higher levels of care – quaternary and tertiary health care facilities- normally situated in urban areas. This means rural residents and poorer people were less likely to access HIV testing, care and treatment due to transport cost. In 2010/11, one of the strategies to mitigate the transport barrier was the decentralisation of ART services to lower levels of care [13]. These devolved care models were implemented in Zimbabwe to improve patients' access to HIV care, ART adherence and retention in care of PLWHIV. The impact of HIV care and ART decentralisation has been well documented in the SSA region[14]. Understanding ART retention patterns after the implementation of decentralised care is vital to guide the ART programme and policy as well as reflecting on the gaps which may contribute to the poor viral suppression. Loss to follow up (LTFU) is one of the main threats to patient retention and may persist even after the decentralisation of ART services This may be due to social (stigmatization, social deprivation and health literacy issues) and individual factors (perception and misconceptions over ART benefits) which still hinder retention in care [15].

This study aims to determine LTFU rates at different health care levels after ART services decentralisation among ART patients who initiated ART between 2004 and 2017. The study period covers the pre- and post-ART decentralisation period to get an overall picture of the LTFU patterns over time. The LTFU rates were compared between the different health care facility types (primary health care (PHC), district/mission and provincial/referral hospitals) in Zimbabwe using routinely collected patients-level data from the Zimbabwe National ART database. We further adjusted for the individual-level demographic and clinical characteristics as

For peer review only - http://bmjopen.bmj.com/site/about/guidelines.xhtml

predisposing or independent factors for LTFU using a statistical model that adjusted for the competing risk of mortality on the LTFU outcome.

METHODS

Study Population

This study was conducted in Zimbabwe, a landlocked country in SSA with roughly 1.4 million PLWHIV. The HIV prevalence dropped from 25% in 2004 to 13.7% in 2017 among adults [10,16]. Individual patients aged 15 years and above, who initiated ART between 1 January 2004 and 31 December 2017, were eligible for this analysis. A full description of how the final sample size was achieved is illustrated in Figure 1.

[Insert Figure 1]

Study design and Data sources

This study was a secondary data analysis from a retrospective cohort study of routinely collected patient data from the Zimbabwe nation ART programme. The data came from the national ART programme, which started in 2004. The ART sites expanded from 5 health facilities in 2004 to 1,566 by 2017[10,17]. Treatment guidelines followed WHO recommendations; hence, the ART eligibility criteria and treatment regimens changed over time. The initial regimen for an ART naïve patient is the first-line ART, a combination of two nucleoside reverse transcriptase inhibitors(NRTI), the first drug which can be Zidovudine (AZT), Tenofovir (TDF) (the most common) or Stavudine (D4T) and the second drug is mainly Lamivudine (3TC); and one non-NRTI which can either be Nevirapine (NVP) or Efavirenz (EFV). Majority of the participants were receiving three-drug combination therapy of TDF+3TC+EFV therapy as the first-line treatment. Once the patient experienced treatment failure or intolerance, switch to second-line ART (a three-drug combination of protease inhibitor and two NRTIs) can be made[18].

Monitoring and Evaluation System

The monitoring and evaluation system of the ART programme in Zimbabwe has been described elsewhere[19]. Briefly, since the inception of ART, all patient monitoring records (PMR) had been collected manually using a paper-based system and kept at the health facility where the patient is registered. Since 2012 the paper-based system was no longer functioning properly due

For peer review only - http://bmjopen.bmj.com/site/about/guidelines.xhtml

to high volumes of ART patients [10]. This upsurge in patients' volumes began to affect the accurate ART monitoring and reporting process as the paper-based system could not cope. Moreover, the paper-based system translated into huge workloads and became strenuous to the already over-burdened health workers. As a result, the electronic patient management system (ePMS) was launched in 2012. The ePMS increased efficiency and effective management of ART patients through improving follow-ups to subsequently increase ART adherence[19]. The ePMS provided a platform to compile quality patient-level data to accurately make forecast and evaluate the ART intervention, enabling a more effective program [10].

The ePMS was rolled out in systematic phased approach with priority given to those HIV sites with high volumes of HIV patients at the same time maintaining a good representation of the four health care levels in the country [19]. From the ePMS Strategic Plan document, the first phase target was 83 sites in 2013 comprising of central, provincial and district hospitals which covered approximately 61% of ART patients nationwide. Additional 267 city polyclinics, rural/large mission hospitals sites were included to make a total of 350 sites by the end of 2014[19]. The last phase included 184 static ART follow up sites to bring the total to 534 sites by the end of 2017. By the end of 2018, there were 622 sites with a functional ePMS with the inclusion of private hospitals which meant that approximately 95% of all patients receiving ART were linked to the ePMS database [10].

The final database of the ePMS has patients' information from all levels of care (quaternary, tertiary, secondary and primary health care levels). It, therefore, reflects the overall ART management services in the country, although the database includes all HIV-related data. At enrolment, the patient's demographic data are captured. At each subsequent clinic visit, information on ART regimen type, clinical information, laboratory investigations and physical examination is captured. Outcomes are assessed and captured as death, LTFU, transfer-out to another health facility or alive and active on treatment. Already existing patient's information prior to 2012 from the paper-based system was retrospectively transcribed into the electronic database at each clinic, and all subsequent clinic visits were recorded prospectively with a real-time entry of patient records.

The introduction of the ePMS for monitoring HIV patients in 2012 has provided a platform for huge data repository in the country which may be used to answer some of the public health

For peer review only - http://bmjopen.bmj.com/site/about/guidelines.xhtml

questions on the HIV aspects using statistical models. Moreover, the ePMS platform provides readily available data archiving for robust statistical analysis approaches to guide evidence-based decision-making and programme interventions planning at patients-level while eliminating the aggregated data. Therefore, this rich, huge patient-level database was of preference and suitable to answer our study objective.

Study Endpoints

The primary endpoint was LTFU defined as a failure of a patient to report for drug replenishment within 90 days from last appointment date or if the patient missed the next scheduled visit date and never showed up again. Patients, who returned after having been LTFU, were classified as alive and active on treatment. We also consider the mortality outcome as a competing event of LTFU if a patient was reported to have died.

Data Analysis Methods

Descriptive summaries were reported as frequencies and proportions stratified by health facility type. The pattern of LTFU was explored by calendar year. Incidence rates were calculated from the ART initiation date to outcome occurrence date on a quarterly time scale. Kaplan-Meier non-parametric curves (Equations 1-4 in Appendix) were used to describe the probability of LTFU over time stratified by selected covariates. The log-rank test was used to assess the equality of these patient retention probabilities. Estimated hazard rates (HR) (Equation 5-9 in Appendix) were reported as per 100 person-years.

Important variable selection was done using the lasso regression regularisation technique. Variable selection sensitivity analysis was done using the subset selection techniques (best subset selection, stepwise forward variable selection and stepwise backward variable selection methods). All these methods yield the same independent factors considered in the seven different multiple adjusted time-to-event models fitted, Figure 2. The baseline predictors used for adjustment, were all categorical, namely: health facility type, degree of urbanisation of the health facility (rural/urban), age groups (15-24, 25-34, 35-44, 45 and above), tuberculosis status (positive/ negative), body functional status-which is the patient's ability to perform normal daily activities expected for the well-being of an individual-(working body, ambulatory body, bed-ridden body), WHO clinical stages (stage 1, II, III and IV), ART enrolment calendar period

For peer review only - http://bmjopen.bmj.com/site/about/guidelines.xhtml

(2004-2007; 2008-2011; 2012-2014; 2015-2017) and regimen type (Stavudine, Zidovudine or Tenofovir based combination therapy). The stratification of the health facility type into primary health care, district/mission hospitals, and provincial/referral hospitals was based on the level of care classification of the corresponding health facility an individual was enrolled in, and the grouping of the health facilities was based on the patients' care similarities. For those individuals who had moved from one health facility to the other during the study period covered, the last recorded health facility site was considered for this analysis. Therefore, the health level of care classification was mutually exclusive as one individual was assigned to the last captured health facility.

[Insert Figure 2]

The standard Cox regression model defined in Equation 10-12 in the Appendix is normally used for a time to event analysis; however, this method may give biased estimates in the presence of competing events. We fitted the competing risk (CR) model of mortality on LTFU through the cumulative incidence function (CIF) define in Equation 13-18 in Appendix) [20]. CR analysis assumes that patients are exposed to at least two risks (mortality and LTFU). One of the events is of interest (LTFU) while the other is a competing event (mortality) which inhibits the outcome of interest from occurring. The competing events may not be independent, and the baseline hazard may differ between them. The covariates used were time-independent; however, we adjusted for time to take into account the period the country experienced an economic turmoil around 2008 which resulted in a mass withdrawal of health personnel in the public sectors and an increased scarcity of vital medical supplies in health system including HIV medication [21,22]. The adjusted sub-hazard ratios (AsHR) for covariates on LTFU were reported with 95% confidence intervals (95% CI). We censored on 31 December 2017 for those who had not experienced the event of interest and at the last observed date for those who had transferred to other health facilities.

We performed a sensitivity analysis of our key findings through multiple imputations of the missing explanatory covariates using Rubin's method, which assumes that the data are missing at random (MAR) [23]. We compared imputed data and the complete case data estimates based on the maximum likelihood estimation (MLE) perspective for both the Cox proportional hazard and Gombertz parametric models. Within the Gombertz parametric models, estimates from the

For peer review only - http://bmjopen.bmj.com/site/about/guidelines.xhtml

Bayesian estimation (BE) and MLE were compared [24]. We assumed non-informative normal priors for all unknown parameters to be estimated with a mean of zero and variance of 100 or precision of 0.001 for the parametric Bayesian model. We fitted a parametric Weibull model with random effects to account for any individual-heterogeneity[25]. Model comparison between BE and MLE was done using the Akaike's information criterion (AIC), Bayesian information criterion (BIC) and Deviance information criterion (DIC). In contrast, model comparison between complete case analysis and imputed data analysis was done using the precision of the confidence interval for the estimates. All data management and analysis were performed using Stata 15.1 (StataCorp, 2017, Stata Statistical Software: Release 15.1, College Station, TX: StataCorp LLC)[26].

Patient and Public involvement

This study acknowledges the importance of patients and public involvement in research studies; however, this research was done without patient involvement in all research stages since it was secondary data analysis.

Ethical Approval

Ethics approval for this study was granted by The University of Witwatersrand's Human Research Ethics Committee (Medical) (Clearance Certificate No. M170673). Data approval was obtained from the Ministry of Health and Child Care in Zimbabwe, who are the custodians of the countrywide database. Since this study was secondary data analysis, we did not have direct contact with the participants. We received only anonymised data for analyses.

RESULTS

Descriptive Analysis

At the end of study 268,896 (68.81%) patients were alive, 18,328(4.7%) had died, 88,744 (22.71%) were LTFU, and 14,803(3.79%) had transferred out from the initial health facility another health facility. Over 1,544,468 person-years of ART, mortality and LTFU rates were 1.19 (95%CI: 1.17-1.20) and 5.75(95%CI: 5.71-5.78) per 100 person-years, respectively. The

For peer review only - http://bmjopen.bmj.com/site/about/guidelines.xhtml

quarterly trend analysis graph of LTFU over time showed a general increase in LTFU over time with significant peaks in 2012, 2014 and 2016 (Figure 3).

[Insert Figure 3]

The descriptive characteristics of 390,771 patients from 538 health facilities are presented in Table 1**.** The study participants were enrolled at PHC (45.1%), districts/mission (49.2%) or provincial/referral (5.7%) hospitals. Most patients were enrolled in rural health facilities (66.82%). At ART initiation, majority of the patients were female 117,567(66.7%) with mean (standard deviation) age of 37.5(10.2) years, and only 6.44% (n=25, 143) patients had either an ambulatory or a bed-ridden body functional status.

Table 1: Patients sociodemographic and clinical characteristics at ART initiation in Zimbabwe national ART programme, 2004-2017

Characteristics	Categories	Primary health care (PHC) n=176 253 (45.1%)	District/Mission hospitals n=192 238(49.2%)	Provincial/Referral hospitals n=22 280 (5.7%)	Totals N=390 771 (100%)
**Patient Demographics**					
Age (years) at ART initiation	15-24	20 314 (11.53%)	21 057 (10.95%)	2 601 (11.67%)	43 972 (11.25%)
	25-34	54 416 (30.87%)	58 874 (30.63%)	6 979 (31.32%)	120 269 (30.78%)
	35-44	59 284 (33.64%)	66 418 (34.55%)	7 875 (35.35%)	133 577 (34.18%)
	45+	42 239 (23.96%)	45 889 (23.87%)	4 825 (21.66%)	92 953 (23.79%)
Degree of urbanisation	Rural	113 738 (66.92%)	137 380 (73.57%)	432 (2.01%)	251 550 (66.52%)
	Urban	56 223 (33.08%)	49 348 (26.43%)	21 055 (97.99%)	126 626 (33.48%)
Sex	Females	117 567 (66.70%)	124 141 (64.58%)	14 136 (63.45%)	255 844 (65.47%)
	Male	58 686 (33.30%)	68 097 (35.42%)	8 144 (36.55%)	134 927 (34.53%)
Education status at ART initiation	None	3 854 (2.19%)	4 136 (2.15%)	227 (1.02%)	8 217 (2.10%)
	Primary	33 735 (19.14%)	34 062 (17.72%)	2 248 (10.09%)	70 045 (17.92%)
	Secondary	59 181(33.58%)	58 349 (30.35)	6 593 (29.59%)	124 123 (31.76%)
	Tertiary	2 921(1.66%)	4 423(2.30%)	1 177(5.28%)	8 521 (2.18%)
	Missing	76 562(43.44%)	91 268 (47.48%)	12 035(54.02)	179 865 (46.03%)
Marital status at ART initiation	Single	24 438 (13.87%)	24 489 (12.74%)	3 530 (15.84%)	52 457 (13.42%)
	Married	105 504 (59.86%)	113 953 (59.28%)	12 935 (58.06%)	232 392 (59.47%)
	Widowed	25 798 (14.64%)	30 994 (16.12%)	3 523 (15.81%)	3 523 (15.81%)
	Divorced	13 429 (7.62%)	14 732 (7.66%)	1 677 (7.53%)	29 838 (7.64%)
	Missing	7 084(4.02%)	8 070 (4.20%)	615 (2.76%)	15 769 (4.04%)
Calendar time of starting ART	2004-2007	6 635 (3.76%)	8 942 (4.65%)	2 962 (13.29%)	18539 (4.74%)
	2008-2011	38 990 (22.12%)	61 321 (31.85%)	7 899 (35.45%)	108120 (27.67%)
	2012-2014	67 759 (38.44%)	90 582 (47.12%)	9 472 (42.51%)	167 813 (42.94%)
	2015-2017	62 869 (35.67%)	31 483 (16.38%)	1 947 (8.74%)	96 299 (24.64%)
**Clinical factors**					
WHO staging at ART initiation	I/II	82 203 (46.64%)	69 629 (36.22%)	7 690 (34.52%)	159 519 (40.82%)
	III/IV	79 279 (44.98%)	118 113 (61.44%)	14 425 (64.74%)	211 817 (54.20%)
	Missing	14 771 (8.38%)	4 499 (2.34%)	165 (0.74%)	19 435 (4.97%)
Baseline CD4 (cell/uL) at ART initiation	Above 500	4 783 (2.71%)	4 950 (2.57%)	601 (2.70%)	10 334 (2.64%)
	351-500	8 227 (4.67%)	8 790 (4.57%)	940 (4.22%)	17 957 (4.60%)
	200-350	17 908 (10.16%)	24 895 (12.95%)	2 866 (12.86%)	45 669 (11.69%)

For peer review only - http://bmjopen.bmj.com/site/about/guidelines.xhtml

	Below 200	23 796 (13.50%)	35 020 (18.22%)	4 982 (22.36%)	63 798 (16.33%)
	Missing	121 539 (68.96%)	118 583 (61.69%)	12 891 (57.86%)	253 013 (64.75%)
Treatment regimen at the time of data extraction	First-line	170 662 (98.29%)	184 321 (97.37%)	20 724 (94.32%)	375 704 (97.65%)
	D4T(30)+3TC+NVP	625 (0.35%)	2 942 (1.53%)	629 (2.82%)	4 196 (1.07%)
	D4T(30)+ 3TC+EFV	42 (0.02%)	155 (0.08%)	20 (0.09)	217(0.06%)
	AZT+3TC+NVP	1 349 (0.77%)	1 933 (1.01%)	349 (1.57%)	3 631(0.93%)
	AZT+3TC+EFT	212 (0.12%)	425 (0.22%)	56 (0.25 %)	693 (0.18%)
	TDF+3TC+NVP	6 541 (3.71%)	16 334 (8.50%)	2 923 (13.12%)	25 798 (6.60%)
	TDF+3TC+EFV	161 288 (91.51%)	162 006 (84.27%)	16 628 (4.63%)	339 922 (86.99%)
	Other first lines	3 236 (1.84%)	3 474 (1.81%)	428 (1.92%)	7 138 (1.83%)
	Second line	2 960 (1.68%)	4 969 (2.58%)	1 247(5.60%)	9 176 (2.35%)
Tuberculosis status at ART initiation	Negative	156 072 (88.55%)	170 869 (88.88%)	20 218 (90.75%)	347 159 (88.84%)
	Positive	2 092 (1.19%)	2 423 (1.26%)	301 (1.35%)	4 816 (1.23%)
	Not assessed	15 743 (8 93%)	16 387 (8.52%)	1 347 (6.05%)	33 477 (8.57%)
	Missing	2 346 (1.33%)	2 559 (1.33%)	414 (1.86%)	5 319 (1.36%)
Body functional status at ART initiation	Working	162 710 (92.32%)	180 417 (93.85%)	20 024 (89.87%)	363 151 (92.93%)
	Ambulatory	11 789 (6.69%)	10 454 (5.44%)	2 089 (9.38%)	24 332 (6.23%)
	Bed-ridden	331 (0.19%)	452 (0.24 %)	28(0.13%)	811 (0.21%)
	Missing	1 423 (0.81%)	915 (0.48%)	139(0.62%)	2 477 (0.63%)

Missing category refers to unavailable information in the dataset for that particular variable

Most patients were married (59.5%) with secondary education (31.8%) while 54.2% were classified in WHO stage III or IV and 34.18% were in the 35-44 years age group. The majority of the participants were on first-line ART treatment (97.7%), and only a few had confirmed tuberculosis infection (1.2%). There were missing data on some covariates, including baseline CD4 cell counts recorded for only 35.3% of the study participants**.**

The time-to-event rates for LTFU stratified by baseline characteristics are shown in Table 2. LTFU rate per 100-person-years was 5.92 for patients enrolled at district and mission hospitals; 6.60 amongst patients from rural health facilities and 17.09 among patients diagnosed with tuberculosis.

Table 2: Rates per 100 person-years of loss to follow-up and log-rank tests for ART patients' baseline characteristics in Zimbabwe national ART programme, 2004-2017

Characteristics	Category	LTFU cases	Person-years	Rate per 100 person-years	95% CI of rate	Log-rank p-value
Health facility level	Primary health care	34 958	6 200	5.6502	5.5913 to 5.7097	
	District/mission hospitals	48 188	8 100	5.9169	5.8643 to 5.9700	<0.001*
	Provincial/referral hospitals	5 598	1 100	5.0273	4.8973 to 5.1608	
Age (years) at ART initiation	15-24	32 790	4 500	7.3252	7.2463 to 7.4049	
	25-34	12 617	1 500	8.4301	8.2843 to 8.5785	
	35-44	27 504	5 500	4.9671	4.9087 to 5.0261	<0.001*
	45+	15 833	3 900	4.0242	3.9620 to 4.0874	
Degree of urbanisation	Rural	52 629	10 000	5.2756	5.2307 to 5.3209	
	Urban	36 115	5 500	6.6039	6.5362 to 6.6724	<0.001*
Sex	Females	59 695	10 000	5.8703	5.8234 to 5.9175	
	Male	29 049	5 300	5.5063	5.4433 to 5.5700	<0.001*

For peer review only - http://bmjopen.bmj.com/site/about/guidelines.xhtml

Education status at ART initiation	None	1 470	354	4.1582	3.9509 to 4.3762	
	Primary	14 786	2 800	5.3558	5.2702 to 5.4429	
	Secondary	32 629	4 700	6.9961	6.9206 to 7.0725	<0.001*
	Tertiary	1 803	409	4.4097	4.2108 to 4.6180	
	Missing	38 056	7 300	5.2435	5.1911 to 5.2965	
Marital status at ART initiation	Single	13 078	1 900	6.9036	6.7863 to 7.0230	
	Married	7 548	1 100	6.6112	6.4637 to 6.7620	
	Widowed	9 375	2 900	3.1872	3.1233 to 3.2524	<0.001*
	Divorced	55 985	8 800	6.3313	6.2790 to 6.3839	
	Missing	2 758	625	4.4160	4.2543 to 4.5840	
Calendar time of starting ART	2004-2007	790	1 900	0.4255	0.3968 to 0.4562	
	2008-2011	4 572	6 900	0.6593	0.6405 to 0.6787	
	2012-2014	43 565	5 300	8.1802	8.1038 to 8.2574	<0.001*
	2015-2017	39 817	1 300	29.9903	29.6972 to 30.2863	
WHO staging at ART initiation	I/II	47 410	5 100	9.2080	9.1255 to 9.2912	
	III/IV	38 919	9 500	4.0957	4.0552 to 4.1366	<0.001*
	Missing	2 415	794	3.0432	2.9242 to 3.1671	
Baseline CD4 (cell/uL) at ART initiation	Above 500	4 341	257	16.9190	16.4231 to 17.4299	
	351-500	7 614	455	16.7362	16.3645 to 17.1164	
	200-350	11 556	1 700	6.7428	6.6210 to 6.8669	<0.001*
	Below 200	15 271	2 700	5.7338	5.6436 to 5.8254	
	Missing	49 962	10 000	4.8245	4.7823 to 4.8669	
Treatment regimen at the time of data extraction	First line	88 050	15 000	5.9066	5.8677 to 5.9457	
	D4T(30)+ 3TC+NVP	2 685	168	15.9847	15.3914 to 16.6009	
	D4T(30)+ 3TC+EFV	141	10	14.7353	12.4932 to 17.3797	
	AZT+3TC+NVP	448	184	2.4374	2.2218 to 2.6738	
	AZT+3TC+EFT	158	30	5.3159	4.5484 to 6.2129	<0.001*
	TDF+3TC+NVP	9 652	1 100	9.1737	8.9925 to 9.3586	
	TDF+3TC+EFV	71 378	13 000	5.3710	5.3318 to 5.4106	
	Other first lines	3 588	175	20.5590	19.8972 to 21.2429	
	Second line	694	538	1.2900	1.1985 to 1.3908	
Tuberculosis status at ART initiation	Negative	81 119	14 000	5.9418	5.9010 to 5.9828	
	Positive	2 235	131	17.0906	16.3966 to 17.8141	
	Not assessed	4 188	1 400	2.9328	2.8453 to 3.0230	<0.001*
	Missing	1 202	234	5.1455	4.8627 to 5.4447	
Body functional status at ART initiation	Working	81 122	14 000	5.6445	5.6057 to 5.6834	
	Ambulatory	7 059	930	7.5897	7.4147 to 7.7688	
	Bed-ridden	215	26	8.4201	7.3666 to 9.6243	<0.001*
	Missing	348	117	2.9723	2.6758 to 3.3015	

*Significant at 5%; [*]Missing category refers to unavailable information in the dataset for that particular variable

The cumulative incidence probability (CIP) curve for death was lower than the CIP for LTFU as the outcome (Figure 4A). The LTFU CIP curve increased in the first 3 years on ART and became steady after that. We observed overwhelmingly significant variation between LTFU and selected covariates, log-rank p-value<0.001. Higher risk of LTFU was observed amongst 15-24 years patients (Figure 4B), females (Figure 4C), patients enrolled at urban health facilities (Figure 4D); tuberculosis-infected patients (Figure 4E) and WHO stage I/II patients (Figure 4F).

[Insert Figure 4]

For peer review only

Loss to Follow-up Outcome

To determine the possible independent risk factors associated with LTFU, seven models were fitted, and the results are shown in Table 3. An additional pictorial view of the estimates is provided in the Appendix section (Figure A1) to compare the different model estimates. The data had approximately 10% of missing information in the explanatory covariates. There was no difference in the model estimates between imputed data and complete case data (model 1 vs model 2 and model 4 vs model 5). Comparing the MLE (model 3) and the BE (model 4) models, the estimates were almost similar for most regression parameters; and inclusion of the individual random effects did not improve the model estimates (model 4 vs model 6). Lastly, adjusting for the competing effects of mortality on LTFU showed some significant improvement to the model based on the reduced AIC and BIC values compared to the Cox proportional hazard model. Hence, the competing risk model was considered a better fit compared to other models; and the interpretation of the results will be based on this model.

The multivariable competing risk model revealed that patient factors predicting LTFU included: being enrolled at a provincial/referral (AsHR 1.22; 95%CI: 1.18-1.26) or district/mission (AsHR 1.47; 95%CI: 1.45-1.50) hospitals (reference: PHC), urban sites (AsHR 1.61; 95%CI:1.59-1.63) (reference: rural) and 15-24 years group (AsHR 1.19; 95%CI: 1.16-1.21) (reference: 35-44 years). We also detected an overwhelming association between LTFU and tuberculosis-infected patients (AsHR 1.53; 95%CI: 1.45-1.62) (reference: no tuberculosis) and a protective effect of LTFU for patients classified in WHO stage III or IV (AsHR 0.74; 95%CI: 0.73-0.75) (reference: WHO I/II). Patients who were taking stavudine-combination therapy as a first-line ART treatment had an increased risk of becoming LTFU relative to patients taking TDF+3TC+EFV first-line combination therapy, that is, D4T(30)+3TC+NVP (AsHR 5.47; 95%CI:5.52-5.75) and D4T(30)+3TC+EFV (AsHR 7.98; 95%CI: 6.41-9.94). We also observed that patients who initiated ART between 2015 and 2017 had a pronounced risk of becoming LTFU compared to patients who enrolled between 2012 and 2014 (AsHR 6.02; 95%CI: 5.91-6.12).

For peer review only - http://bmjopen.bmj.com/site/about/guidelines.xhtml

Table 3: Multiple regression models results of LTFU amongst ART patients in Zimbabwe national ART programme, 2004-2017 for different models

**Characteristics**	**Category**	**Cox proportional hazard**		**Parametric Gompertz model**			**Weibull model with random effects**	**Competing risk model of mortality on LTFU**
		**CC + MLE (model 1)**	**MI+ MLE (model 2)**	**CC+BE (model 3)**	**CC+MLE (model 4)**	**MI+MLE (model 5)**	**CC+MLE (model 6)**	**CC+MLE (model 7)**
Health facility level	Primary health care	1(reference)	1(reference)	1(reference)	1(reference)	1(reference)	1(reference)	1(reference)
	Secondary hospitals[a]	1.48(1.46-1.50)	1.51(1.49-1.53)	1.53(1.51-1.54)	1.53(1.51-1.55)	1.57(1.54-1.59)	1.48(1.45-1.51)	1.47(1.45-1.50)
	Tertiary hospitals[b]	1.17(1.14-1.21)	1.23(1.20-1.27)	1.23(1.21-1.25)	1.23(1.20-1.27)	1.30(1.26-1.34)	1.10(1.06-1.14)	1.22(1.18-1.26)
Age (years) at ART initiation	15-24	1.16(1.13-1.19)	1.17(1.14-1.19)	1.12(1.11-1.14)	1.13(1.10-1.15)	1.14(1.12-1.17)	1.23(1.20-1.27)	1.19(1.16-1.21)
	25-34	1.18(1.17-1.20)	1.19(1.17-1.21)	1.18(1.16-1.19)	1.18(1.16-1.20)	1.19(1.17-1.20)	1.24(1.21-1.27)	1.19(1.17-1.21)
	35-44	1(reference)	1(reference)	1(reference)	1(reference)	1(reference)	1(reference)	1(reference)
	45+	0.88(0.87-0.89)	0.87(0.86-0.89)	0.88(0.87-0.89)	0.88(0.86-0.90)	0.87(0.85-0.88)	0.86(0.84-0.88)	0.87(0.85-0.89)
Degree of urbanisation	Rural	1(reference)	1(reference)	1(reference)	1(reference)	1(reference)	1(reference)	1(reference)
	Urban	1.70(1.68-1.72)	1.67(1.65-1.27)	1.67(1.65-1.69)	1.67(1.65-1.70)	1.64(1.61-1.66)	2.06(2.01-2.10)	1.61(1.59-1.63)
Calendar time of starting ART	2004-2007	0.04(0.04-0.05)	0.04(0.04-0.05)	0.02(0.02-2.02)	0.02(0.01-0.02)	0.02(0.01-0.02)	0.01(0.01-0.01)	0.09(0.09-0.10)
	2008-2011	0.07(.0.7-0.08)	0.07(0.07-0.08)	0.04(0.04-0.04)	0.04(0.04-0.04)	0.05(0.04-0.05)	0.02(0.02-0.02)	0.11(0.10-0.11)
	2012-2014	1(reference)	1(reference)	1(reference)	1(reference)	1(reference)	1(reference)	1(reference)
	2015-2017	8.48(8.34-8.63)	8.40(8.26-8.54)	5.95(5.90-6.00)	5.97(5.88-6.06)	5.90(5.81-5.99)	16.09(15.56-16.63)	6.02(5.91-6.12)
WHO staging at ART initiation	I/II	1(reference)	1(reference)	1(reference)	1(reference)	1(reference)	1(reference)	1(reference)
	III/IV	0.73(0.72-0.74)	0.74(0.73-0.75)	0.73(0.73-0.74)	0.73(0.72-0.75)	0.74(0.73-0.75)	0.67(0.65-0.68)	0.74(0.73-0.75)
Treatment regimen at the time of data extraction	D4T(30)+ 3TC+NVP	5.96(5.72-6.21)	6.00(5.77-6.25)	6.065.92-6.22)	6.09(5.84-6.34)	6.06(5.82-6.30)	15.05(13.98-16.21)	5.47(5.52-5.75)
	D4T(30)+ 3TC+EFV	8.55(7.21-10.12)	8.34(7.09-9.90)	8.71(8.21-9.20)	8.47(7.16-10.03)	8.29(7.01-9.79)	19.88(15.23-25.87)	7.98(6.41-9.94)
	AZT+3TC+NVP	0.73(0.67-0.80)	0.72(0.66-0.79)	0.74(0.71-0.78)	0.74(0.67-0.81)	0.72(0.65-0.79)	0.74(0.66-0.83)	0.75(0.68-0.83)
	AZT+3TC+EFV	1.19(1.02-1.40)	1.19(1.01-1.39)	1.19(1.16-1.23)	1.21(1.03-1.42)	1.21(1.03-1.40)	1.27(1.03-1.56)	1.09(0.91-1.30)
	TDF+3TC+NVP	2.41(2.36-2.47)	2.46(2.40-2.51)	2.46(2.42-2.50)	2.45(2.40-2.51)	2.50(2.44-2.55)	3.58(3.45-3.70)	2.29(2.23-2.34)
	TDF+3TC+EFV	1(reference)	1(reference)	1(reference)	1(reference)	1(reference)	1(reference)	1(reference)
	Other first lines	1.97(1.90-2.04)	1.97(1.91-2.04)	1.88(1.83-1.93)	1.89(1.82-1.95)	1.89(1.83-1.96)	2.44(2.32-2.57)	1.61(1.54-1.68)
	Second line	0.46(0.42-0.49)	0.45(0.42-0.49)	0.45(0.43-0.47)	0.44(0.41-0.48)	0.44(0.41-0.47)	0.39(0.36-0.42)	0.48(0.44-0.52)
Tuberculosis status at ART initiation	Negative	1(reference)	1(reference)	1(reference)	1(reference)	1(reference)	1(reference)	1(reference)
	Positive	2.14(2.05-2.24)	2.13(2.04-2.23)	2.03(1.99-2.09)	2.02(1.94-2.11)	2.01(1.93-2.10)	2.66(2.50-2.83)	1.53(1.45-1.62)
	Not assessed	0.58(0.56-0.59)	0.59(0.57-0.61)	0.60(0.59-0.60)	0.60(0.58-0.62)	0.61(0.59-0.63)	0.51(0.49-0.53)	0.60(0.58-0.62)
Body functional status at ART initiation	Working	1(reference)	1(reference)	1(reference)	1(reference)	1(reference)	1(reference)	1(reference)
	Ambulatory	1.22(1.19-1.24)	1.23(1.20-1.26)	1.23(1.21-1.26)	1.23(1.20-1.27)	1.25(1.22-1.28)	1.28(1.23-1.32)	1.08(1.05-1.11)
	Bed-ridden	1.34(1.17-1.53)	1.33(1.16-1.52)	1.32(1.29-1.35)	1.34(1.16-1.53)	1.32(1.15-1.51)	1.42(1.18-1.72)	0.64(0.54-0.75)
	AIC	196416.4			379948.9		445559.7	**195708.7**
	BIC	196439.1			380197.6		445819.3	**195731.4**
	DIC			379925.9				

CC=complete case, MI=multiple imputation, BE=Bayesian estimation, MLE=maximum likelihood estimation, Bold face= low values of the information criterion

[a] District and mission hospitals; [b] Provincial and referral hospitals

For peer review only - http://bmjopen.bmj.com/site/about/guidelines.xhtml

DISCUSSION

This study aims to determine the loss to follow-up (LTFU) rates at different health care levels after ART services decentralisation among ART patients who initiated ART between 2004 and 2017 and determine other potential independent factors using the competing risk model approach. Our study followed similar studies adjusting for the competing effect of mortality on LTFU, recognising that those who die are no longer at risk of LTFU [27,28]. We found the LTFU rate of 5.7 per 100- person-years, which was lower than that reported in Ethiopia of 8.2 per 100 person-years [29]. The lower LTFU rates in our study put forward the positive impact of the decentralised models implemented in Zimbabwe to increase patients' retention in HIV care. However, there is still a considerable magnitude of LTFU outcome in ART programs contributing to the "leakages" or attrition of patients within the continuum of care cascade in Zimbabwe. This requires continual upscaling of interventions which minimise the LTFU rates and retain patients in care since patients who are LTFU are associated with increased treatment interruptions which can trigger ART treatment defaulting, treatment non-adherence, treatment failure or treatment resistance [30,31].

Similar to other studies, LTFU independently increased at higher levels of care (provincial and referral (AsHR 1.22; 95%CI: 1.18-1.26) and district and mission (AsHR 1.47; 95%CI: 1.45-1.50) hospitals) relative to PHC in this study [5]. This finding supports the fact that decentralisation is associated with better patients' retention in care as there are high risks of LTFU rate in higher levels of care compared to the PHC facilities. This finding supports the assumption that if patients seek care from health facilities closer to their homes with cheaper transport cost and less congested hospital environments, there is improved patient retention as compared to seeking care to faraway health facilities [32]. Moreover, the high rates of LTFU in secondary and tertiary/quaternary levels of care could be explained by "silent transfers" whereby patients self-transfer themselves to these decentralised PHC facilities based on individual preferences of where to seek care, and the system fails to track it. These "silent transfers" could be a good indication of the upsurge of the decentralisation of services [32]. However, such "silent transfers" may result in inflated LTFU rates in higher levels of care as most these patients would be classified as LTFU when in fact they might have self-transferred and the patient tracking

system is not efficient enough to pick such scenarios due to significantly high patients' volumes persisting at these health facilities [5]. Similarly, the quarterly trend analysis of LTFU rates showed an increase over time, peaking in the period when decentralised models were implemented. The same issue of "silent transfers" of patients to nearer health facilities can also explain this trend pattern finding.

On the contrary, not everyone would prefer to seek care from these decentralised health facilities. Some may intentionally avoid these nearby health facilities to conceal their HIV status from the community. These individuals remain enrolled in faraway health facilities and most probably the higher levels of care. These individuals may contribute to the observed high rated of LTFU in this cohort as they may still face transport cost challenges to replenish their ART supplies[33]. Moreover, tertiary and quaternary levels of care may receive much sicker patients with a high risk of death, hoping to get better treatment through the health care referral system [34]. This means some of these much ill patients are more likely to become LTFU eventually as severe immune deterioration hinders one to keep up to date with their treatment replenishments. Earlier studies have also reported that much sicker patients with severe immune deterioration are more likely to become LTFU compared to those with no significant immunodeficiency [35]. Other studies have also shown that a significant portion of these severely ill patients who eventually become LTFU from higher levels of care health facilities, if tracked, could be found dead [36]. Our findings put forward that though the decentralisation uptake might have been taken up by the majority of the ART patients and improved patient's retention in the ART program in overall; those groups of individuals who still seek health care from the higher levels of care are still more at risk of becoming LTFU; hence, interventions which may retain patients in the higher levels of care facilities are a priority.

Patients enrolled at urban health facilities had increased risk to become LTFU compared to those at rural sites. This finding contradicts a study done in Ethiopia whereby rural residents had an increased risk of LTFU due to availability of transport costs, long distances to ART health facilities, social stigma as well as low levels of HIV knowledge in these communities[15]. However, our study finding can be explained by the decentralisation of health facilities to walkable distances in favour of rural residents [4,37]. Mobility of residents in urban areas is

For peer review only - http://bmjopen.bmj.com/site/about/guidelines.xhtml

disproportionate relative to rural residents; hence, most of these LTFU in urban areas could be part of the silent transfers to other nearer ART health facilities in their relocated areas.

Another interesting finding was high LTFU among the 15-24 years group. The adolescent's group is known to be associated with additional intricacy in their HIV management due to different types of chronic comorbidities, and this whole complexity may impact on their ART outcomes including LTFU [38,39]. The high risk of becoming LTFU among adolescents can be attributed to structural deprivation due to medical and psychological reasons which contribute to poor adherence in this subpopulation group as a result of becoming LTFU [40]. The most common medical barrier which has been found to affect ART adherence include treatment side effects, multiple doses and health system dissatisfaction. Research has found that ART has improved perinatal HIV infection into a controllable disease through optimal ART adherence. This opens up an additional challenge among adolescents who were prenatally or perinatally infected in the transition stage to adults care to experience ART adherence lapses and develop worse clinical outcomes as they become LTFU [40]. The transition from the paediatric care to adult care would mean changing care providers, absence of youth-friendly facilities, tight and hectic schedules, new responsibilities and deprivation of caregiver support for some of these adolescents. Some of the social deprivation issues among adolescents include inadequate financial support to cover transport costs to health facilities for treatment refill since this group heavily depends on their caregivers [41]. Some of the social deprivations faced by adolescents include lack of family support as some adolescents come from disrupted family structures with depraved caregiver-child relationships; fear perceived HIV-related stigma and discrimination to disclose their status [15,41,42].

Therefore, this study recommends the policymakers to strengthen the social protection support policy which addresses complex vulnerability, disadvantages and risks faced by HIV positive adolescents. This strategy also may foster resiliencies in general, HIV positive adolescents' population. The social protection support can interrupt some of the social deprivations faced by adolescents through poverty reduction and economic development, improved access to health care, access to health services, reduced stigma and discrimination and improved caregiver psychosocial and physical well-being. Another recommendation is the use of health facility-based peer-supporters programmes for adolescents to improve ART adherence and ultimately,

For peer review only - http://bmjopen.bmj.com/site/about/guidelines.xhtml

viral suppression[43,44]. The peer support activities involve peer-to-peer counselling, peer support groups and treatment buddy programmes among adolescents with the aim of ART adherence and ultimately achieving viral suppression, reducing the HIV-related illnesses among adolescents. As adolescents may exit the HIV care through becoming LTFU or withdrawals due to transition adjustments challenges from paediatric care to adult care; an early multidisciplinary and developmentally suitable transition preparedness mechanism is an alternative. The mechanism should be a comprehensive package addressing inherent adolescents' health issues like cognitive health, ART adherence, stigma, disclosure and socioeconomic issues [38,44]. Besides, a simple regimen, directly observed therapy and technological interventions like cell phone reminders have been found to increase ART adherence in adolescents.

We observed an overwhelming association of tuberculosis infection and becoming LTFU which concurs with an earlier study which reported that the risk of becoming LTFU was two times more likely in HIV patients with tuberculosis infection compared to those who did not have tuberculosis [29]. This finding can be explained by overlapping toxicity of HIV and tuberculosis drugs, leading to adverse reactions which have a direct consequence of becoming LTFU [29]. Therefore, a push towards integrated care of HIV and tuberculosis service delivery would elevate HIV uptake and tuberculosis screening and reduce travel inconveniences on patients [34,45]. Also, tuberculosis treatment before ART initiation reduces the risk to LTFU [42]; hence, it is profound to increase efforts in tuberculosis screening and treatment of HIV patients. However, issues around tuberculosis drug resistance cannot be ignored. Nowadays, HIV studies also consider information on non-communicable diseases like cancer, diabetes and hypertension; however, this information was missing from this current study database.

We also detected that patients classified in WHO stage III or IV were less likely to become LTFU relative to those classified in WHO stage I or II. However, our finding contradicts with earlier studies which have reported that experiencing a severe AIDS-defining ailment was associated with reduced risk of becoming LTFU[27,46]. Our study finding may put forward the fact that there could have been an upsurge of health-seeking behaviour amongst patients with advanced WHO clinical stages in this database cohort or this finding could be attributed to expansions of community awareness through health promotion in these health facilities communities.

For peer review only - http://bmjopen.bmj.com/site/about/guidelines.xhtml

Strengths and weaknesses of this study

This study highlights several strengths. A regularisation variable selection technique was used to identify important covariates. The lasso model shrinks the variable coefficient to zero and produces a simpler model which can be interpreted easily. For the survival analysis, the standard Cox regression model overestimated the risk in the presence of competing risks, and we used the Fine and Gray competing risk model instead [47]. Ignoring competing risks alter the probabilities of occurrence of the outcome giving biased estimates. Standard survival methods normally used in many studies censor the competing risk; however, this is improper because those who have died can no longer become LTFU. Such analysis will either overestimate or underestimate the risk ratios. We also used individual data which enabled exploration and adjustment for patient-level covariates associated with the outcome to get robust information on the ART outcome patterns as compared to aggregated data[33,48]. The study follow-up period was relatively long (13years), and the data came from all the ten provinces in Zimbabwe without restriction to specific geographical areas. The data represented quaternary, tertiary, secondary and primary health facilities from private and public sectors. This makes our results generalisable to the whole of Zimbabwe since the data reflect a true representation of all PLWHIV in the country. To the best of our knowledge, this study has used one of the largest datasets to analyse adult ART outcomes at the patient-level.

Our study results should be inferred in light of some limitations. Missing data was of concern, particularly to those variables that influence LTFU like viral load and CD4 cell count in addition to non-communicable diseases comorbidities. The presents of missing data could be explained by poor documentation of information [34] or limited access to the point of care machines for viral load, and CD4 cell counts measurements [4,49,50]. The missing patterns for some covariates like viral load and CD4 cell count measurements were likely to be informative and missing not at random (MNAR) since differentiated care monitoring approach was used in some years covered by this study[51]. Therefore, to maintain sufficient power, covariates with a high percentage of missing observations were excluded at the multivariable analysis stage. In future, we recommend that the data clerks should be trained on the importance of capturing full information and to the programme managers to consider expanding the HIV routine data collection tool to capture non-communication diseases conditions of the HIV patients. The socio-

For peer review only - http://bmjopen.bmj.com/site/about/guidelines.xhtml

demographic and clinical characteristics in the database were only taken at baseline or last observed at the time of data extraction; hence, we could not incorporate the time dependency of some covariates. We also acknowledge that there could be differential misclassification of the LTFU outcome in this study in the sense that some of those recorded as LTFU may be "silent transfers" who have enrolled in a different health facility as a result of ART decentralisation and have been given a different unique identifier number; hence, this might have biased our LTFU estimates in this study. There is also a possibility of participant selection bias since we only considered patients whose information as captured through the ePMS; as a result, the reported findings could be systematically different from those patients not included. However, it is of paramount importance to note that the dataset used was huge, and representation of the health facilities in Zimbabwe was reflected; hence, our findings could be generalised to all ART services health facilities in Zimbabwe.

While our findings for the common predictors of LTFU may be comparable to earlier studies conducted in Sub- Saharan Africa region, we had also hypothesised that those ART patients' who enrolled around 2008 would have a higher risk of becoming LTFU. This was done taking note of the hyperinflation that occurred in Zimbabwe during the part of the study period under review. The economic turmoil was allied to the increased mass departure of qualified health professionals and scarcities of vital medical supplies in health facilities [21,22]. Despite this situation, we detected a protective effect of becoming LTFU between 2008 and 2011 in this study. This could be explained by the support the country received through Global Fund [4], and ART sites increased from 282 sites in 2008 to 1,006 in 2012 [37]. For further studies, we recommend the use of time-varying covariates like regimen type, CD4 cell counts, WHO stages and viral load measurements; and the use of spatial analysis of ART outcome to identify high burdened regions which the policymakers may use to allocate resources, especially in resource-constrained settings.

Conclusion

The results from this current study using the data spanning over 13 years agree with the evident decline of the HIV incidence and prevalence in Zimbabwe; and the findings are comparable to similar HIV studies' findings, even from Demographic Health Survey [52]. Our results indicate

For peer review only - http://bmjopen.bmj.com/site/about/guidelines.xhtml

that patient retention in care is still a drawback in HIV prevention and control. Policymakers and program managers should come up with strategies which may retain patients on ART including increased patients surveillance and strengthen patients' tracing mechanisms to reduce LTFU at health facilities; strengthening community-based caregiver support [14], use of peers and nutritional aid support especially for adolescents and young (15-34years). In addition to interventions which minimise "leakages" in the HIV treatment cascade including use of ART adherence clubs strategies to reduce ART resistance [53] and sexual abstinence, campaigns should remain the cornerstone of HIV prevention and control. As much as most of the observed LTFU outcome at higher levels of care (district, provincial and central hospitals) could be "silent transfers", most patients who become LTFU are likely to have poor ART adherence if not dead. Poor ART adherence leads to poor viral suppression which affects achieving the third 90% UNAIDS targets, which state that 90% of those patients on ART should be virally suppressed. Clinicians should provide continual educational support on the importance of ART adherence to increase life expectancy among the HIV population and other benefits of ART. More important than not meeting the UNAIDS targets is the impact that poor viral suppression has on patients' health.

Supplementary materials

Additional theoretical aspects of survival models: Cox regression model and Competing risk model

[Insert Appendix 1-supplementary file here]

Hazard ratio plots for the different survival models fitted on complete case data and imputed time to LTFU data

[Insert Appendix 2- Figure A1 here]

Figure A1: Hazard ratio plots for the different survival models fitted on the complete case data and imputed time to becoming LTFU data among ART patients.

Author Contributions: **ZMZ** conceptualised the concept, cleaned and analysed the data and wrote the initial draft of the manuscript**. JT** and **TFC** advised on analysis, **EM** guided and

For peer review only - http://bmjopen.bmj.com/site/about/guidelines.xhtml

oversaw the analysis and reviewed the manuscript. **ZMZ, JT, TFC and EM** contributed to the interpretation of the results. All the authors reviewed the final manuscript for submission.

Funding: This work was supported by the Developing Excellence in Leadership, Training and Science (DELTAS) Africa Initiative Sub-Saharan Africa Consortium for Advanced Biostatistics (SSACAB) [Grant No. 107754/Z/15/Z]. The DELTAS Africa Initiative is an independent funding scheme of the African Academy of Sciences (AAS) Alliance for Accelerating Excellence in Science in Africa (AESA) and is supported by the New Partnership for Africa's Development Planning and Coordinating Agency (NEPAD Agency) with funding from the Wellcome Trust [Grant No. 107754/Z/15/Z] and the United Kingdom government. The views expressed in this publication are those of the authors and not necessarily those of the AAS, NEPAD Agency, Wellcome Trust, the UK government or the Ministry of Health and Child Care, Zimbabwe.

Acknowledgements: Our acknowledgements go to the Ministry of Health and Child Care, AIDS/TB Units Department for the support in data compilation and extraction for this study. We also thank the Division of Epidemiology and Biostatistics at the School of Public Health for their assistance in the getting ethical approval of this study. Thanks to Dr Nomathemba Chandiwana for technical assistance of this work.

Conflict of interest statement: All authors declare that they do not have a conflict of interest for this work.

Data sharing statement: The data used for this study can be found from a third party through an application process to the Zimbabwe Ministry of Health and Child Care through the HIV/AIDS Unit which oversees the data collection and compilation process for the ART program; therefore, the data is not publicly available.

Figure legends

Figure 1: Flowchart of the inclusion criteria for analyses of adult ART patients' enrolled in Zimbabwe ART program followed-up between 2004 and 2017

Figure 2: Schematic illustration of the variable of importance selection steps and subsequent models fitted for sensitivity analysis of the results

For peer review only - http://bmjopen.bmj.com/site/about/guidelines.xhtml

Figure 3: Trend analysis of loss to follow-up outcome among patients receiving ART in Zimbabwe National ART Programme on a quarterly time scale, 2004 to 2017

Figure 4: The cumulative incidence probability of mortality and loss to follow-up (A); and Kaplan-Meier curves for loss to follow-up stratified by baseline age categories (B), gender (C), level of urbanisation of the health facilities (D), baseline tuberculosis infection status (E) and baseline World Health Organization clinical stages for ART patients from the Zimbabwe national ART Programme electronic patient monitoring data, 2004 to 2017

REFERENCES

[1] UNAIDS. Global AIDS UPDATE 2016. UNAIDS 2016;17 Suppl 4:S3-11. http://www.who.int/hiv/pub/arv/global-aids-update-2016-pub/en/ [Accessed on 21 January 2018].

[2] Geng EH, Hare CB, Kahn JO, Jain V, Nunnery T Van, Christopoulos KA, et al. The Effect of a " Universal Antiretroviral Therapy " Recommendation on HIV RNA Levels Among HIV-Infected Patients Entering Care With a CD4 Count Greater Than 500 / μL in a Public Health Setting. Clinical Infectious Disease 2012;55:1690–7.

[3] Kitahata MM, Gange SJ, Abraham AG, Merriman B, Saag MS, Justice AC, et al. Effect of Early versus Deferred Antiretroviral Therapy for HIV on Survival. The New England Journal of Medicine 2009;360:1815–26.

[4] Mutasa-Apollo T, Shiraishi RW, Takarinda KC, Dzangare J, Mugurungi O, Murungu J, et al. Patient retention, clinical outcomes and attrition-associated factors of HIV-infected patients enrolled in Zimbabwe's National Antiretroviral Therapy Programme, 2007-2010. PLoS ONE 2014;9:2007–10.

[5] Rachlis B, Cole DC, van Lettow M, Escobar M, Muula AS, Ahmad F, et al. Follow-Up Visit Patterns in an Antiretroviral Therapy (ART) Programme in Zomba, Malawi. PLoS ONE 2014;9:e101875.

[6] Farahani M, Vable A, Lebelonyane R, Seipone K, Anderson M, Avalos A, et al. Outcomes of the Botswana national HIV/AIDS treatment programme from 2002 to 2010: a longitudinal analysis. The Lancet Global Health 2014;2:e44-50.

[7] Franke MF, Kaigamba F, Socci AR, Hakizamungu M, Patel A, Bagiruwigize E, et al. Improved Retention Associated With Community-Based Accompaniment for Antiretroviral Therapy Delivery in Rural Rwanda. Clinical Infectious Diseases 2013;56:1319–26. doi:10.1093/cid/cis1193.

[8] Somi G, Keogh SC, Todd J, Kilama B, Wringe A, van den Hombergh J, et al. Low

For peer review only

For peer review only - http://bmjopen.bmj.com/site/about/guidelines.xhtml

mortality risk but high loss to follow-up among patients in the Tanzanian national HIV care and treatment programme. Tropical Medicine & International Health 2012;17:497–506.

[9] UNAIDS. 90-90-90 An ambitious treatment target to help end AIDS epidemic. United Nations 2014. http://www.unaids.org/sites/default/files/media_asset/90-90-90_en.pdf[Accessed 25 January 2019].

[10] Ministry of Health and Child Care, National AIDS Council. Global Aids Response Progress-Fact Track Commitment to End by 2030- Gam Zimbabwe Country Report 2017. UNAIDS 2017:1–24. http://www.unaids.org/sites/default/files/country/documents/ZWE_2018_countryreport.pdf [Accessed on 27 September 2018].

[11] Osika J, Altman D, Ekbladh L, Katz I, Nguyen H, Rosenfeld J, et al. ZIMBABWE HEALTH SYSTEM ASSESSMENT 2010. 2010.

[12] MINISTER OF HEALTH AND CHILD CARE. Zimbabwe Service Availability and Readiness Assessment 2015 Report. Ministry of Health and Child Care 2015:1–145. http://ncuwash.org/newfour/wp-content/uploads/2017/08/Zimbabwe-Service-Availability-and-Readiness-Assessment-Report.pdf [Accessed date 25 May 2020].

[13] Suthar AB, Rutherford GW, Horvath T, Doherty MC, Negussie EK. Improving antiretroviral therapy scale-up and effectiveness through service integration and decentralization. AIDS 2014;28:S175–85.

[14] Ferrand RA, Simms V, Dauya E, Bandason T, Mchugh G, Mujuru H, et al. The effect of community-based support for caregivers on the risk of virological failure in children and adolescents with HIV in Harare, Zimbabwe (ZENITH): an open-label, randomised controlled trial. The Lancet Child and Adolescent Health 2017;1:175–83.

[15] Megerso A, Garoma S, Eticha TT, Workineh T, Daba S, Habtamu Z, et al. Predictors of loss to follow-up in antiretroviral treatment for adult patients in the Oromia region, Ethiopia. HIV/AIDS - Research and Palliative Care 2016;8:83–92.

[16] UNAIDS. 2004 Report on the global AIDS epidemic: 4th global report. UNAIDS 2004:1–236. https://files.unaids.org/en/media/unaids/contentassets/documents/unaidspublication/2004/GAR2004_en.pdf [Accessed on 15 August 2020].

[17] Apollo T, Takarinda K, Mugurungi O, Chakanyuka C, Simbini T, Harries AD. A Report on the Zimbabwe Antiretroviral Therapy (ART) Programme Progress Towards Achieving MGD6 Target 6B: Achievement and Challenges. The Central African Journal of Medicine 2010;56:12–4.

[18] Ministry of Health and Child Care. Guidelines for Antiretroviral Therapy for the Prevention and Treatment of HIV in Guidelines for Prevention and Treatment of HIV in Zimbabwe 2013:1–88.

For peer review only - http://bmjopen.bmj.com/site/about/guidelines.xhtml

[19] Ministry of Health and Child Care. Zimbabwe Electronic Patient Management System Strategic Plan Document 2012:1–52.

[20] Fine JP, Gray RJ. A Proportional Hazards Model for the Subdistribution of a Competing Risk. Journal of the American Statistical Association 1999;94:496–509.

[21] World Health Organization. Issues and challenges - Progress on the Health-Related MDGs 2015. http://www.aho.afro.who.int/profiles_information/index.php/Zimbabwe:Issues_and_challenges_-_Progress_on_the_Health-Related_MDGs [Accessed 10 October 2018].

[22] Meldrum A. Zimbabwe's health-care system struggles on. Lancet 2008;371:1059–60.

[23] Rubin DB. Inference and missing data. Biometrika 1976;63:581–92. doi:10.1093/biomet/63.3.581.

[24] Little RJ. Calibrated Bayes: A Bayes/frequentist roadmap. American Statistician 2006;60:213–23. doi:10.1198/000313006X117837.

[25] Pourhoseingholi MA, Hajizadeh E, Moghimi Dehkordi B, Safaee A, Abadi A, Zali MR. Comparing Cox regression and parametric models for survival of patients with gastric carcinoma. Asian Pacific Journal of Cancer Prevention 2007;8:412–6.

[26] StataCorp. Stata Statistical Software: Release 15. College Station, Texas, USA 2017.

[27] Schöni-Affolter F, Keiser O, Mwango A, Stringer J, Ledergerber B, Mulenga L, et al. Estimating loss to follow-up in HIV-infected patients on antiretroviral therapy: The effect of the competing risk of death in Zambia and Switzerland. PLoS ONE 2011;6:2–8.

[28] Assemie MA, Muchie KF, Ayele TA. Incidence and predictors of loss to follow up among HIV ‑ infected adults at Pawi General Hospital, northwest Ethiopia : competing risk regression model. BMC Research Notes 2018;11:1–6.

[29] Tadesse K, Haile F. Predictors of Loss to Follow Up of Patients Enrolled on Antiretroviral Therapy : A Retrospective Cohort Study. AIDS & Clinical Research 2014;5:1–6.

[30] Babo YD, Alemie GA, Fentaye FW. Predictors of first-line antiretroviral therapy failure amongst HIV-infected adult clients at Woldia Hospital, Northeast Ethiopia. PLoS ONE 2017;12:e0187694.

[31] Dewing SF, Mathews C, Lurie M, Kagee A, Padayachee T, Lombard CJ, et al. Predictors of poor adherence among people on antiretroviral treatment in Cape Town, South Africa: A case-control study. AIDS Care 2015;27:342–9. doi:10.1080/09540121.2014.994471.Predictors.

[32] Geng EH, Odeny TA, Lyamuya R, Nakiwogga-Muwanga A, Diero L, Bwana M, et al. Retention in care and patient-reported reasons for undocumented transfer or stopping care among HIV-infected patients on antiretroviral therapy in Eastern Africa: Application of a sampling-based approach. Clinical Infectious Diseases 2016;62:935–44.

For peer review only - http://bmjopen.bmj.com/site/about/guidelines.xhtml

[33] Fatti G, Grimwood A, Bock P. Better antiretroviral therapy outcomes at primary healthcare facilities: An evaluation of three tiers of ART services in four South African provinces. PLoS ONE 2010;5:1–10.

[34] Molfino L, Kumar AM, Isaakidis P, Van den Bergh R, Khogali M, Hinderaker SG, et al. High attrition among HIV-infected patients with advanced disease treated in an intermediary referral center in Maputo, Mozambique. Global Health Action 2014;7:23758.

[35] Matsena Zingoni Z, Chirwa TF, Todd J, Musenge E. HIV Disease Progression Among Antiretroviral Therapy Patients in Zimbabwe : A Multistate Markov Model. Frontiers in Public Health 2019;7:1–15. doi:10.3389/fpubh.2019.00326.

[36] Fox MP, Rosen S. Patient retention in antiretroviral therapy programs up to three years on treatment in sub-Saharan Africa, 2007-2009: Systematic review. Tropical Medicine and International Health 2010;15:1–15.

[37] Ministry of Health and Child Care. National HIV Care and Treatment Strategic Plan 2013-2017 and Three Year Operational Plan 2013:1–80.

[38] McHugh G, Simms V, Dauya E, Bandason T, Chonzi P, Metaxa D, et al. Clinical outcomes in children and adolescents initiating antiretroviral therapy in decentralized healthcare settings in Zimbabwe. Journal of the International AIDS Society 2017;20:1–9.

[39] Ferrand RA, Bandason T, Musvaire P, Larke N, Nathoo K, Mujuru H, et al. Causes of acute hospitalization in adolescence: Burden and spectrum of HIV-related morbidity in a country with an early-onset and severe HIV epidemic: A prospective survey. PLoS Medicine 2010;7:1–11.

[40] Zanoni BC, Mayer KH. The adolescent and young adult HIV cascade of care in the United States: Exaggerated health disparities. AIDS Patient Care and STDs 2014;28:128–35. doi:10.1089/apc.2013.0345.

[41] Maskew M, Fox MP, Evans D, Govindasamy D, Jamieson L, Malete G, et al. Insights into Adherence among a Cohort of Adolescents Aged 12-20 Years in South Africa: Reported Barriers to Antiretroviral Treatment. AIDS Research and Treatment 2016;2016:1–13. doi:http://dx.doi.org/10.1155/2016/4161738.

[42] Alvarez-Uria G, Naik PK, Pakam R, Midde M. Factors associated with attrition, mortality, and loss to follow up after antiretroviral therapy initiation: Data from an HIV cohort study in India. Global Health Action 2013;6:1–8.

[43] PATA. The Dar es Salaam Call to Action – Peers to Zero. PATA 2016:6. http://teampata.org/wp-content/uploads/2017/10/Dar-Declaration_WEB.pdf[Accessed on 3 February 2020].

[44] Mark D, Lovich R, Walker D, Burdock T, Ronan A, Hatane L. Providing peer support for adolescents and young people living with HIV. Child Survival Working Group 2020:3–6. http://www.childrenandaids.org/sites/default/files/2018-07/12-ProvidingPeerSupport-CSWG.pdf[Accessed on 26 January 2020].

For peer review only - http://bmjopen.bmj.com/site/about/guidelines.xhtml

[45] Takarinda KC, Sandy C, Masuka N, Hazangwe P, Choto RC, Mutasa-Apollo T, et al. Factors Associated with Mortality among Patients on TB Treatment in the Southern Region of Zimbabwe, 2013. Hindawi Tuberculosis Research and Treatment Journal 2017;2017:1–11.

[46] Berheto TM, Haile DB, Mohammed S. Predictors of loss to follow-up in patients living with HIV/AIDS after initiation of antiretroviral therapy. North American Journal of Medical Sciences 2014;6:453–459.

[47] Andersen PK, Z SA, Susanne Rosthoj. Competing risks as a multi-state model. Statistical Methods in Medical Research 2002;11:203–15.

[48] Weigel R, Estill J, Egger M, Harries AD, Makombe S, Tweya H, et al. Mortality and loss to follow-up in the first year of ART. AIDS 2012;26:365–73.

[49] Layer EH, Kennedy CE, Beckham SW, Mbwambo JK, Likindikoki S, Davis WW, et al. Multi-level factors affecting entry into and engagement in the HIV continuum of care in Iringa, Tanzania. PLoS ONE 2014;9. doi:10.1371/journal.pone.0104961.

[50] Nyika H, Mugurungi O, Shambira G, Gombe NT, Bangure D, Mungati M, et al. Factors associated with late presentation for HIV / AIDS care in Harare City, Zimbabwe, 2015. BMC Public Health 2016;16:1–7. doi:10.1186/s12889-016-3044-7.

[51] Phillips A, Vojnov L, Cohn J, Roberts T, Ellman T, Bonner K, et al. Sustainable HIV Treatment in Africa through Viral Load-Informed Differentiated Care. Nature 2016;528:S68–S76.

[52] Zimbabwe National Statistics Agency. Zimbabwe Demographic and Health Survey. Statistics 2015:535. doi:10.1017/CBO9781107415324.004.

[53] Bock P, Gunst C, Maschilla L, Holtman R, Grobbelaar N, Wademan D, et al. Retention in care and factors critical for effectively implementing antiretroviral adherence clubs in a rural district in South Africa. Journal of the International AIDS Society 2019;22:e25396.

For peer review only

For peer review only - http://bmjopen.bmj.com/site/about/guidelines.xhtml

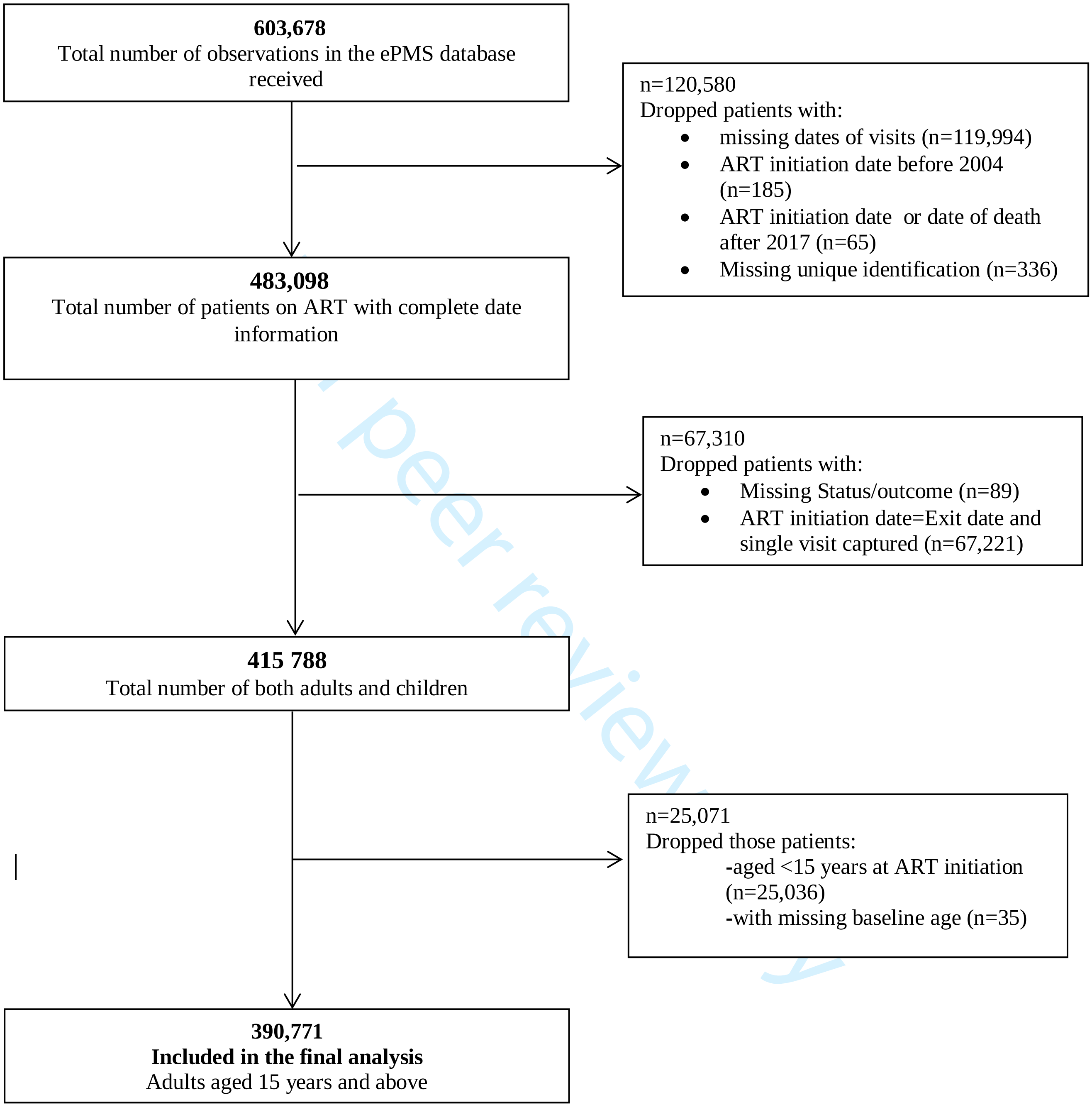

Figure 1: Flowchart of the inclusion criteria for analyses of adult ART patients' enrolled in Zimbabwe ART program followed-up between 2004 and 2017

For peer review only - http://bmjopen.bmj.com/site/about/guidelines.xhtml

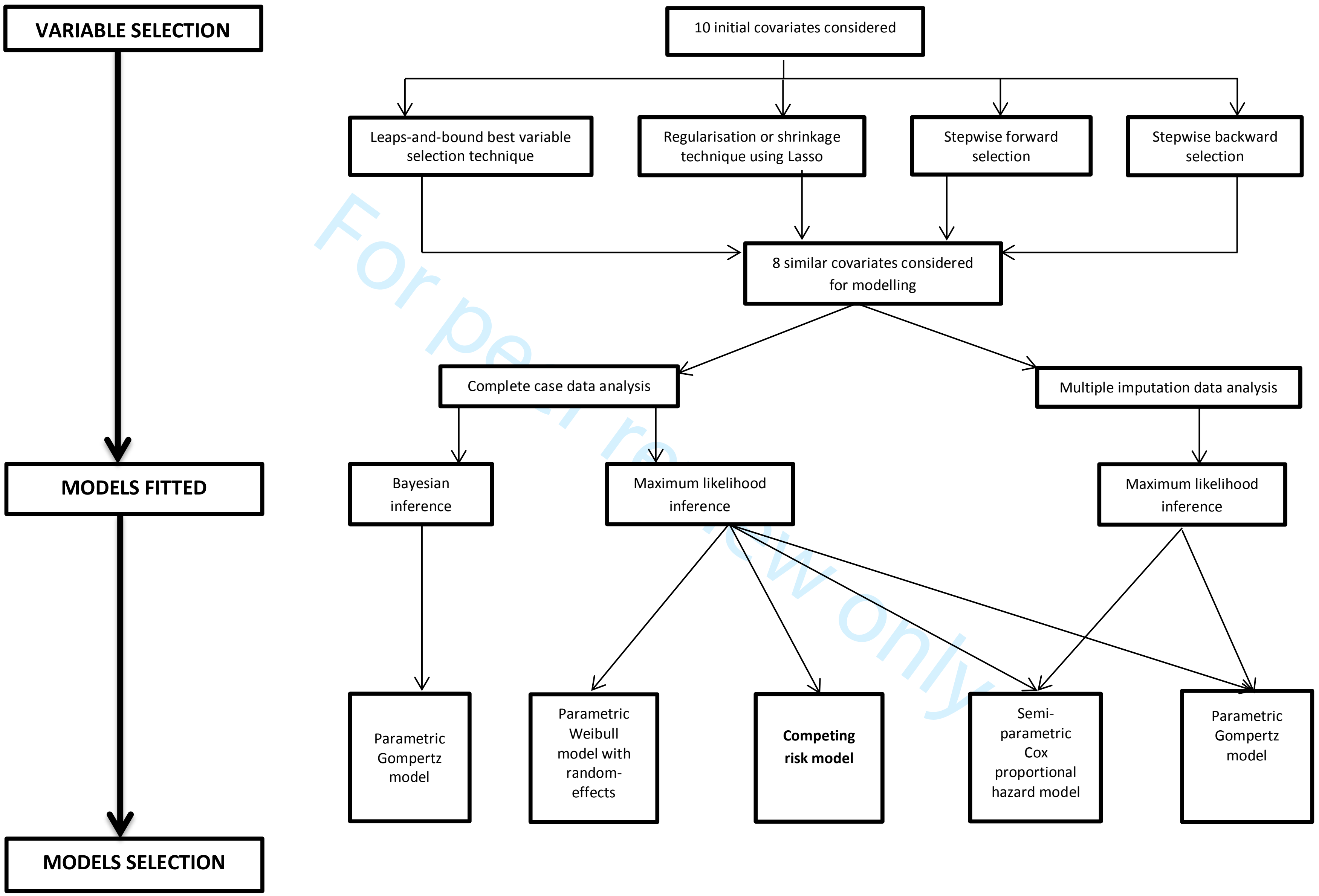

Figure 2: Schematic illustration of variable of importance selection steps and subsequent models fitted for sensitivity analysis of the results

For peer review only - http://bmjopen.bmj.com/site/about/guidelines.xhtml

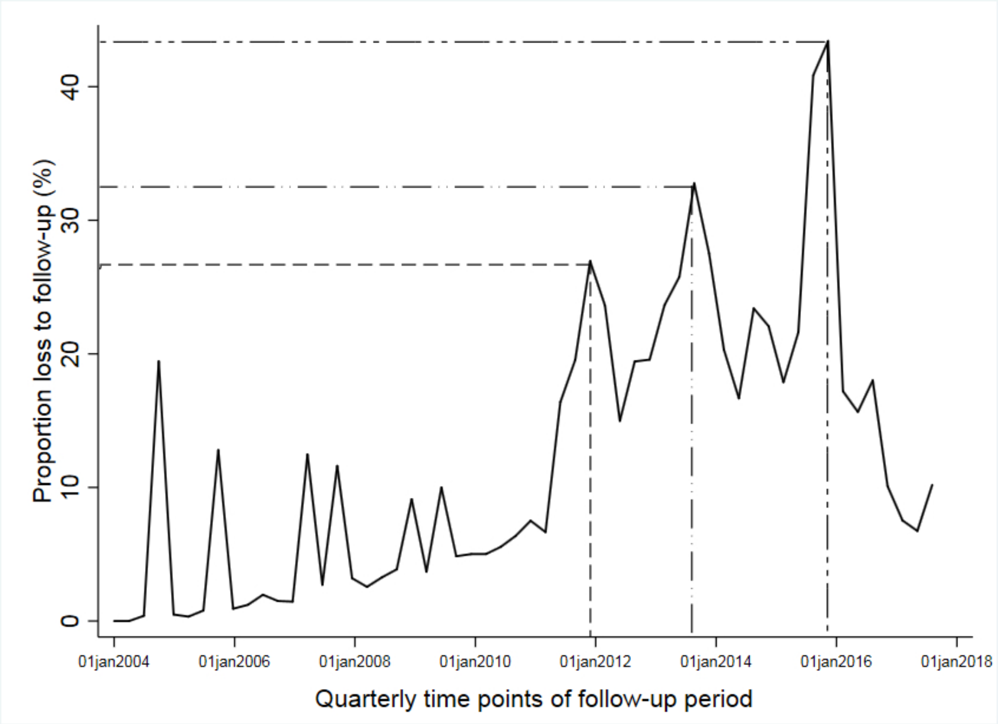

Figure 3: Trend analysis of loss to follow-up outcome among patients receiving ART in Zimbabwe National ART Programme on a quarterly time scale, 2004 to 2017

298x217mm (72 x 72 DPI)

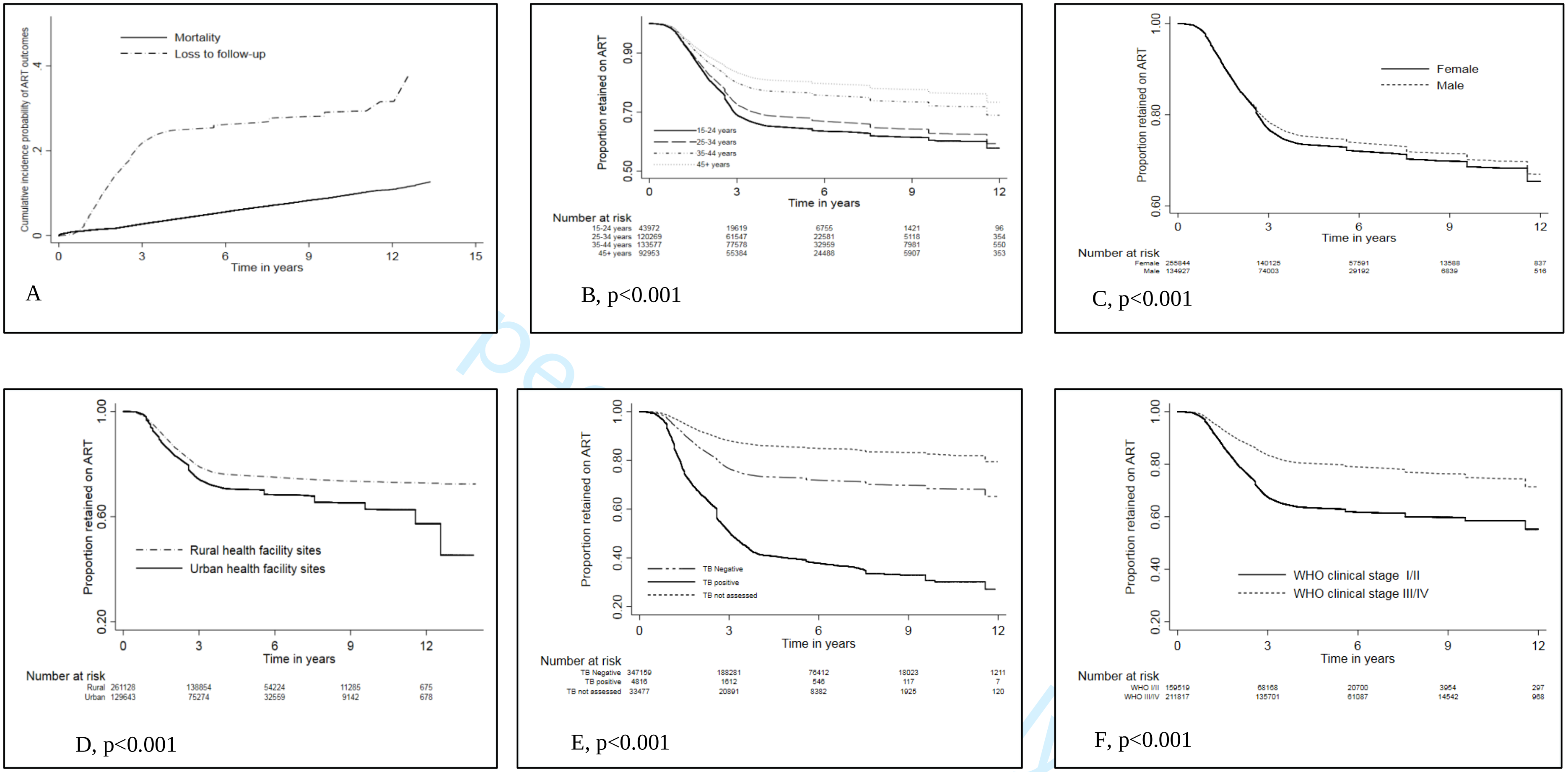

Figure 4: The cumulative incidence probability of mortality and loss to follow-up (A); and Kaplan-Meier curves for loss to follow-up stratified by baseline age categories (B), gender (C), level of urbanization of the health facilities (D), baseline tuberculosis infection status (E) and baseline World Health Organization clinical stages for ART patients from the Zimbabwe national ART Programme electronic patient monitoring data, 2004 to 2017

A1: Cox proportional hazard

Traditionally for time-to-event analysis, researchers tend to fit the semi-parametric cause-specific proportional hazard model which was proposed by Cox (1972) because of its less stringent assumption on the hazard form and survival function [22]. For the conventional cause-specific proportion hazard, we did a non-informative censoring of LTFU and transfer-out. We assumed that the hazard function is an arbitrary, unspecified, non-negative function of time [22].

A2: Survival Function

Let *T* be a non-negative random variable defining the survival time of an individual. The survival function [*S(t)*] is used to represent the probability that an individual survives from time origin to some time beyond *t*

$$S(t) = P(T \geq t) \quad (1)$$

If we assume that *T* is a continuous random variable $[P(T \geq t) = P(T > t)]$, the different values that *T* can take have an underlying probability density function (pdf) given by:

$$S(t) = P(T > t) = \int_0^t f(x)dx = 1 - F(t) \quad (2)$$

where *F(t)* is the cumulative distribution function (CDF) expressed as:

$$F(t) = P(T < t) = \int_0^t f(x)dx \quad (3)$$

If one has a survival function, the density function can be calculated by differentiating the cumulative distribution function as:

$$f(t) = -\frac{dF(t)}{dt} \quad (4)$$

A3: The Hazard function

The hazard function is the probability that an individual dies at time *t* conditional to having survived to that time. The hazard function, therefore, represents an instantaneous death (failure) rate for an individual who survived to that time. In other words, the hazard function considers the probability that a random variable is associated with an individual's survival time lies between *t* and $t + \delta t$ conditional to *T* being greater than or equal to *t*:

For peer review only

$$h(t) = P(t < T < t + \delta t \mid T \geq t) \\ = \frac{F(t+\delta t) - F(t)}{S(t)} \tag{5}$$

where $F(t)$ is the cumulative distribution function. The hazard function is the limiting value of the above probability in Equation 5 divided by the time interval δt, as δt tends to zero. This can be re-written as:

$$\begin{aligned} h(t) = \mu(t) &= \lim_{\delta t \to 0} \frac{P[t \leq T < t + \delta t \mid T \geq t]}{\delta t} \\ &= \lim_{\delta t \to 0} \frac{P[t \leq T < t + \delta t]}{\delta t P(T > t)} \\ &= \lim_{\delta t \to 0} \frac{F[t + \delta t - F(t)]}{\delta t S(t)} \\ &= \frac{1}{S(t)} \left\{ \lim_{\delta t \to 0} \frac{F[t + \delta t - F(t)]}{\delta t} \right\} \end{aligned} \quad . \tag{6}$$

But $\left\{ \lim_{\delta t \to 0} \frac{F[t + \delta t - F(t)]}{\delta t} \right\} = f(t)$ and since T is a continuous random variable, then

$$h(t) = \frac{f(t)}{S(t)} = -\frac{d}{dt}\log[S(t)] \ . \tag{7}$$

The cumulative hazard is

$$H(t) = \int_0^t h(u)du = -\log[S(x)] \ . \tag{8}$$

Thus for a continuous lifetimes

$$\begin{aligned} S(t) &= \exp\{-H(t)\} \\ &= \exp\left[-\int_0^t h(u)du\right] \end{aligned} \ . \tag{9}$$

A4: The Cox regression model

This model is also known as the Proportional hazard regression model, which models the incidence or hazard rate (mortality rate in our case). This model assumes independence of

survival times between distinct individuals in a sample and a constant hazard ratio regardless of time. The model can be written as:

$$\log\left(\frac{h(t)}{h_0(t)}\right) = \beta_1 X_1 + \beta_2 X_2 + \ldots + \beta_k X_k$$
$$\left(\frac{h(t)}{h_0(t)}\right) = \exp(\beta_1 X_1 + \beta_2 X_2 + \ldots + \beta_k X_k) \qquad (10)$$
$$h(t) = h_0(t)\exp(\beta_1 X_1 + \beta_2 X_2 + \ldots + \beta_k X_k)$$

This model can be simplified in a matric form as:

$$h(t/\mathbf{Z}) = h_0(t)\exp(\boldsymbol{\beta}\,\mathbf{Z}^T)$$

where $h(t/\mathbf{Z})$ is the expected hazard at time t conditional to the covariate matrix $\mathbf{Z}$. The expected hazard is a product of the baseline hazard ($h_0(t)$) (which is a function of duration time only) and the exponential function of the regressors ($\boldsymbol{\beta} = (\beta_1, \beta_2, \ldots, \beta_k)$; a 1 x k parameter vector) for k covariates. Then the explanatory variables for n persons, $\mathbf{Z}$ matrix, will be:

$$\mathbf{Z} = \begin{bmatrix} X_{11}, & X_{12}, & . & . & ., & X_{1n} \\ X_{21}, & X_{22}, & . & . & ., & X_{2n} \\ . & . & . & . & . & . \\ . & . & . & . & . & . \\ . & . & . & . & . & . \\ X_{k1}, & X_{k2}, & . & . & ., & X_{kn} \end{bmatrix}$$

Thus for the individual covariates, $Z_i = (X_{i1}, \ldots., X_{ik})^T$, the baseline hazard ($h_0(t)$ is equal to *h(t)* when all the regressors are equal to zero, the function of the regressors will be equal to 1. The Cox model is termed semi-parametric because it is made up of the parametric part for the covariates adjustment and the non-parametric baseline hazard as there are no assumptions for the underlying hazard function.

A5: Likelihood function of the Cox regression

The Cox model parameters are estimated by maximising the partial log-likelihood function. Letting the minimum censoring time C_j and survival time T_j be denoted by $t_{(j)}$. The censoring

For peer review only

time is right censored at C after observing that $T_j > C_j$. Let $R(t_j)$ denotes the set of lives which are at risk before the *j*th observed lifetime. Then the partial likelihood is a product of the event times of a quotient that compares the hazard of individuals with the event at a time $t_{(j)}$ to all the hazard of all individuals at risk at a time $t_{(j)}$ can be derived in terms of these censoring times. If we observe $\left\{\left(T_j,\delta_j\right)\right\}_{j=1}^{n}$ where T_j is still the observed time and δ_j the censoring indicator taking values 1 if the failure time is observed for individuals $j=1,2,...,n$ where n is the total number of observations and 0 if right censoring, then the likelihood can be easily expressed as a product of the pdf and the cumulative survival function expressed as $LH \propto \prod_{j=1}^{n} f\left(t_j\right)^{\delta_j} S\left(t_j +\right)^{1-\delta_j}$. This function can be expressed as well as a partial likelihood function in terms of covariates and regression coefficients matrices, as shown below:

$$L_p(\beta \mid D_{obs}) = \prod_{j=1}^{n}\left[\frac{\exp(\boldsymbol{Z}_j^T\boldsymbol{\beta})}{\sum_{k\in R(t_j)}\exp(\boldsymbol{Z}_k^T\boldsymbol{\beta})}\right]^{\delta_j}, \tag{11}$$

where $D_{obs} = \{(t_j, \delta_j, \boldsymbol{Z}): j=1,2,...,n\}$ is the observed univariate right-censored survival data. Considering the Breslow concept when ties are present, the partial likelihood may be expressed as:

$$L_{pt}(\beta \mid D_{obs}) = \prod_{j=1}^{d}\frac{\exp(\boldsymbol{Z}_j^T\boldsymbol{\beta})}{\left[\sum_{k\in R(t_j)}\exp(\boldsymbol{Z}_k^T\boldsymbol{\beta})\right]^{d_j}},$$

where $d=\sum_{j=i}^{n}\delta_j, \boldsymbol{z}_j = \sum_{k\in D_j}\boldsymbol{x}_j, d_j =$ the number of events at $t_{(j)}$ and $R(t_j)$ is a set of all individuals who have the event at the defined time $t_{(j)}$. Thus, this can be written as

$$L_{pt}(\beta \mid D_{obs}) = \prod_{j=1}^{n}\frac{\exp(\delta_j\boldsymbol{Z}_j^T\boldsymbol{\beta})}{\left[\sum_{k\in R(t_j)}\exp(\boldsymbol{Z}_k^T\boldsymbol{\beta})\right]^{\delta_j}},$$

For peer review only

Expanding the Breslow equation gives:

$$\log L(\beta \mid D_{obs}) = \sum_{j=1}^{D}\left[\sum_{i\in D_j}^{n} \boldsymbol{Z}_j^T \boldsymbol{\beta} - d_j \log\left\{\sum_{k\in R_j}^{n} \exp(\boldsymbol{Z}_j^T \boldsymbol{\beta})\right\}\right] \tag{12}$$

where j indexes the ordered failure times $t_{(j)}, j = 1,...,D$; D_j is the set of d_j observations that fail at time $t_{(j)}$; d_j is the number of failures at time $t_{(j)}$; and R_j is the set of observations k that are at risk at time $t_{(j)}$(that is, all k such that $t_{0k} < t_{(j)} \le t_k$). This can be simplified in set notation form as $k \in R_j$ and $d_j \in D_j$.

A6: Competing risk model specification [49]

In epidemiological studies, more than one cause of failure is possible, hence the estimates of the targeted outcome probabilities are either over-estimated with the Cox model [50] the drive for the competing risk analysis to get marginal probabilities of mortality as proposed by Fine and Gray [17]. Competing risk is when we have more than one cause of failure outcomes, and we are only interested in one of them. In this case, mortality was the competing event to LTFU. The Kaplan-Meier estimate is normally used, where the competing risk events are treated as censored observations, but this is biased since the independence assumption is violated. An individual who is censored because of the competing event will certainly not experience the outcome of interest; hence, the Kaplan-Meier may overestimate the probability of failure, especially if completion is high. Hence, using the Kaplan-Meier estimate in this situation would result in the biased estimates. The competing risk inference is the joint distribution of time of death or failure (T) and failure outcome, in our case death (D) conditional to some covariates, X. The cause-specific hazard (q cause of death) function will be

$$h_q(t) = \lim_{\delta x \to 0} \frac{P(t \le T < t + \delta t, D = q \mid T \ge t)}{\delta t} \tag{13}$$

The cumulative cause-specific hazard is written as:

$$H_q(t) = \int_0^t h_q(u)du \tag{14}$$

And define

$$S_q(t) = \exp(H_q(t)) \tag{15}$$

For peer review only - http://bmjopen.bmj.com/site/about/guidelines.xhtml

The cumulative incidence function (CIF) or the sub-distribution function for death is defined as:

$$I_q(t) = P(T \le t, D = q) = \int_0^t h_q(u) S(u) du \qquad (16)$$

This function is sub-distribution since the cumulative probability of death is always below one, that is, $I_q(\infty) = P(D = q) < 1$. The hazard specification would be $h_q(t) = \frac{d \log(1 - I_q(t))}{dt}$, and the hazard model is expressed as

$$h_q(t / \mathbf{Z}) = h_{q,0}(t) \exp(\boldsymbol{\beta}_q \mathbf{Z}^T) \qquad (17)$$

where $h_{q,0}$ is the baseline sub-distribution hazard of cause q and the vector $\boldsymbol{\beta}_q$ represents the matrix of covariates effects of cause q . To estimate the unknown $\boldsymbol{\beta}_q$ parameters, we maximise the following log-pseudolikelihood function:

$$\log L(\beta \mid D_{obs}) = \sum_{i=1}^{n} \delta_i w_i \left[\boldsymbol{Z}_i^T \boldsymbol{\beta} + offset_i - \log \left\{ \sum_{k \in R_i}^{n} w_i \alpha_{ik} \exp(\boldsymbol{Z}_k^T \boldsymbol{\beta} + offset_k) \right\} \right] \qquad (18)$$

where δ_i is death for an *i*th individual, R_i is the set of observations, q at risk at time t_i. The only difference with the Cox is the α_{iq} weights. These weights keep individuals who experience the competing event to be in subsequent risk sets and to reduce their weight over time hence their likelihood of being censored increases.

For peer review only

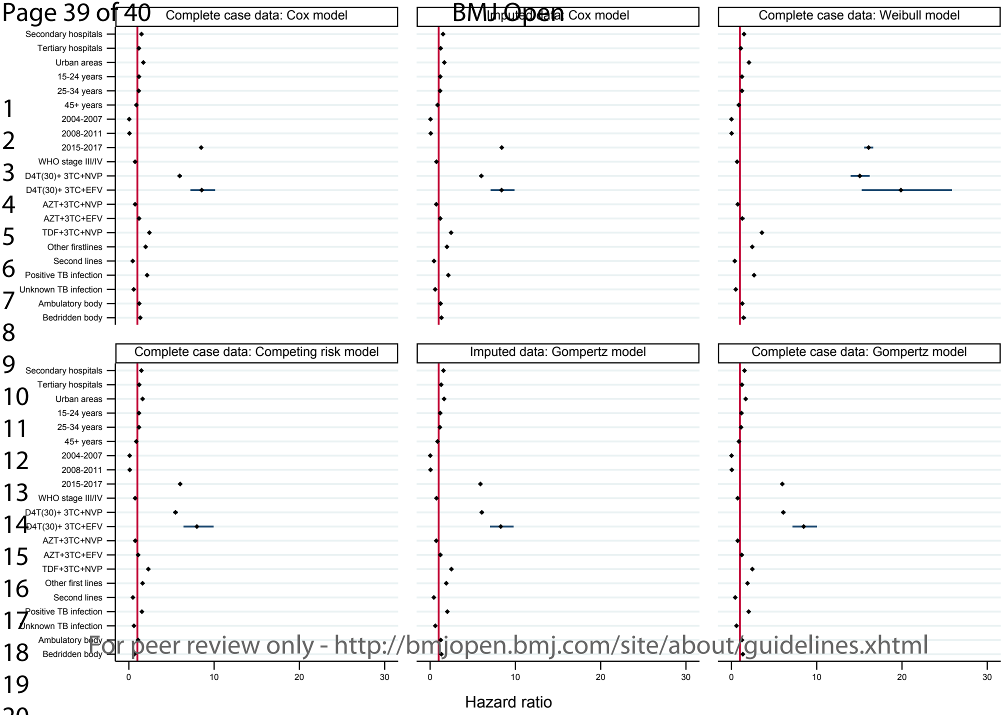

For peer review only - http://bmjopen.bmj.com/site/about/guidelines.xhtml

STROBE 2007 (v4) Statement—Checklist of items that should be included in reports of *cohort studies*

Section/Topic	Item #	Recommendation	Reported on page #
**Title and abstract**	1	(*a*) Indicate the study's design with a commonly used term in the title or the abstract	1
		(*b*) Provide in the abstract an informative and balanced summary of what was done and what was found	1-2
**Introduction**			
Background/rationale	2	Explain the scientific background and rationale for the investigation being reported	3
Objectives	3	State specific objectives, including any prespecified hypotheses	4
**Methods**			
Study design	4	Present key elements of study design early in the paper	4
Setting	5	Describe the setting, locations, and relevant dates, including periods of recruitment, exposure, follow-up, and data collection	4-5
Participants	6	(*a*) Give the eligibility criteria, and the sources and methods of selection of participants. Describe methods of follow-up	4
		(*b*) For matched studies, give matching criteria and number of exposed and unexposed	n/a
Variables	7	Clearly define all outcomes, exposures, predictors, potential confounders, and effect modifiers. Give diagnostic criteria, if applicable	5
Data sources/ measurement	8*	For each variable of interest, give sources of data and details of methods of assessment (measurement). Describe comparability of assessment methods if there is more than one group	
Bias	9	Describe any efforts to address potential sources of bias	5
Study size	10	Explain how the study size was arrived at	4
Quantitative variables	11	Explain how quantitative variables were handled in the analyses. If applicable, describe which groupings were chosen and why	4
Statistical methods	12	(*a*) Describe all statistical methods, including those used to control for confounding	5
		(*b*) Describe any methods used to examine subgroups and interactions	n/a
		(*c*) Explain how missing data were addressed	6
		(*d*) If applicable, explain how loss to follow-up was addressed	n/a
		(*e*) Describe any sensitivity analyses	6
**Results**			

For peer review only

Participants	13*	(a) Report numbers of individuals at each stage of study—eg numbers potentially eligible, examined for eligibility, confirmed eligible, included in the study, completing follow-up, and analysed	4
		(b) Give reasons for non-participation at each stage	n/a
		(c) Consider use of a flow diagram	4
Descriptive data	14*	(a) Give characteristics of study participants (eg demographic, clinical, social) and information on exposures and potential confounders	7-8
		(b) Indicate number of participants with missing data for each variable of interest	8
		(c) Summarise follow-up time (eg, average and total amount)	7
Outcome data	15*	Report numbers of outcome events or summary measures over time	7
Main results	16	(*a*) Give unadjusted estimates and, if applicable, confounder-adjusted estimates and their precision (eg, 95% confidence interval). Make clear which confounders were adjusted for and why they were included	10
		(*b*) Report category boundaries when continuous variables were categorized	n/a
		(*c*) If relevant, consider translating estimates of relative risk into absolute risk for a meaningful time period	n/a
Other analyses	17	Report other analyses done—eg analyses of subgroups and interactions, and sensitivity analyses	10
**Discussion**			
Key results	18	Summarise key results with reference to study objectives	12
**Limitations**			
Interpretation	20	Give a cautious overall interpretation of results considering objectives, limitations, multiplicity of analyses, results from similar studies, and other relevant evidence	12-15
Generalisability	21	Discuss the generalisability (external validity) of the study results	15
**Other information**			
Funding	22	Give the source of funding and the role of the funders for the present study and, if applicable, for the original study on which the present article is based	15

*Give information separately for cases and controls in case-control studies and, if applicable, for exposed and unexposed groups in cohort and cross-sectional studies.

Note: An Explanation and Elaboration article discusses each checklist item and gives methodological background and published examples of transparent reporting. The STROBE checklist is best used in conjunction with this article (freely available on the Web sites of PLoS Medicine at http://www.plosmedicine.org/, Annals of Internal Medicine at http://www.annals.org/, and Epidemiology at http://www.epidem.com/). Information on the STROBE Initiative is available at www.strobe-statement.org.

For peer review only
